# Supplementary material for: Readout of intrinsic and induced DNA shape by homeodomain transcription factor complexes
Source: Biophys J. 2026 Mar 20;125(16):4345–56. doi: 10.1016/j.bpj.2026.03.036 (PMC13119616; doi:10.1016/j.bpj.2026.03.036)
Supplement: Supplementary file 1 [file mmc1.pdf]

Biophysical Journal, Volume *125*

Supplemental information

Readout of intrinsic and induced DNA shape by homeodomain transcription factor complexes

Yibei Jiang, Alexandra M. Shewchuk, Tsu-Pei Chiu, Jinsen Li, Judith F. Kribelbauer-Swietek, Nicolas Gompel, and Remo Rohs

SUPPLEMENTAL INFORMATION

Readout of induced and intrinsic DNA shape by homeodomain transcription factor complexes

Yibei Jiang[1,†], Alexandra M. Shewchuk[1], Tsu-Pei Chiu[1], Jinsen Li[1], Judith F. Kribelbauer-Swietek[1,2], Nicolas Gompel[3], and Remo Rohs[1,4,5,6,7,8,*]

[1]Department of Quantitative and Computational Biology, University of Southern California, Los Angeles, CA 90089, USA

[2]Department of Biological Sciences, University of Southern California, Los Angeles, CA 90089, USA

[3]Bonn Institute for Organismic Biology, University of Bonn, 53115 Bonn, Germany

[4]Department of Chemistry, University of Southern California, Los Angeles, CA 90089, USA

[5]Department of Physics & Astronomy, University of Southern California, Los Angeles, CA 90089, USA

[6]Thomas Lord Department of Computer Science, University of Southern California, Los Angeles, CA 90089, USA

[7]Division of Medical Oncology, Department of Medicine, University of Southern California, Los Angeles, CA 90033, USA

[8]Alfred E. Mann Department of Biomedical Engineering, University of Southern California, Los Angeles, CA 90089, USA

[†]Present address: Illumina, Inc., 5200 Illumina Way, San Diego, CA 92122, USA

*To whom correspondence should be addressed. Tel: +1 213 740 0552; Email: rohs@usc.edu

SUPPLEMENTAL FIGURES

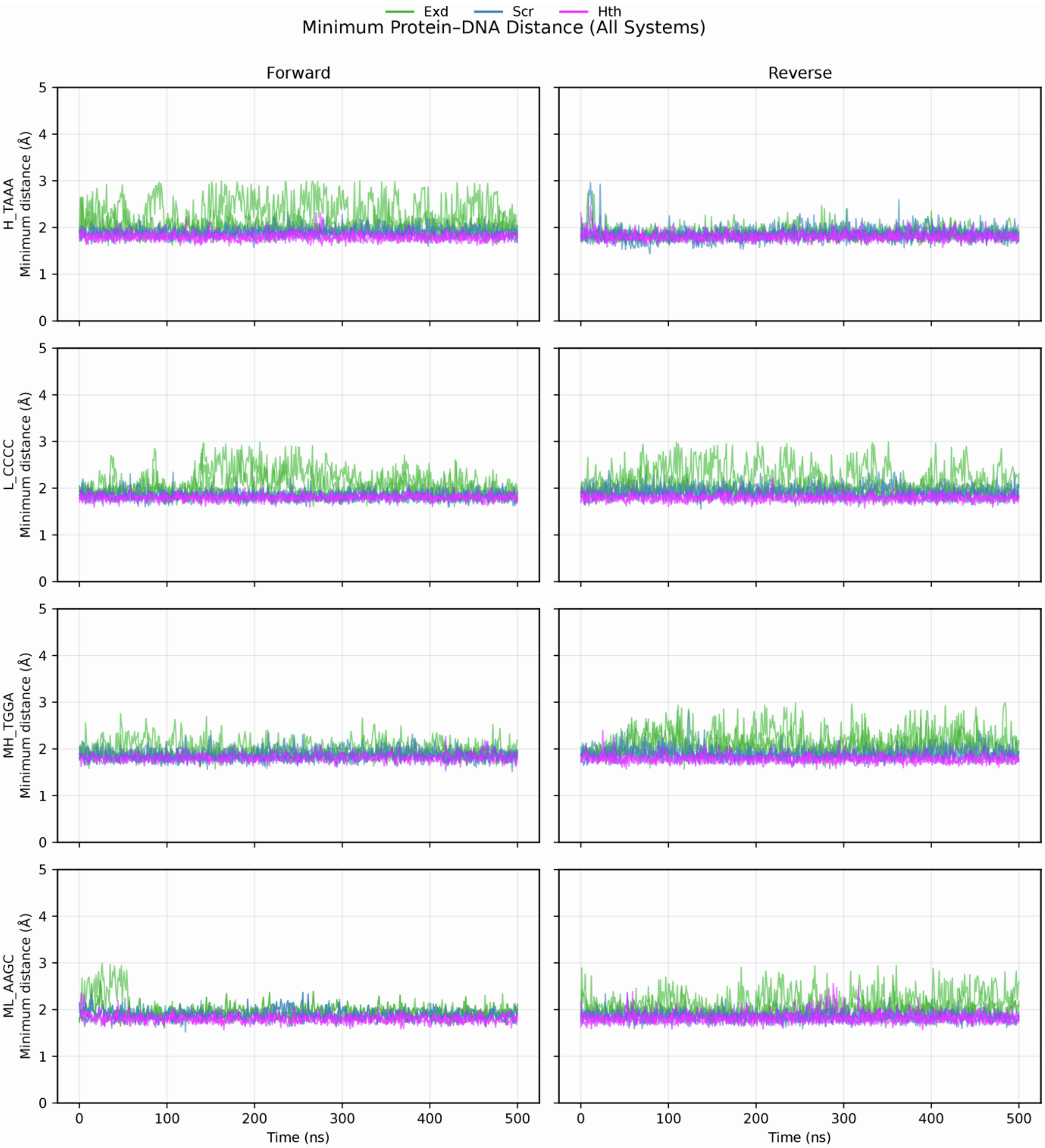

Fig. S1: Time-series traces show the minimum heavy-atom distance (Å) between the DNA and interface residues of Hth, Exd, and Scr across 500-ns simulations for each sequence variant in the forward (left) and reverse (right) orientations. Three replicas are overlaid for each system, with distances from the three proteins shown in green, blue, and magenta.

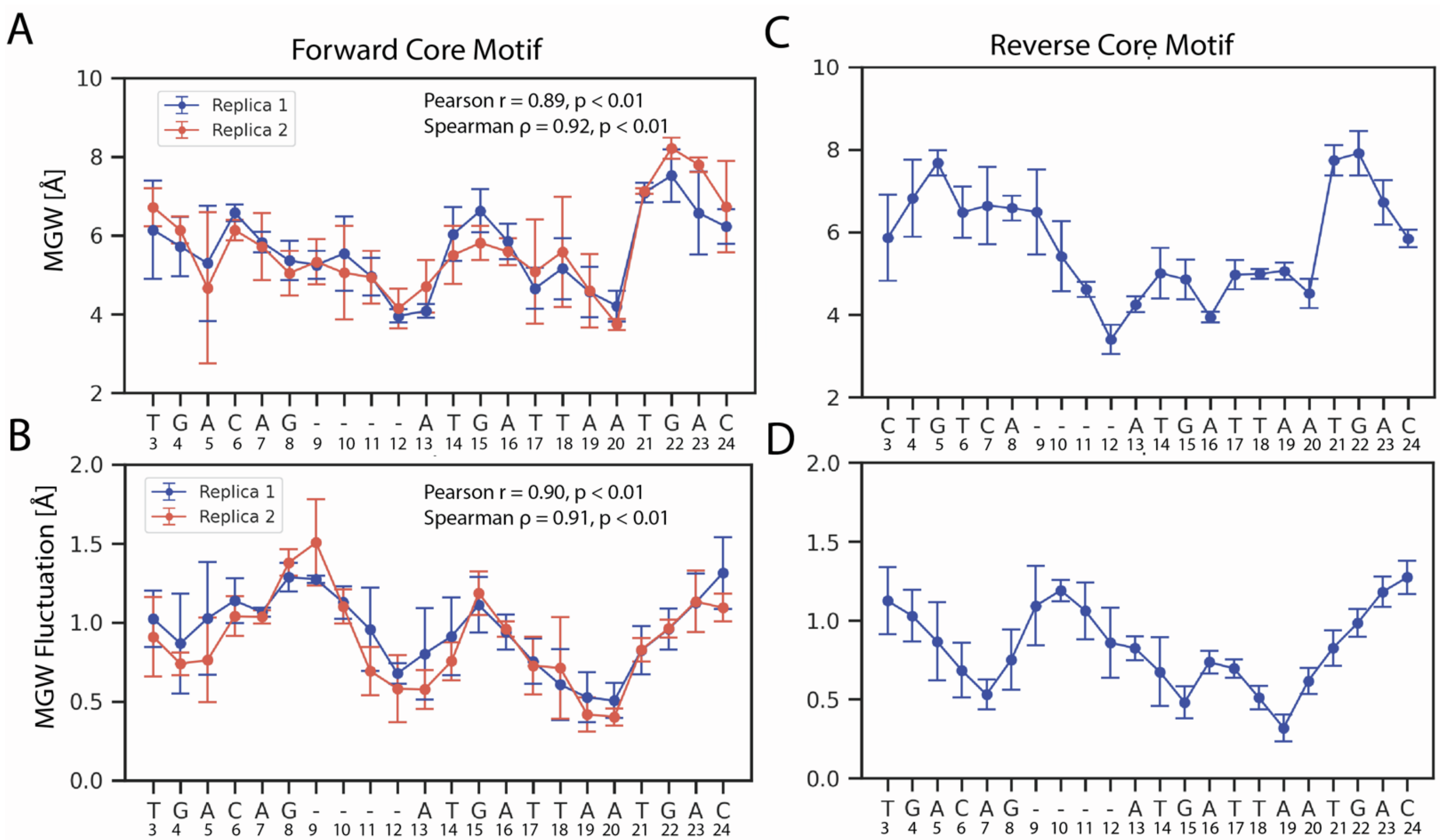

Fig. S2. Convergence of minor groove width (MGW) and MGW fluctuation (MGW-FL) across independently equilibrated MD replicas. (A) MGW profiles for the Forward core motif, comparing two independently equilibrated replica sets (blue, orange), each consisting of three 500-ns simulations; the last 200 ns of each simulation is shown. The two replica sets exhibit highly similar MGW patterns (Pearson $r = 0.89$, $p < 0.01$; Spearman's rank $\rho = 0.92$, $p < 0.01$). (B) Corresponding MGW-FL, computed as the pooled MGW standard deviation within each replica set, also shows strong agreement between replicas (Pearson $r = 0.90$, $p < 0.01$; Spearman's rank $\rho = 0.91$, $p < 0.01$). Error bars indicate variability among the three simulations within each replica set. (C) MGW profile for the high-affinity Reverse core motif. (D) Corresponding MGW-FL profile for system in (C). In panels (C–D), error bars represent variability among the three replicate simulations

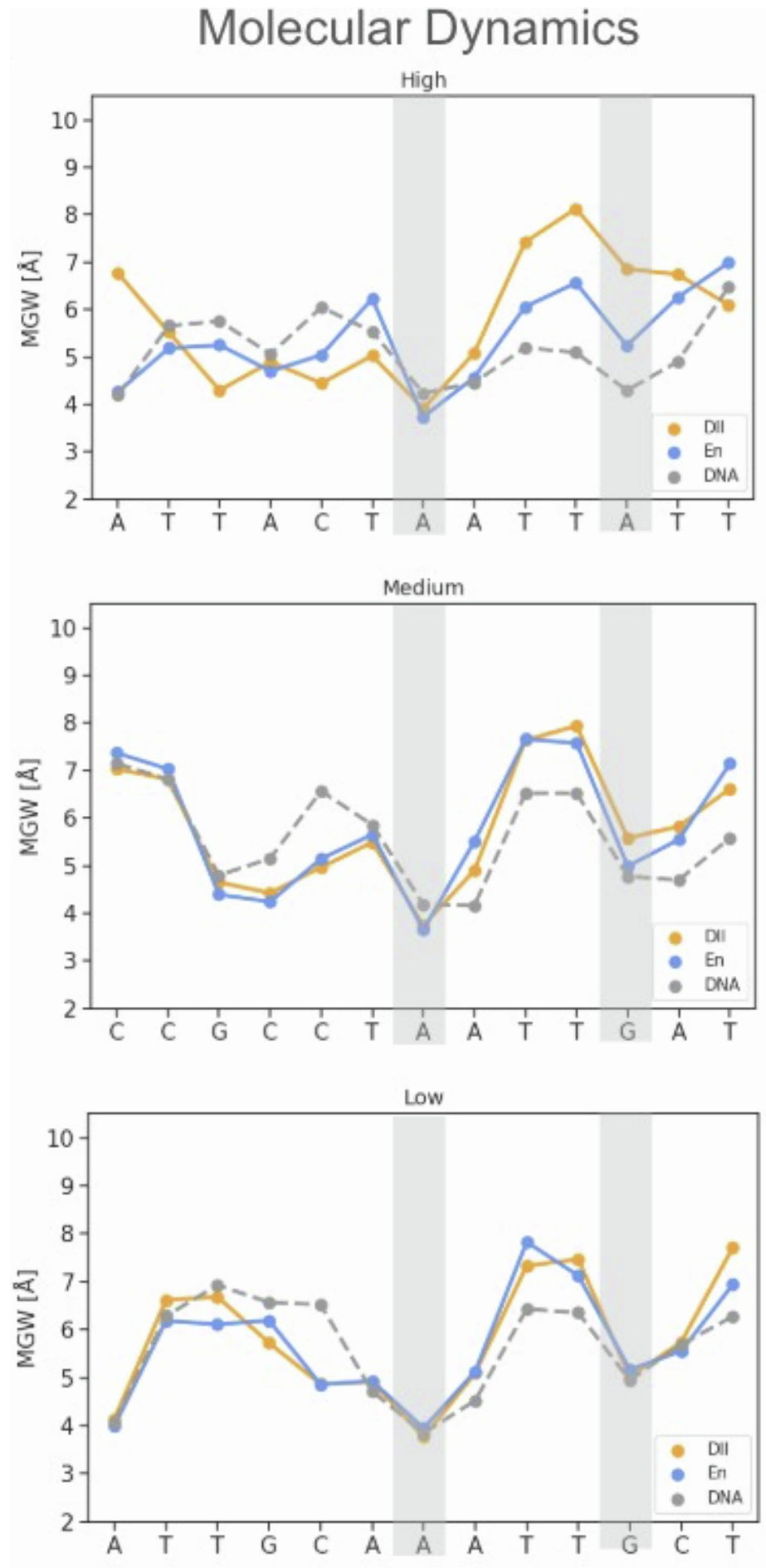

Fig. S3: DNA sequence profiles for various nucleotide sequences bound by Engrailed (En) and Distal-less (Dll) homeodomain proteins. Average DNA shape profiles calculated from MD simulations for three selected nucleotide sequences with relative binding affinities derived from SELEX-seq binding assays (High, Medium, and Low binding affinity). The DNA profile for each sequence bound to Dll (orange), En (blue), or unbound DNA (gray) is shown in each sequence

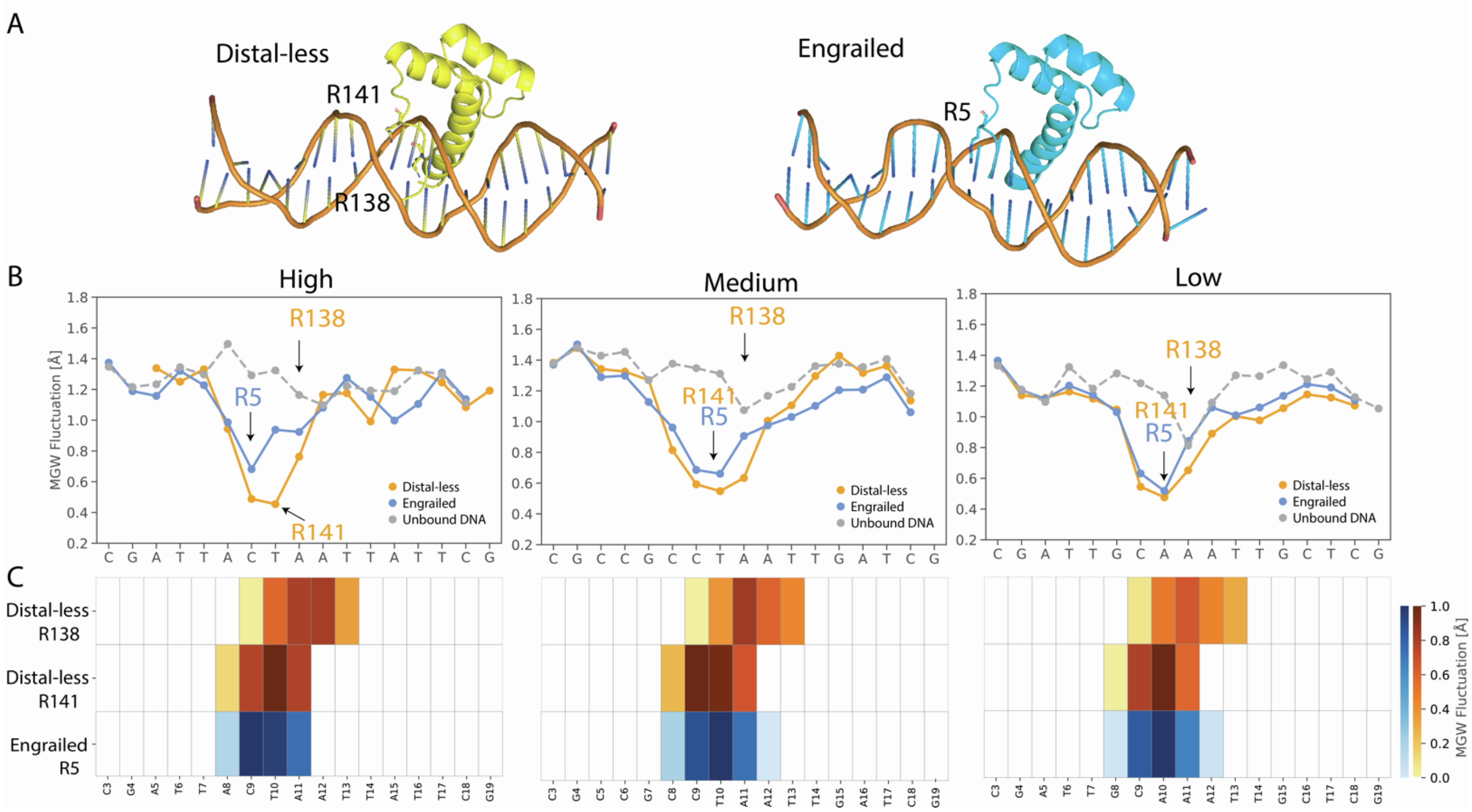

Fig. S4: Minor groove width fluctuations (MGW-FL) of various nucleotide sequences for Engrailed (En) and Distal-less (Dll) proteins. (A) Representative structures of the Dll and En protein–DNA complexes derived from MD simulations. Minor groove (MG)-interacting residues are labeled and shown as sticks. (B) Average MGW-FL profiles derived from MD simulations for three DNA sequences of different affinities (High, Medium, and Low affinity). For each sequence, MGW-FL is plotted for DNA bound to Dll (orange), En (blue), and unbound DNA (gray). (C) Residue–DNA contact maps for MG-interacting residues identified in (A), showing the closest DNA positions used to determine the residue labels in (B) (see Materials and Methods for details).

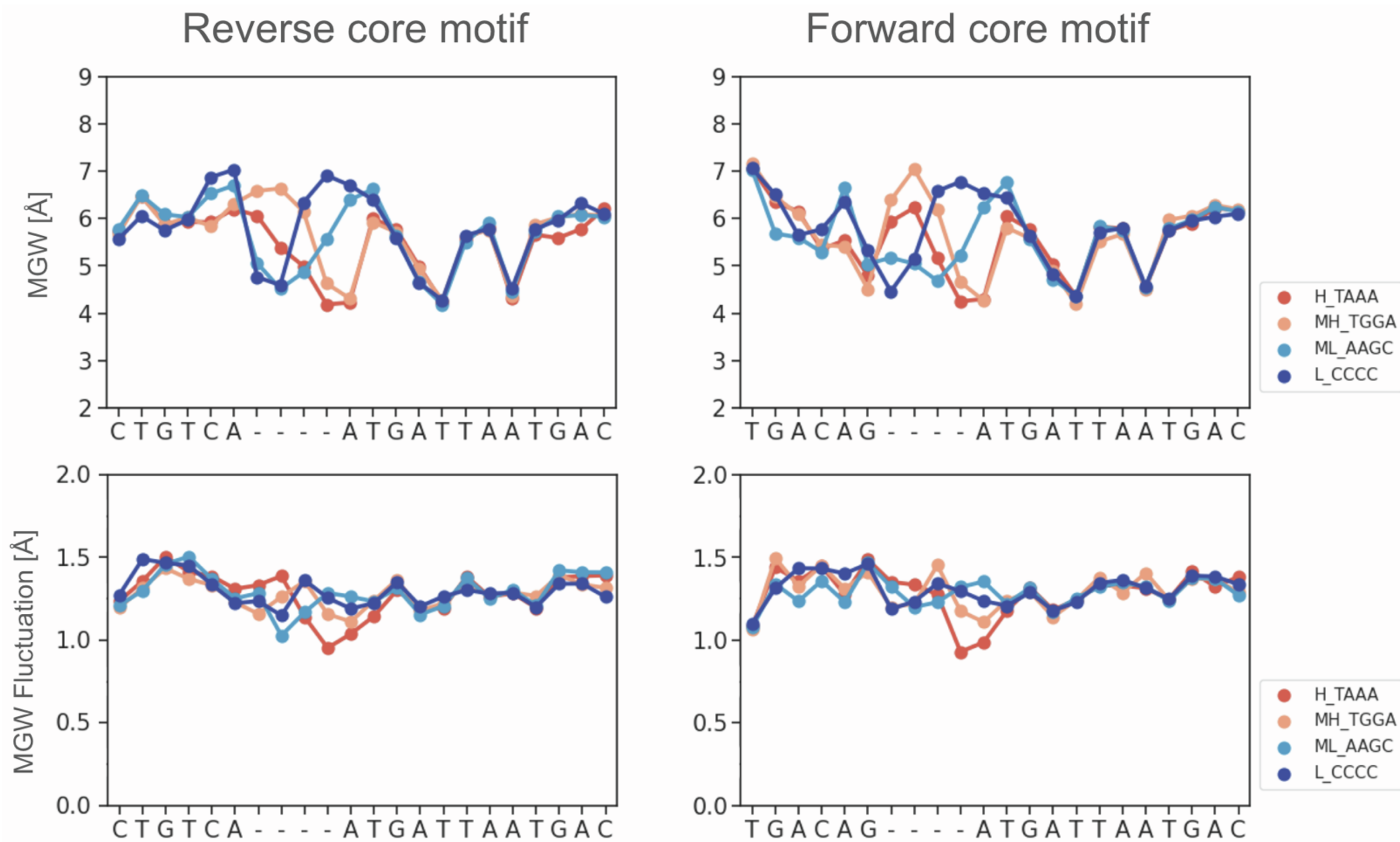

Fig. S5: Average unbound MGW and MGW-FL for four DNA sequences of various affinities for sequences with reverse core motif and sequences with forward core motif from MD simulations.

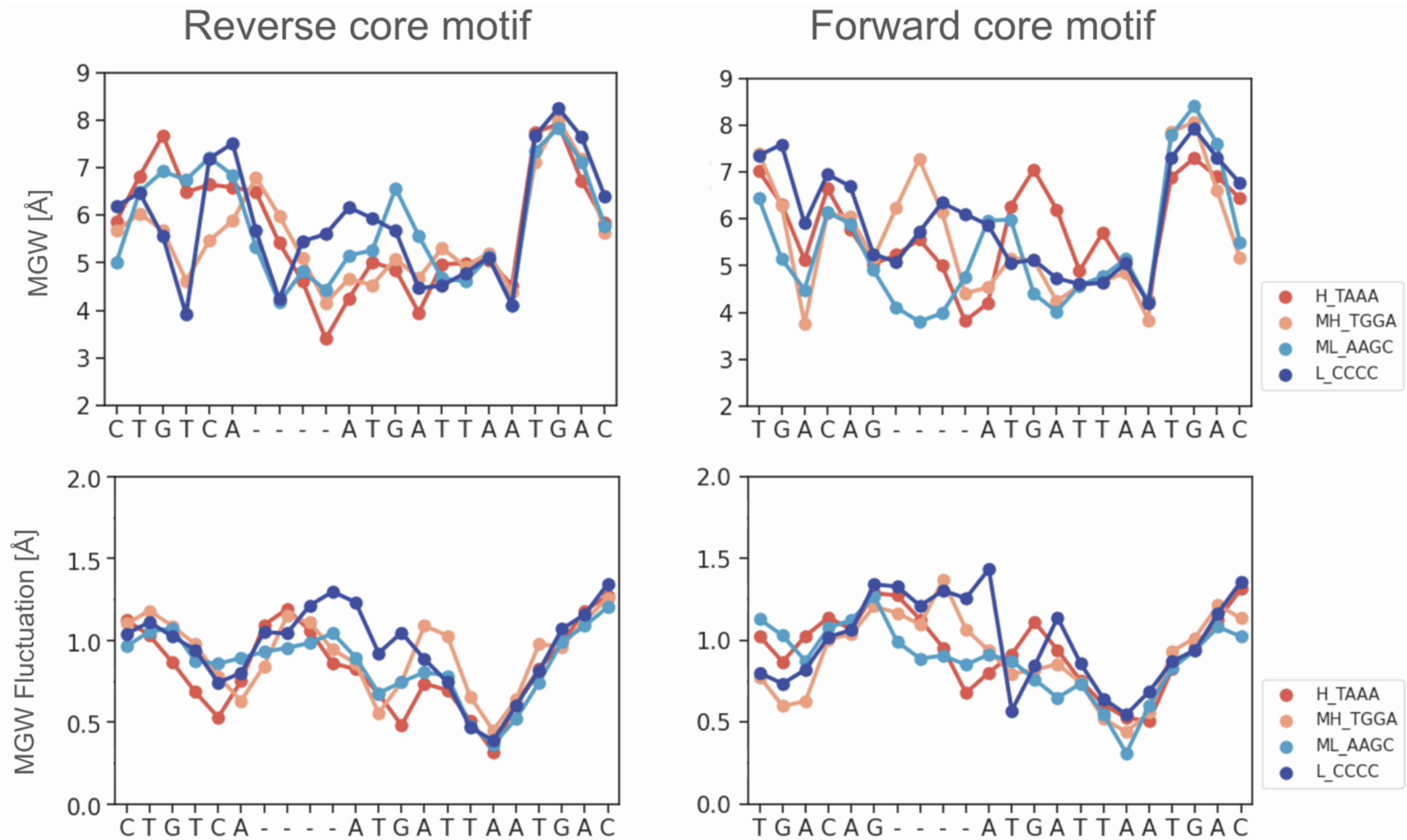

Fig. S6: Average MGW and MGW-FL for bound DNA fragments of various affinities calculated from MD simulations for sequences with reverse core motif and sequences with forward core motif.

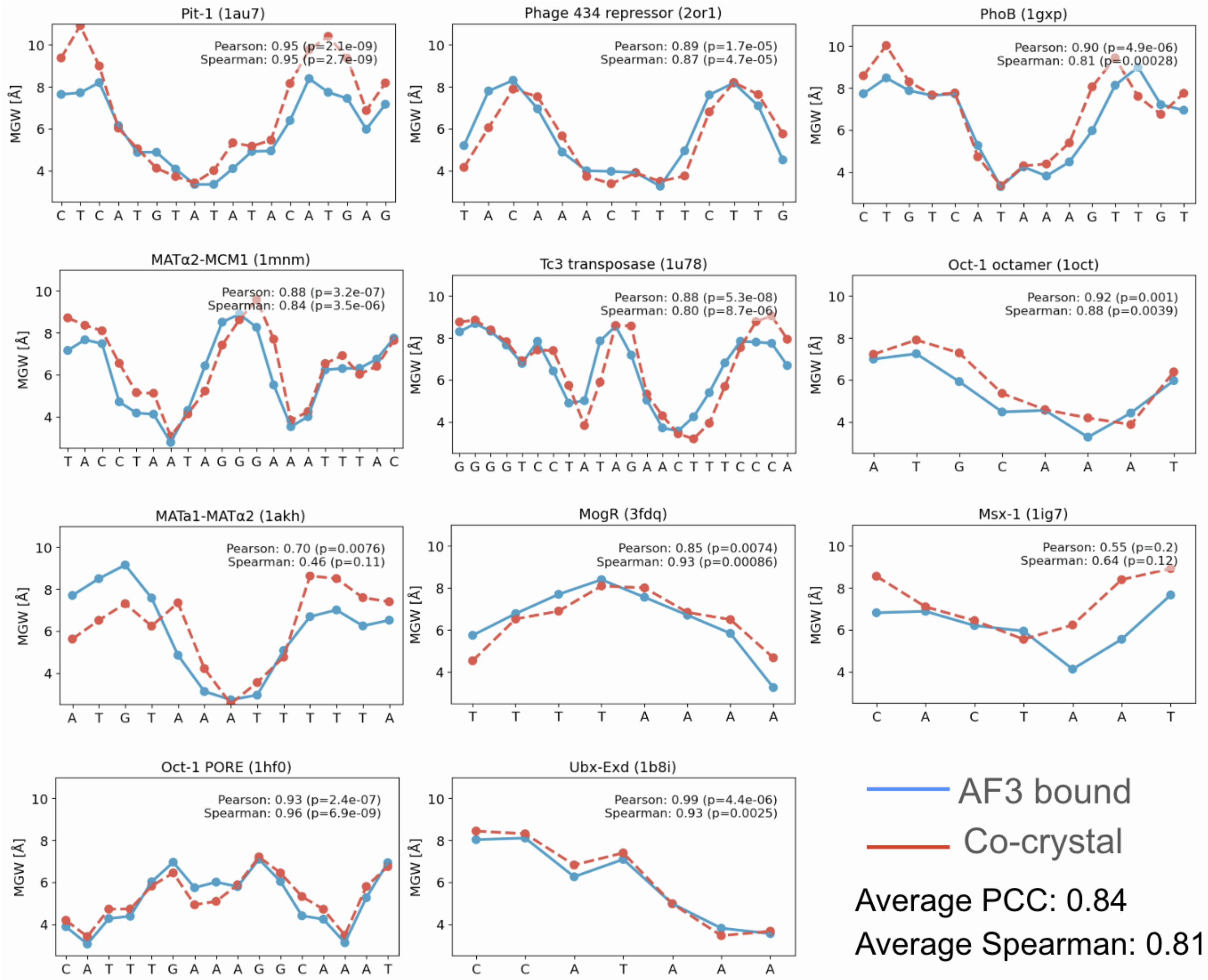

Fig. S7: MGW comparison for complexes predicted with AlphaFold 3 (AF3) (2) (blue) and co-crystal structures (red) for eleven selected protein–DNA complexes obtained from (3).

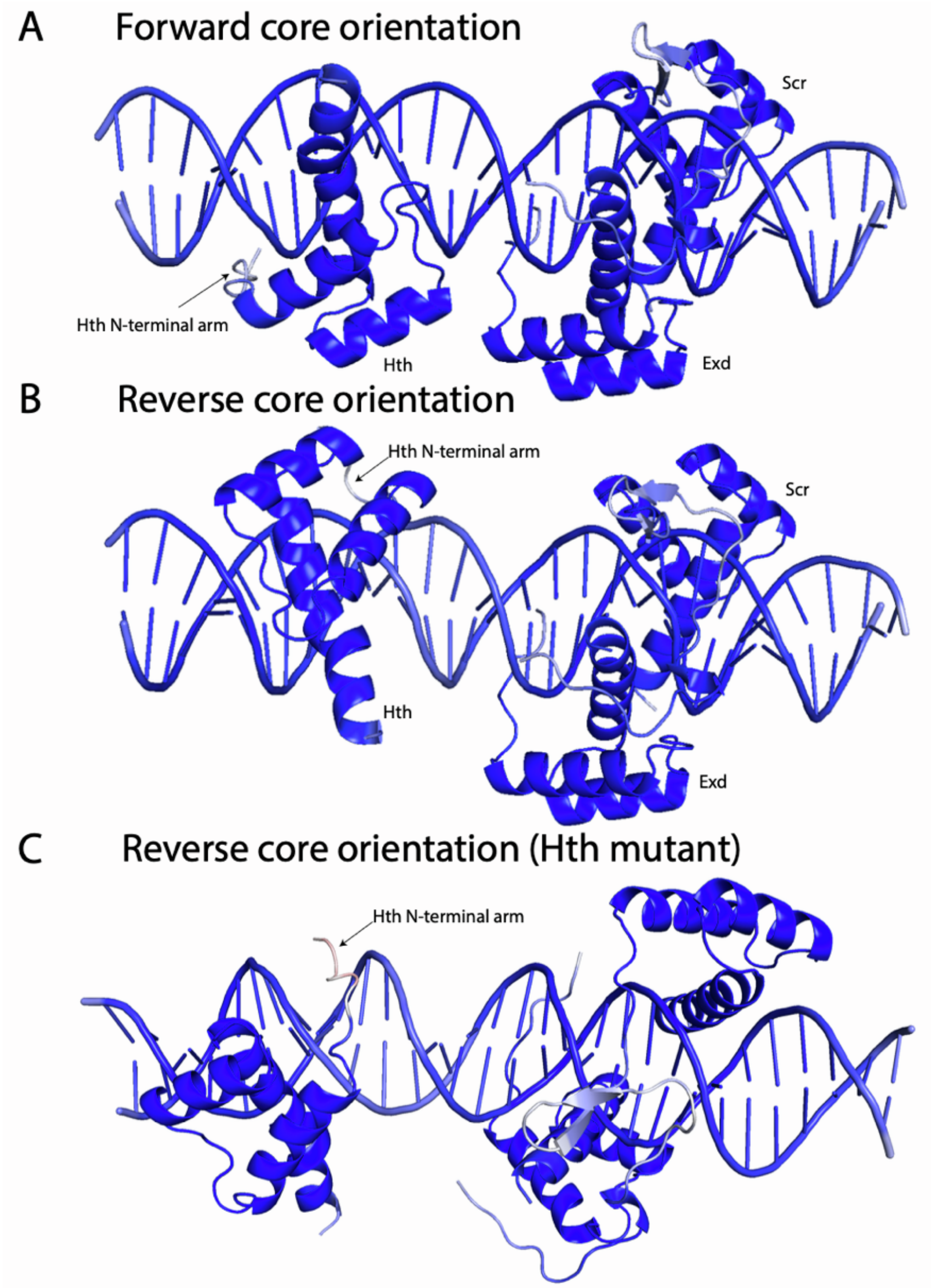

Fig. S8: AF3 prediction for Hth complex with forward (A), reverse core orientations (B), and Hth mutant (C). The structures are colored based on AF3 pLDDT scores (blue color implying higher AF3 confidence within the standard 0 to 100 range). Arrow indicates the mutated N-terminal arm residues with reduced pLDDT score.

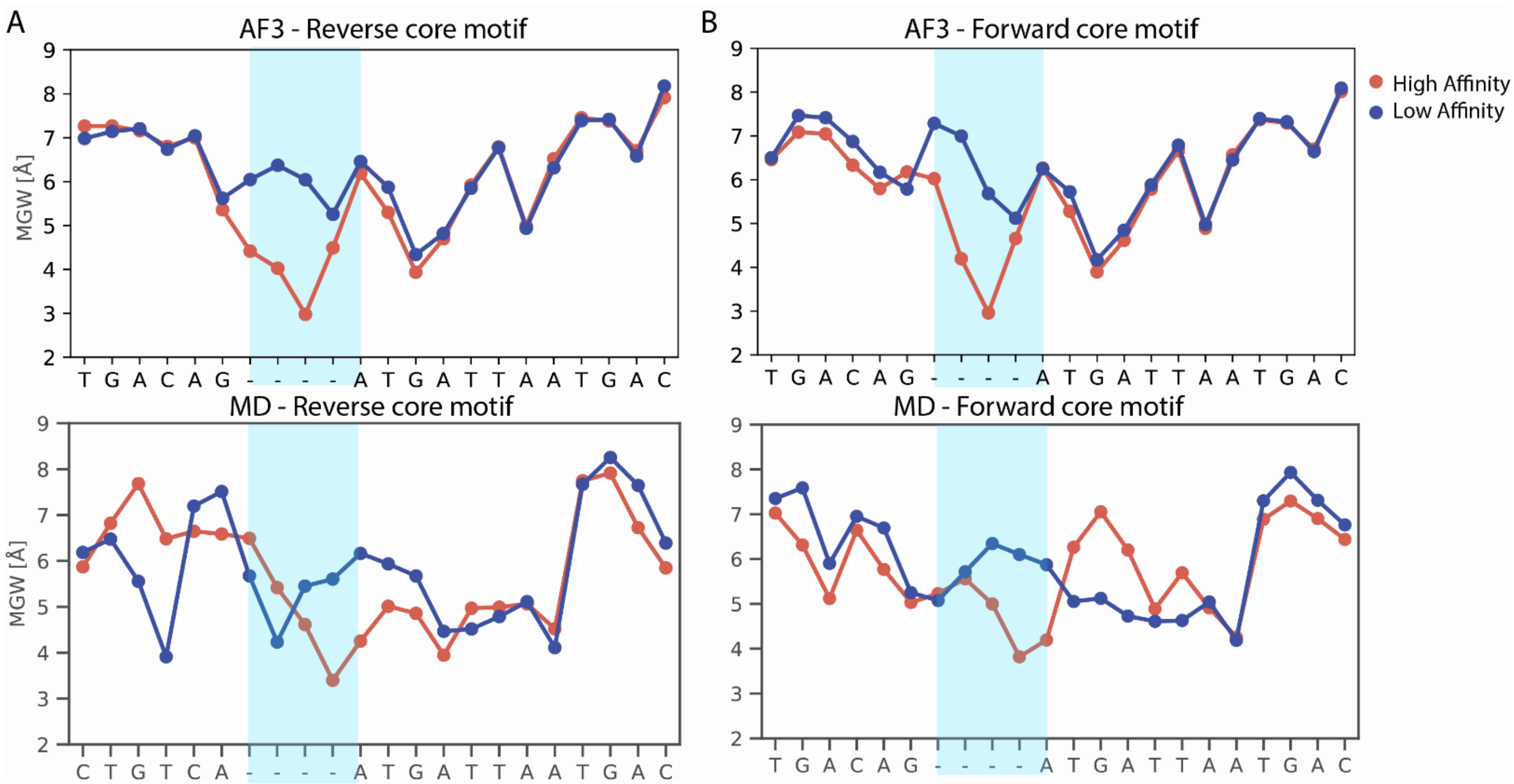

Fig. S9: MGW comparison between AF3-derived predictions (top panel) and MD simulations (bottom panel) for high-affinity and low-affinity DNA sequences in both (A) forward and (B) reverse core motif orientations.

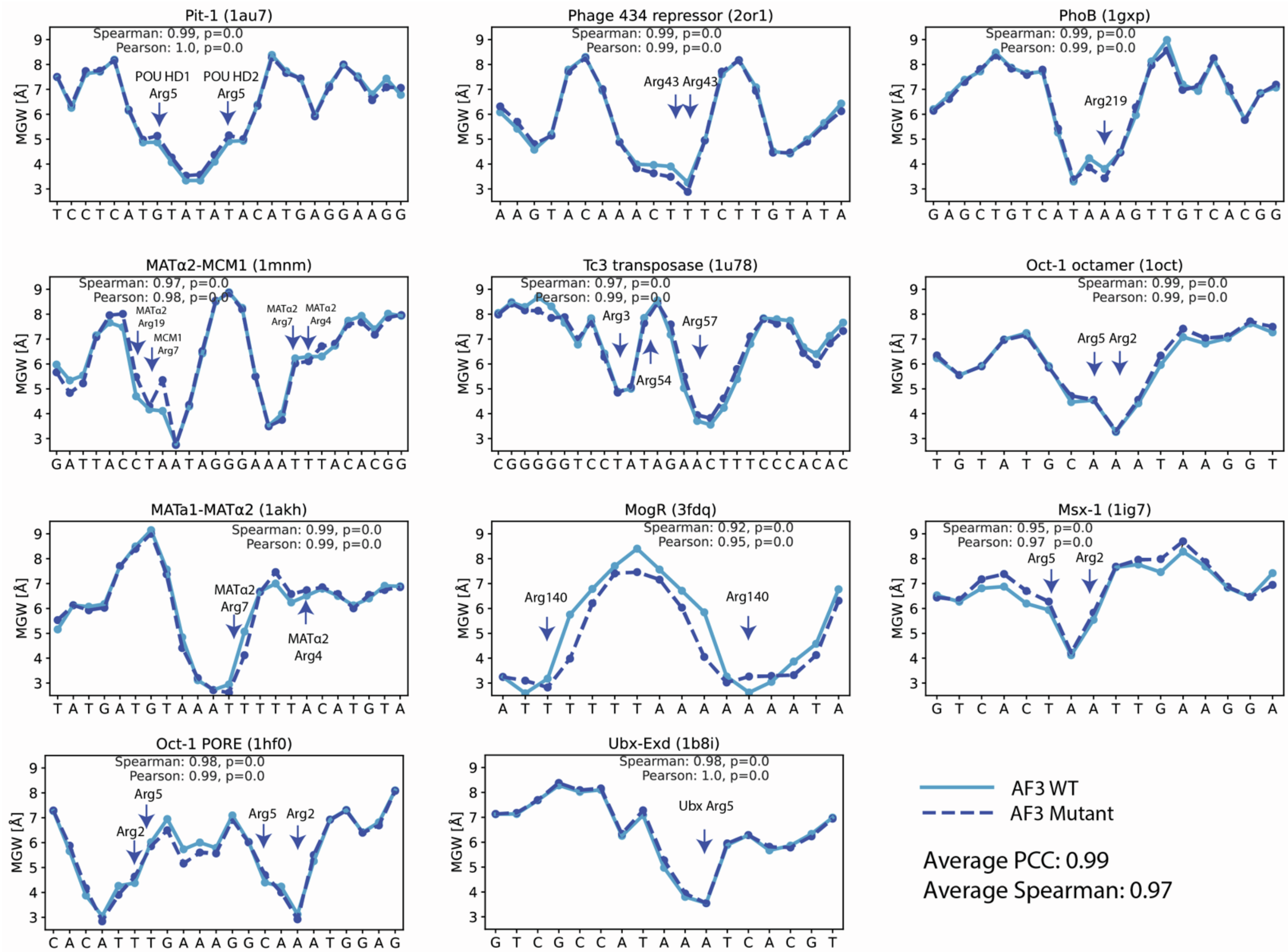

Fig. S10: MGW comparison for AF3-predicted wild-type (WT) (light blue solid line) and AF3-predicted mutant (darker blue dashed line) structures for eleven selected protein–DNA complexes obtained from (3). The mutated residues are annotated with arrows.

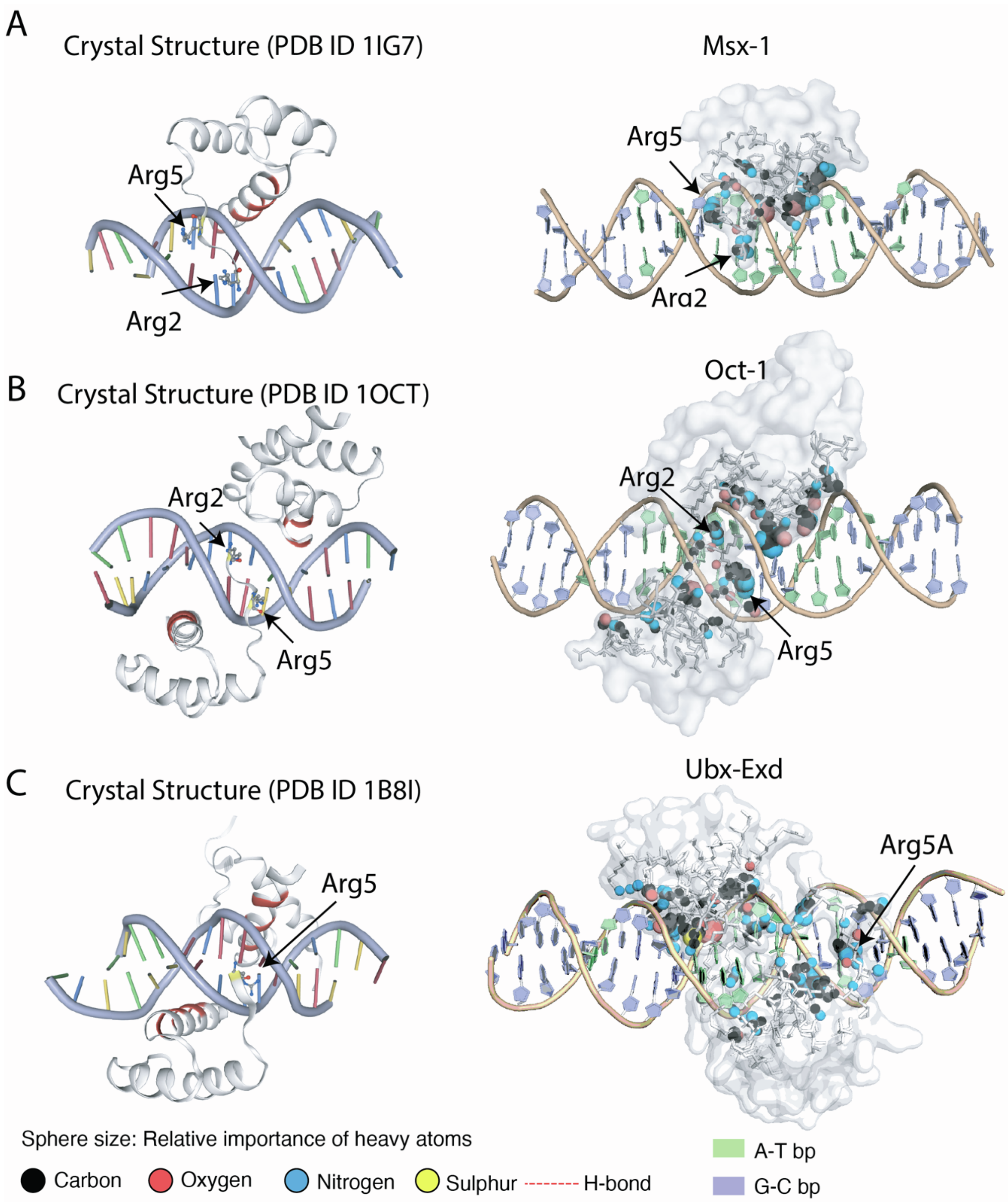

Fig. S11: DeepPBS (4) heavy-atom relative importance (RI) scores for various AF3-predicted wild-type (WT) homeodomain–DNA complexes. Sphere sizes reflect the relative importance of each heavy atom. Key minor groove (MG) contacts are annotated for these protein–DNA complexes. Visualizations of the co-crystal structures are shown on the left, and DeepPBS RI scores are shown on the right.

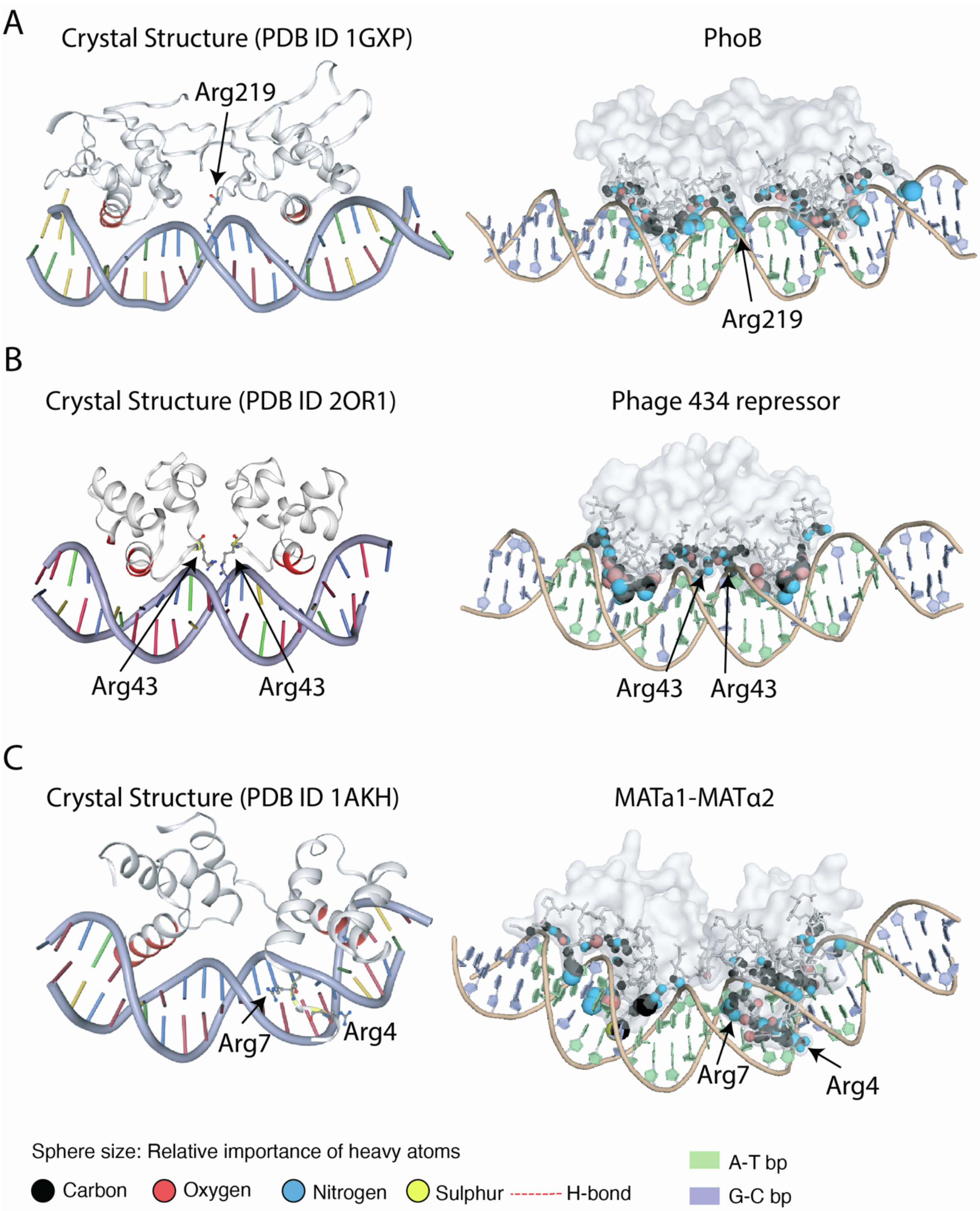

Fig. S12: DeepPBS (4) heavy-atom relative importance (RI) scores for various AF3-predicted wild-type (WT) protein–DNA complexes. Sphere sizes reflect the relative importance of each heavy atom. Key minor groove (MG) contacts are annotated for these protein–DNA complexes. Visualizations of the co-crystal structures are shown on the left, and DeepPBS RI scores are shown on the right.

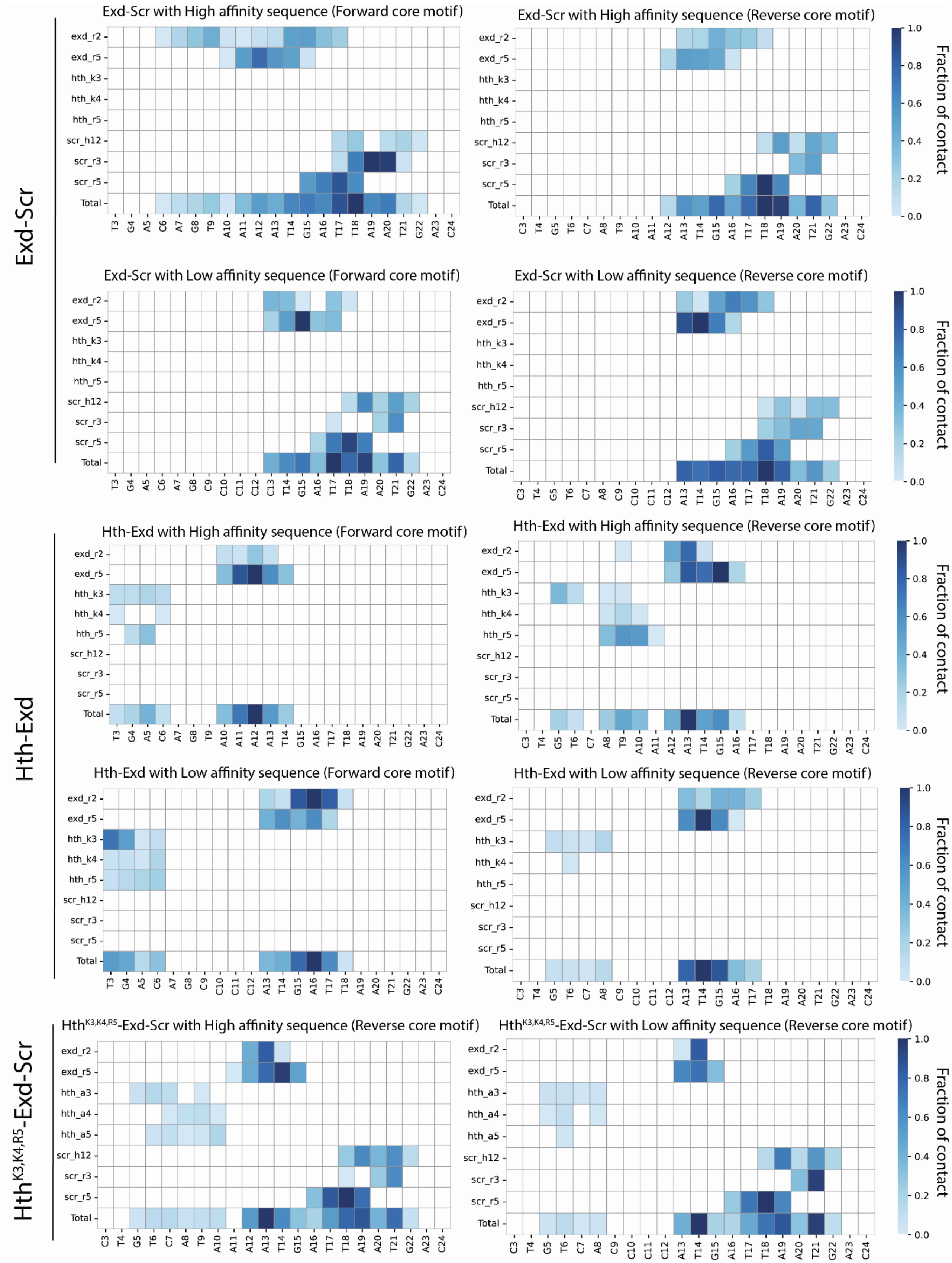

Fig. S13: Minor groove contacts for high- and low-affinity sequences across various protein–DNA complex architectures. The complexes analyzed include the Hth-Exd–DNA complex, the Exd-Scr–DNA complex, and the mutant Hth^{K3A,K4A,R5A}-Exd–DNA complex. DNA sequences contain both forward and reverse core motifs.

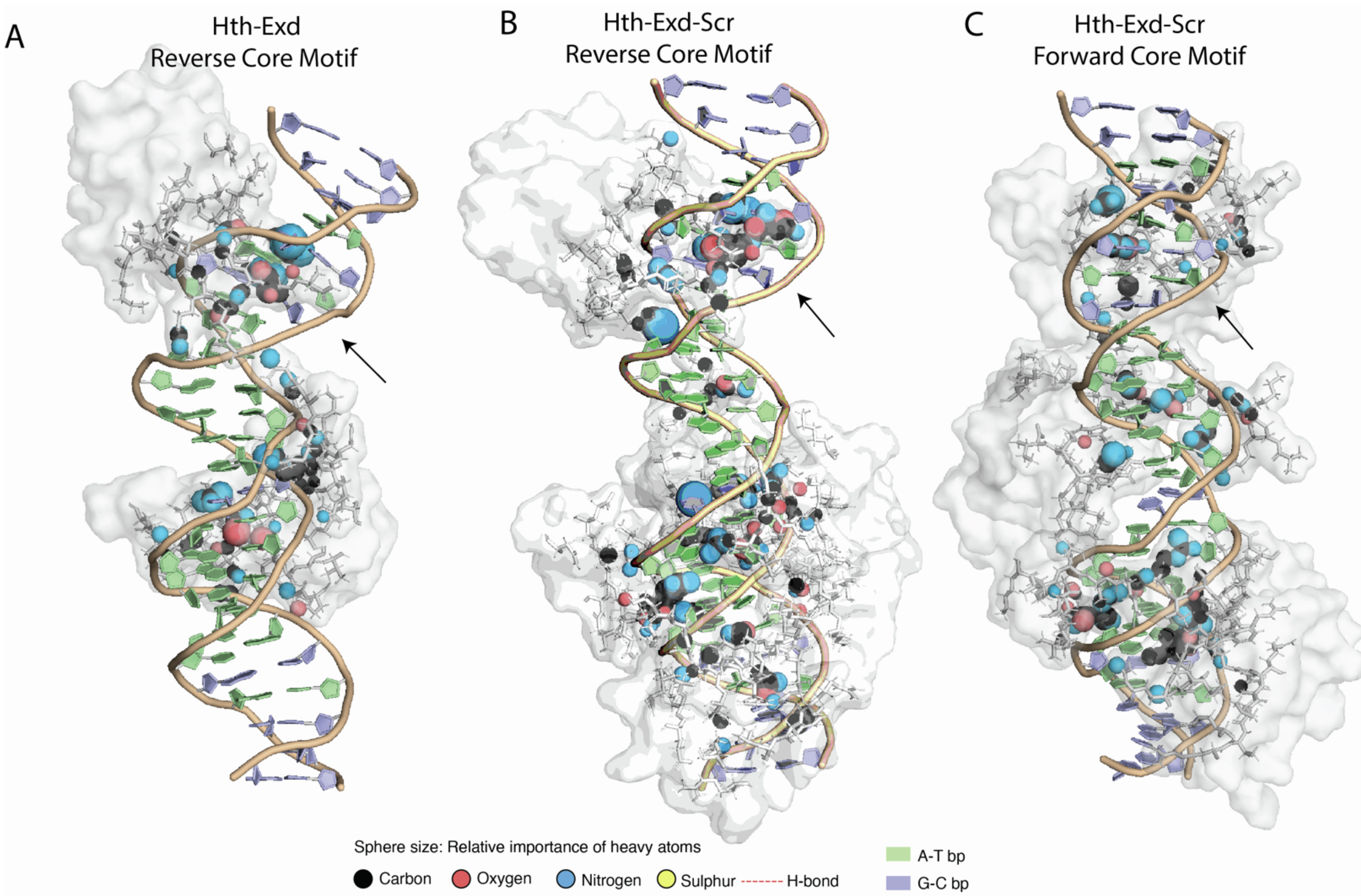

Fig. S14: DeepPBS relative importance (RI) analysis of Hth-Exd-Scr and Hth-Exd complexes. Sphere sizes represent the RI scores of heavy atoms in mediating protein–DNA interactions, with a black arrow indicating the Hth core motif region. (A) Hth-Exd complex with DNA in the reverse core motif orientation, (B) Hth-Exd-Scr complex with DNA in the reverse orientation, and (C) Hth-Exd-Scr complex with DNA in the forward orientation.

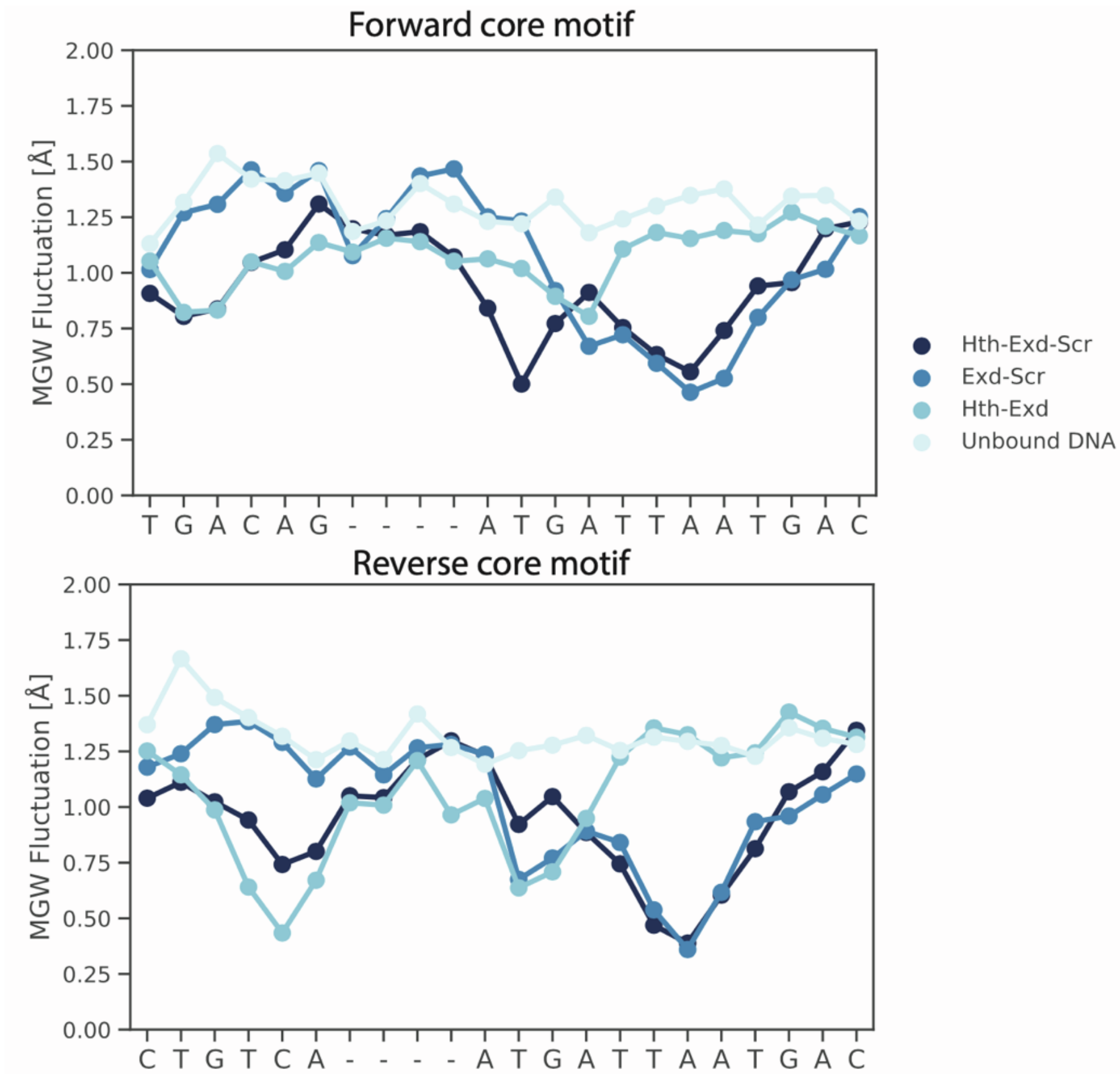

Fig. S15: MGW-FL of Hth-Exd-Scr, Hth-Exd, and Exd-Scr complexes bound to low-affinity nucleotide sequences with core motifs in (A) forward versus (B) reverse core orientations. Unbound DNA is also shown.

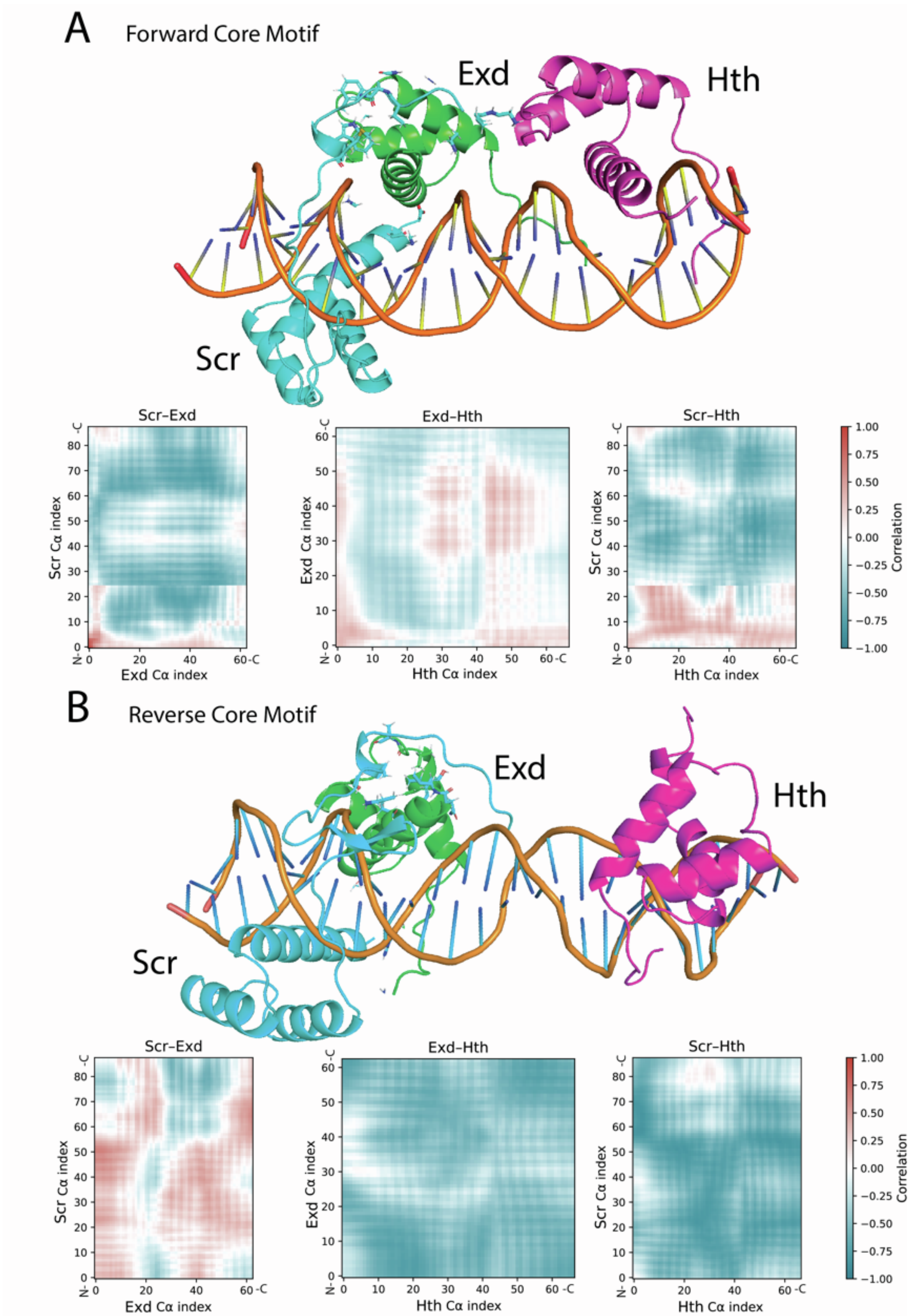

Fig. S16: Replica-averaged dynamic cross-correlation matrices (DCCMs) for the Hth-Exd-Scr trimer in the (A) forward and (B) reverse orientations. Positive values indicate coordinated motion, and negative values indicate anticorrelated motion. The patterns reveal orientation-dependent coupling between Scr-Exd, Exd-Hth, and Scr-Hth that reflects both direct contacts and DNA-mediated correlation within the trimer.

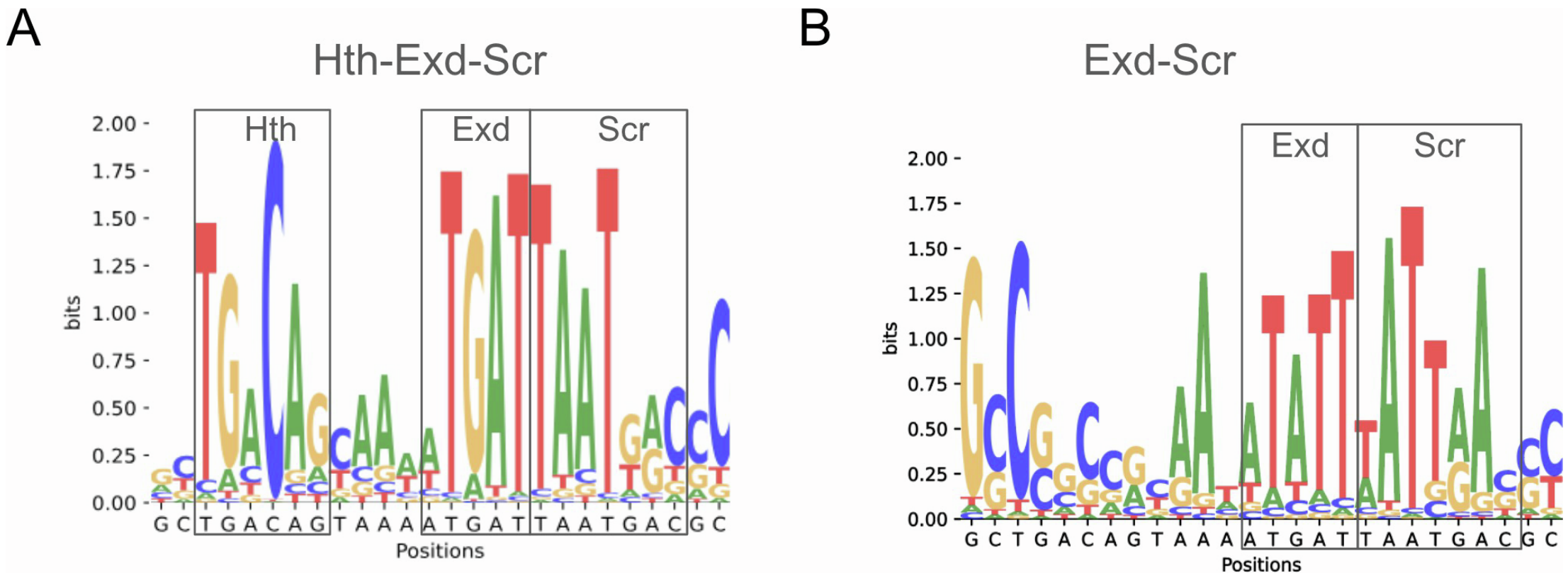

Fig. S17: DNA binding specificity predicted by DeepPBS for the Hth-Exd-Scr trimer with high- and low-affinity sequences derived from SELEX-seq experiments (Table S1) (5).

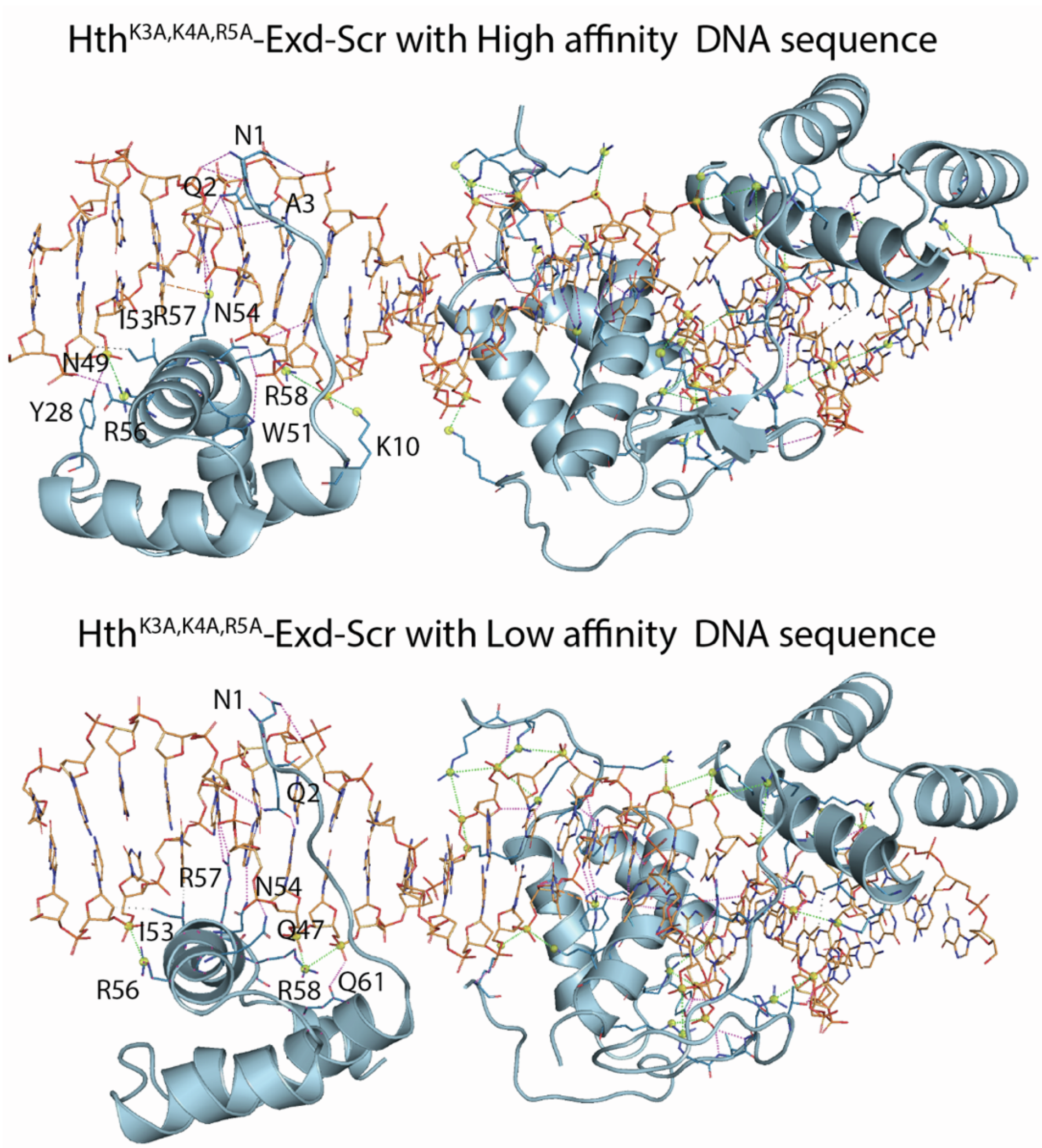

Fig. S18: DNA interactions of the Hth-Exd-Scr trimer with high- and low-affinity nucleotide sequences. Magenta lines indicate hydrogen bonding, orange dashes indicate π-cation interaction, gray indicate hydrophobic interaction, and green dashes indicate salt bridges.

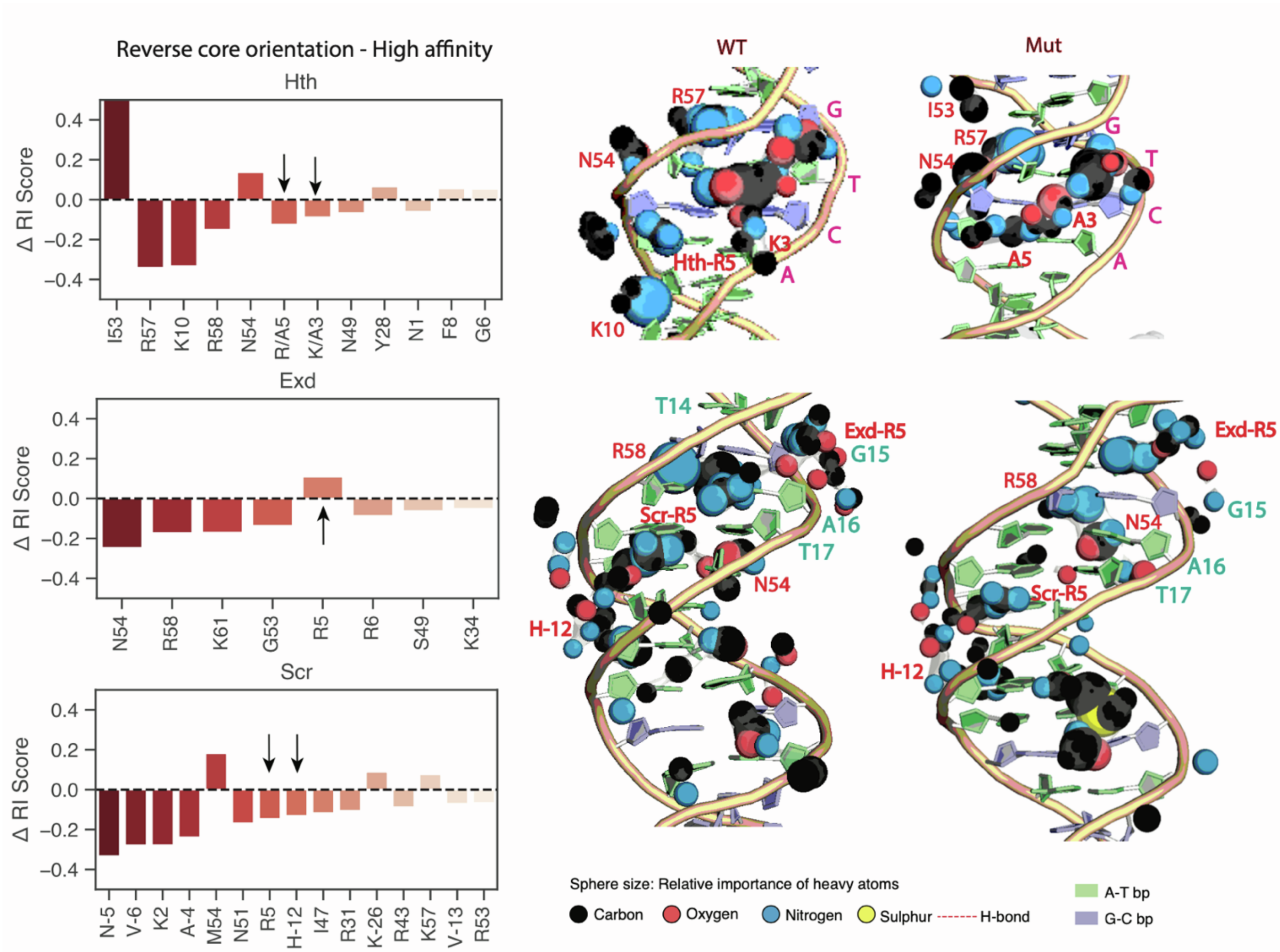

Fig. S19: Change in relative importance (RI) scores for the Hth-Exd-Scr–DNA ternary complex with high-affinity nucleotide sequence upon mutation of Hth residues. RI changes are computed as follows: $\Delta RI = RI_{\text{Mutant}} - RI_{\text{WT}}$. Arrows highlight residues with MG contacts of each protein. The right panel shows visualizations of DeepPBS-predicted RI scores for wild-type (WT) and mutant (Mut) complexes, with key residues labeled. Protein omitted for visibility.

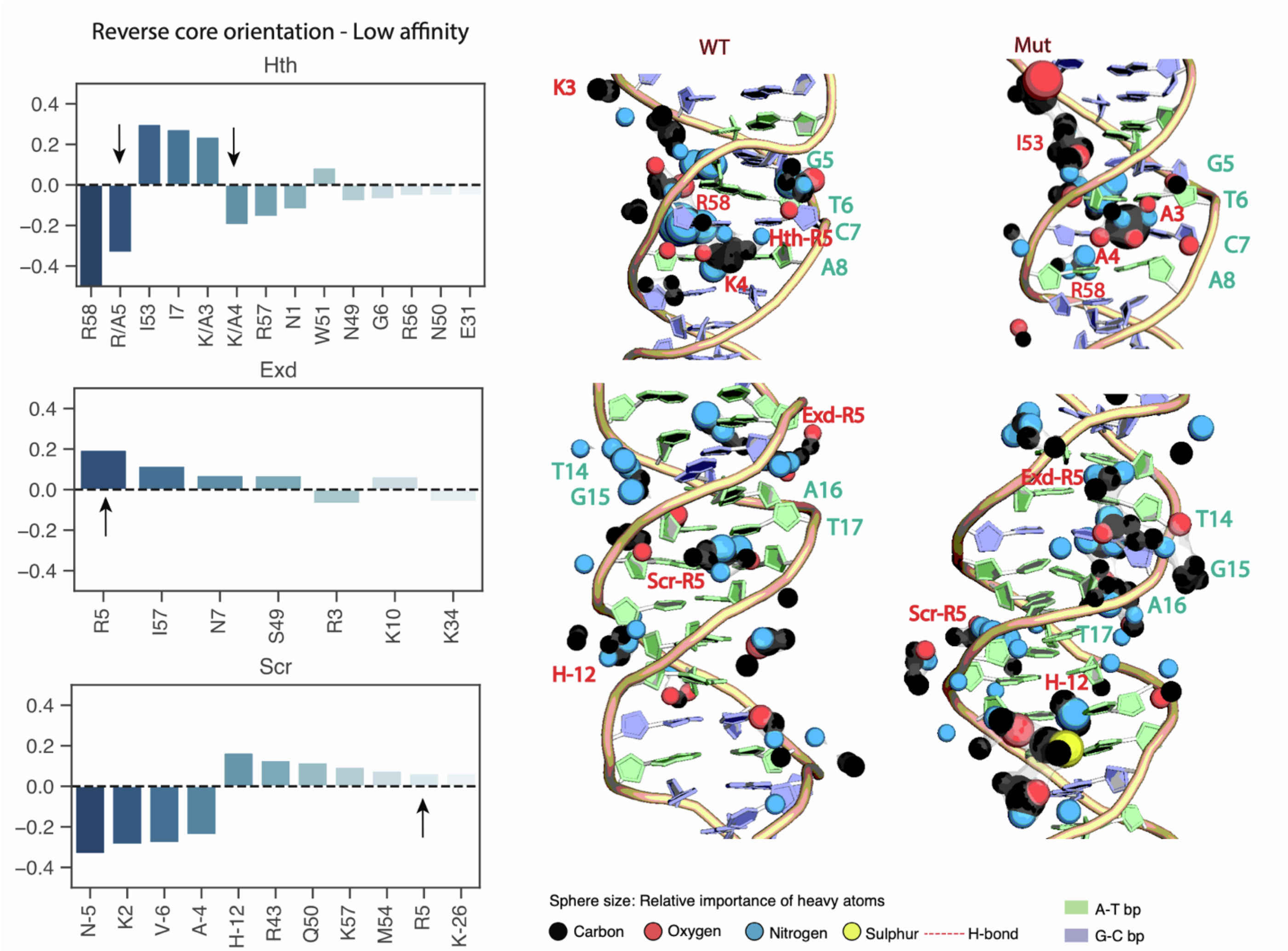

Fig. S20: Change in relative importance (RI) scores for the Hth-Exd-Scr–DNA complex with low-affinity sequence upon mutation of specific Hth residues. RI changes are computed as follows: $\Delta RI = RI_{\text{Mutant}} - RI_{\text{WT}}$. Arrows highlight residues that interact with the minor groove. The right panel shows visualizations of DeepPBS-derived RI scores for wild-type (WT) and mutant (Mut) complexes, with key residues labeled. Protein omitted for visibility.

SUPPLEMENTAL TABLES

Affinity	Sequence
High - Forward	$C_{1}G_{2}T_{3}G_{4}A_{5}C_{6}A_{7}G_{8}\mathbf{T_{9}A_{10}A_{11}A_{12}}A_{13}T_{14}G_{15}A_{16}T_{17}T_{18}A_{19}A_{20}T_{21}G_{22}A_{23}C_{24}C_{25}G_{26}$
Medium High -Forward	$C_{1}G_{2}T_{3}G_{4}A_{5}C_{6}A_{7}G_{8}\mathbf{T_{9}G_{10}G_{11}A_{12}}A_{13}T_{14}G_{15}A_{16}T_{17}T_{18}A_{19}A_{20}T_{21}G_{22}A_{23}C_{24}C_{25}G_{26}$
Medium Low -Forward	$C_{1}G_{2}T_{3}G_{4}A_{5}C_{6}A_{7}G_{8}\mathbf{T_{9}A_{10}A_{11}G_{12}C}_{13}T_{14}G_{15}A_{16}T_{17}T_{18}A_{19}A_{20}T_{21}G_{22}A_{23}C_{24}C_{25}G_{26}$
Low - Forward	$C_{1}G_{2}T_{3}G_{4}A_{5}C_{6}A_{7}G_{8}\mathbf{C_{9}C_{10}C_{11}C_{12}}A_{13}T_{14}G_{15}A_{16}T_{17}T_{18}A_{19}A_{20}T_{21}G_{22}A_{23}C_{24}C_{25}G_{26}$
High - Reverse	$C_{1}G_{2}C_{3}T_{4}G_{5}T_{6}C_{7}A_{8}\mathbf{T_{9}A_{10}A_{11}A_{12}}A_{13}T_{14}G_{15}A_{16}T_{17}T_{18}A_{19}A_{20}T_{21}G_{22}A_{23}C_{24}C_{25}G_{26}$
Medium High -Reverse	$C_{1}G_{2}C_{3}T_{4}G_{5}T_{6}C_{7}A_{8}\mathbf{T_{9}G_{10}G_{11}A_{12}}A_{13}T_{14}G_{15}A_{16}T_{17}T_{18}A_{19}A_{20}T_{21}G_{22}A_{23}C_{24}C_{25}G_{26}$
Medium Low -Reverse	$C_{1}G_{2}C_{3}T_{4}G_{5}T_{6}C_{7}A_{8}\mathbf{T_{9}A_{10}A_{11}G_{12}C}_{13}T_{14}G_{15}A_{16}T_{17}T_{18}A_{19}A_{20}T_{21}G_{22}A_{23}C_{24}C_{25}G_{26}$
Low - Reverse	$C_{1}G_{2}C_{3}T_{4}G_{5}T_{6}C_{7}A_{8}\mathbf{C_{9}C_{10}C_{11}C_{12}}A_{13}T_{14}G_{15}A_{16}T_{17}T_{18}A_{19}A_{20}T_{21}G_{22}A_{23}C_{24}C_{25}G_{26}$

Table S1: Nucleotide sequences used for MD simulation of the tertiary Hth-Exd-Scr–DNA complex. Spacer sequences derived from SELEX-seq experiments using the Top-Down Crawl (1) method are shown in bold.

Scr<br>88 *aa*	GKKNPPQIYPWMKRVHLGTSTVNANGETKRQRTSYTRYQTLELEKEFHFNRYLTRRRRIEIAH<br>ALCLTERQIKIWFQNRRMKWKKEHK
Exd<br>63 *aa*	ARRKRRNFSKQASEILNEYFYSHLSNPYPSEEAKEELARKCGITVSQVSNWFGNKRIRYKKNI
Hth<br>67 *aa*	NQKKRGIFPKVATNILRAWLFQHLTHPYPSEDQKKQLAQDTGLTILQVNNWFINARRRIVQPMIDQS
Hth<br>mutant<br>67 *aa*	NQAAAGIFPKVATNILRAWLFQHLTHPYPSEDQKKQLAQDTGLTILQVNNWFINARRRIVQPMIDQS

Table S2: Protein sequences used for MD simulation of the tertiary Hth-Exd-Scr–DNA complex. The length of each protein is annotated below in number of amino acids (aa). The mutated residues in the Hth^{K3A,K4A,R5} mutant is annotated with underline. Select N-terminal arm residues such as Scr H-12, Scr R3, Scr R5, Exd R2, Exd R5, Hth K3, Hth K4, and Hth R5 are highlighted in red.

ID	Type	Orient	DNA	Complex	Mut	Reps	Time (ns)
1	Bound	Forward	H_TAAA	Hth–Exd–Scr (trimer)	WT	3	500
2	Bound	Forward	MH_TGGA	Hth–Exd–Scr (trimer)	WT	3	500
3	Bound	Forward	ML_AAGC	Hth–Exd–Scr (trimer)	WT	3	500
4	Bound	Forward	L_CCCC	Hth–Exd–Scr (trimer)	WT	3	500
5	Bound	Forward	H_TAAA	Hth–Exd (dimer)	WT	3	500
6	Bound	Forward	L_CCCC	Hth–Exd (dimer)	WT	3	500
7	Bound	Forward	H_TAAA	Exd–Scr (dimer)	WT	3	500
8	Bound	Forward	L_CCCC	Exd–Scr (dimer)	WT	3	500
9	Bound	Reverse	H_TAAA	Hth–Exd–Scr (trimer)	WT	3	500
10	Bound	Reverse	MH_TGGA	Hth–Exd–Scr (trimer)	WT	3	500
11	Bound	Reverse	ML_AAGC	Hth–Exd–Scr (trimer)	WT	3	500
12	Bound	Reverse	L_CCCC	Hth–Exd–Scr (trimer)	WT	3	500
13	Bound	Reverse	H_TAAA	Hth–Exd (dimer)	WT	3	500
14	Bound	Reverse	L_CCCC	Hth–Exd (dimer)	WT	3	500
15	Bound	Reverse	H_TAAA	Exd–Scr (dimer)	WT	3	500
16	Bound	Reverse	L_CCCC	Exd–Scr (dimer)	WT	3	500
17	Bound	Reverse	H_TAAA	Hth–Exd–Scr (trimer)	Mutant	3	500
18	Bound	Reverse	L_CCCC	Hth–Exd–Scr (trimer)	Mutant	3	500
19	Bound	Reverse	H_TAAA	Hth–Exd (dimer)	Mutant	3	500
20	Bound	Reverse	L_CCCC	Hth–Exd (dimer)	Mutant	3	500
21	Unbound	Forward	H_TAAA	DNA only	WT	3	300
22	Unbound	Forward	MH_TGGA	DNA only	WT	3	300
23	Unbound	Forward	ML_AAGC	DNA only	WT	3	300
24	Unbound	Forward	L_CCCC	DNA only	WT	3	300
25	Unbound	Reverse	H_TAAA	DNA only	WT	3	300
26	Unbound	Reverse	MH_TGGA	DNA only	WT	3	300
27	Unbound	Reverse	ML_AAGC	DNA only	WT	3	300
28	Unbound	Reverse	L_CCCC	DNA only	WT	3	300
29	Bound	–	High	Engrailed	WT	3	300
30	Bound	–	Medium	Engrailed	WT	3	300
31	Bound	–	Low	Engrailed	WT	3	300
32	Bound	–	High	Distal-less	WT	3	300
33	Bound	–	Medium	Distal-less	WT	3	300
34	Bound	–	Low	Distal-less	WT	3	300

Table S3: Summary of all MDs simulations performed in this study. Each system was simulated in three independent replicas unless otherwise noted. Systems include forward and reverse orientations, four DNA core sequences (High, Medium High, Medium Low, Low), and multiple protein configurations (Hth-Exd-Scr trimer, Hth-Exd dimer, Exd-Scr dimer), as well as unbound DNA controls.

Comparison	Region	State A	State B	% Change	p-value
Unbound → Exd-Scr (Reverse)	Hth	1.35	1.27	-5.9%	0.263
Unbound → Exd-Scr (Reverse)	Spacer	1.22	1.12	-7.7%	0.275
Unbound → Exd-Scr (Reverse)	Exd_Scr	1.27	0.85	-33.2%	**0.002**
Exd-Scr → WT Trimer(Reverse)	Hth	1.27	0.91	-28.6%	**0.042**
Exd-Scr → WT Trimer(Reverse)	Spacer	1.12	1.03	-8.4%	0.357
Exd-Scr → WT Trimer(Reverse)	Exd_Scr	0.85	0.81	-4.7%	0.431
WT Trimer → Mutant(Reverse)	Hth	0.91	1.01	+11.7%	**0.049**
WT Trimer → Mutant(Reverse)	Spacer	1.03	1.08	+4.7%	0.740
WT Trimer → Mutant(Reverse)	Exd_Scr	0.81	0.81	-0.6%	0.935
Unbound → WT Trimer(Reverse)	Hth	1.35	0.91	-32.8%	**0.003**
Unbound → WT Trimer(Reverse)	Spacer	1.22	1.03	-15.4%	**0.033**
Unbound → WT Trimer(Reverse)	Exd_Scr	1.27	0.81	-36.4%	**0.003**
Unbound → Hth-Exd (Reverse)	Hth	1.35	0.91	-32.7%	**0.008**
Unbound → Hth-Exd (Reverse)	Spacer	1.22	1.09	-10.2%	0.342
Unbound → Hth-Exd (Reverse)	Exd_Scr	1.27	1.15	-10.1%	**0.030**
Unbound → Mutant(Reverse)	Hth	1.35	1.01	-25.0%	**0.020**
Unbound → Mutant(Reverse)	Spacer	1.22	1.08	-11.5%	0.376
Unbound → Mutant(Reverse)	Exd_Scr	1.27	0.81	-36.7%	**0.008**
Unbound → Exd-Scr (Forward)	Hth	1.33	1.32	-0.9%	0.731
Unbound → Exd-Scr (Forward)	Spacer	1.25	1.11	-11.4%	**0.030**
Unbound → Exd-Scr (Forward)	Exd_Scr	1.28	0.87	-32.3%	**0.013**
Exd-Scr → WT Trimer(Forward)	Hth	1.32	0.98	-25.5%	**0.042**
Exd-Scr → WT Trimer(Forward)	Spacer	1.11	1.08	-3.0%	0.598
Exd-Scr → WT Trimer(Forward)	Exd_Scr	0.87	0.83	-4.3%	0.638
Unbound → WT Trimer(Forward)	Hth	1.33	0.98	-26.2%	0.074
Unbound → WT Trimer(Forward)	Spacer	1.25	1.08	-14.0%	0.062
Unbound → WT Trimer(Forward)	Exd_Scr	1.28	0.83	-35.3%	**0.003**
Unbound → Hth-Exd (Forward)	Hth	1.33	1.05	-21.1%	0.056
Unbound → Hth-Exd (Forward)	Spacer	1.25	1.07	-14.8%	**0.004**
Unbound → Hth-Exd (Forward)	Exd_Scr	1.28	1.14	-11.4%	0.058

Table S4. Mean MGW-FL for the Hth core motif, spacer, and Exd–Scr core motif regions across simulated complexes. Comparisons include transitions from unbound DNA to dimers, wild-type trimer, and mutant trimer configurations in both reverse and forward orientations. State A and State B correspond to the first and second conditions listed for each comparison. Percent change reflects the relative difference in MGW fluctuation between states, and *p*-values are derived from two-tailed paired t-tests calculated across simulation replicates pooled across affinity classes. *P*-values < 0.05 are highlighted in bold.

SUPPLEMENTAL REFERENCES

1. Cooper, B., T.P. Chiu, and R. Rohs, 2022. Top-Down Crawl: a method for the ultra-rapid and motif-free alignment of sequences with associated binding metrics. *Bioinformatics*, **38**:5121–5123.
2. Abramson, J., J. Adler, J. Dunger, R. Evans, T. Green, A. Pritzel, O. Ronneberger, L. Willmore, A.J. Ballard, J. Bambrick, et al., 2024. Accurate structure prediction of biomolecular interactions with AlphaFold 3. *Nature*, **630**:493–500.
3. Azad, R.N., D. Zafiropoulos, D. Ober, Y. Jiang, T.P. Chiu, J.M. Sagendorf, R. Rohs, and T.D. Tullius, 2018. Experimental maps of DNA structure at nucleotide resolution distinguish intrinsic from protein-induced DNA deformations. *Nucleic Acids Res.*, **46**:2636–2647.
4. Mitra, R., J. Li, J.M. Sagendorf, Y. Jiang, A.S. Cohen, T.P. Chiu, C.J. Glasscock, and R. Rohs, 2024. Geometric deep learning of protein–DNA binding specificity. *Nat. Methods*, **21**:1674–1683.
5. Kribelbauer, J.F., R. Loker, S. Feng, C. Rastogi, N. Abe, H. Rube, H.J. Bussemaker, and R.S. Mann, 2020. Context-dependent gene regulation by homeodomain transcription factor complexes revealed by shape-readout deficient proteins. *Mol. Cell*, **78**:152–167.
