# Supplementary material for: Readout of intrinsic and induced DNA shape by homeodomain transcription factor complexes
Source: Biophys J. 2026 Mar 20;125(16):4345–56. doi: 10.1016/j.bpj.2026.03.036 (PMC13119616; doi:10.1016/j.bpj.2026.03.036)
Supplement: Supplementary file 2 [file mmc2.pdf]

Biophysical Journal
Article

Readout of intrinsic and induced DNA shape by homeodomain transcription factor complexes

Yibei Jiang,[1,9] Alexandra M. Shewchuk,[1] Tsu-Pei Chiu,[1] Jinsen Li,[1] Judith F. Kribelbauer-Swietek,[1,2] Nicolas Gompel,[3] and Remo Rohs[1,4,5,6,7,8,*]

[1]Department of Quantitative and Computational Biology, University of Southern California, Los Angeles, California; [2]Department of Biological Sciences, University of Southern California, Los Angeles, California; [3]Bonn Institute for Organismic Biology, University of Bonn, Bonn, Germany; [4]Department of Chemistry, University of Southern California, Los Angeles, California; [5]Department of Physics & Astronomy, University of Southern California, Los Angeles, California; [6]Thomas Lord Department of Computer Science, University of Southern California, Los Angeles, California; [7]Division of Medical Oncology, Department of Medicine, University of Southern California, Los Angeles, California; and [8]Alfred E. Mann Department of Biomedical Engineering, University of Southern California, Los Angeles, California
[9]Present address: Illumina, Inc., 5200 Illumina Way, San Diego, California

ABSTRACT Homeodomain transcription factors (TFs) recognize their DNA targets through both sequence-specific base contacts and readout of local DNA shape. Although intrinsic DNA structure is encoded by nucleotide sequence, it also undergoes protein-induced structural deformation upon binding. Yet, the interplay between intrinsic and protein-induced DNA shape remains unclear. Here, we dissect how these two readout modes determine binding specificity in a trimeric complex composed of the *Drosophila* Hox TF Sex combs reduced and its cofactors, Homothorax and Extradenticle. Guided by SELEX-seq data, we performed molecular dynamics simulations of this complex bound to sequences of varying binding affinities. We find that minor groove width reflects intrinsic DNA structure, whereas minor groove width fluctuations capture protein-induced stabilization and reshaping of the DNA. Within the trimeric complex, Homothorax reduces conformational fluctuations in an orientation- and sequence-dependent manner, with charged residues in its N-terminal arm playing key roles in DNA shape readout. This demonstrates that recognition involves a context-dependent balance between conformational selection and induced fit. We extend this analysis to two other homeodomain TFs, Distal-less and Engrailed, revealing that even closely related proteins produce distinct DNA shape signatures depending on sequence context. To translate these insights to novel sequences or mutant proteins, we evaluate whether AlphaFold 3 (AF3) can capture mutation-sensitive DNA shape readout. Although AF3 accurately reproduces wild-type structures, it struggles to predict how mutations or conformational dynamics alter DNA shape. To bridge this gap, we developed a hybrid pipeline integrating AlphaFold-based homology modeling, molecular dynamics simulations, and DeepPBS, a deep learning method for binding specificity prediction. This multiscale framework successfully captures the active modulation of DNA structure missed by AF3 alone, providing new insights into TF binding specificity and creating a roadmap for integrating deep learning and physics-based methods to study molecular mechanisms.

SIGNIFICANCE Transcription factors (TFs) are proteins that regulate gene expression through specific interactions with their DNA binding sites in the genome. Achieving a high degree of binding specificity often requires cofactors that interact with both the TF and the DNA, enabling recognition of intrinsic DNA shape features and inducing additional conformational changes that enhance binding stability and specificity. In this study, we introduce a pipeline that integrates biophysical simulations with deep learning methods to characterize DNA readout modes of homeodomain TFs, such as *Drosophila* Hox TFs in complex with two cofactors, Extradenticle and Homothorax. This pipeline extends to varied DNA sequences and mutant TFs, demonstrating the versatility of the approach and its usefulness in analyzing the mechanisms underlying TF-cofactor–DNA binding.

INTRODUCTION

Homothorax (Hth) is a homeodomain protein that plays a pivotal role in *Drosophila* embryonic development through Homeobox (Hox)-specific regulation of the expression of body-patterning genes (1–4). Together with Hox

Submitted September 29, 2025, and accepted for publication March 17, 2026.

*Correspondence: rohs@usc.edu

Editor Name: Ana Nicoleta Bondar

https://doi.org/10.1016/j.bpj.2026.03.036

© 2026 The Author(s). Published by Elsevier Inc. on behalf of Biophysical Society.
This is an open access article under the CC BY license (http://creativecommons.org/licenses/by/4.0/).

transcription factors (TFs) and Extradenticle (Exd), Hth forms a trimeric complex that binds specific DNA sequences to drive transcriptional programs required for segmental identity and other developmental processes (1,5–10). Exd enhances the DNA-binding specificity of Hox proteins (11,12), whereas Hth binds Exd to promote interactions with Hox TFs (9,13), stabilize the Exd-Hox complex, and facilitate the complex's nuclear localization (6,14). Understanding the molecular mechanisms underlying Hth-mediated DNA binding is essential for elucidating how Hox proteins achieve functional binding specificity.

Hth belongs to the class of TALE (Three Amino acid Loop Extension) homeodomain proteins, which also includes the human homologs MEIS1–3. Although several MEIS structures have been determined, none capture the trimeric Hth-Exd-Hox architecture that is central to *Drosophila* developmental regulation. This trimeric complex exhibits well-defined DNA-binding specificity (9), supported by extensive SELEX-seq data linking DNA shape features to binding affinity (15). We therefore focus on the *Drosophila* Hth-Exd-Hox system, using the human MEIS1 structure (PDB: 4XRM) as a template for homology modeling due to the high sequence similarity (see materials and methods).

Beyond sequence-specific recognition, homeodomain proteins also recognize DNA shape, with minor groove width (MGW) serving as a key structural determinant of protein–DNA binding specificity and complex stability (13,15). In particular, the N-terminal arms of these proteins, although intrinsically flexible, play a well-established role in minor groove recognition: their positively charged residues insert into the minor groove and contribute to DNA shape readout and binding specificity (15,16). SELEX-seq studies have shown that the Hth-Exd-Hox trimer, studied here with the Hox paralog Sex combs reduced (Scr), preferentially binds sequences with specific intrinsic MGW signatures with optimally spaced MGW minima (13). However, these studies were limited to nucleotide pentamers, in which DNA shape features were derived from pentamers with two nucleotide pairs adjacent to each central base pair (bp), and thus could not capture the extended length of DNA targets of the trimeric protein complex. Moreover, although these findings highlight the importance of intrinsic DNA shape features, the role of protein-induced conformational changes and dynamic fluctuations in bound DNA remains largely unexplored.

To address these gaps, we introduce MGW fluctuation (MGW-FL) as a metric for conformational flexibility. Unlike static MGW, which reflects intrinsic DNA structure, MGW-FL captures the stabilization induced by protein binding. We employed molecular dynamics (MD) simulations to investigate how Hth modulates both MGW and MGW-FL in the Hth-Exd-Scr–DNA complex and examined how mutations of the Hth N-terminal residues affect complex stability. To predict intrinsic DNA shape over extended regions, we used Deep DNAshape (17), a deep-learning-based approach for predicting three-dimensional DNA shape features without the limitations of earlier pentamer methods (18). Similarly, induced DNA deformations upon protein binding were previously inaccessible due to the lack of an experimentally solved structure of the trimeric complex. To overcome this limitation, we employed MD simulations to investigate how Hth modulates DNA shape through binding, focusing on the role of MGW and MGW-FL in stabilizing the Hth-Exd-Scr–DNA tertiary complex.

We further extended this analysis to understand how mutations in the Hth protein's N-terminal residues affect the stability of the complex, allowing us to identify shape-sensing residues critical for stabilization. Due to the lack of solved structures for mutants, we turned to structure prediction methods, particularly AlphaFold 3 (AF3) (19), which enables large-scale modeling of protein–DNA assemblies directly from sequence. Although AF3 performs well on wild-type (WT) and previously characterized structures, whether it can capture sequence- and mutation-dependent DNA shape features essential for binding specificity remains an open question.

Combining deep-learning-based structure prediction (AlphaFold), MD simulations, and mutation analysis offers a powerful strategy to investigate how the Hth-Exd-Scr complex and other homeodomain proteins achieve binding specificity through DNA shape recognition.

To complement our analysis of the Hth-Exd-Scr trimer, we also examined two single-homeodomain *Drosophila* TFs, Distal-less (Dll) and Engrailed (En). Despite sharing highly similar homeodomains, these proteins play opposing roles in development: Dll promotes leg development, whereas En mediates Hox-specific repression of leg fate on selected body segments (20). Comparing intrinsic DNA shape readout of these single-homeodomain proteins with the cofactor-dependent modulation of the Hth-Exd-Scr system illustrates how protein context shapes DNA recognition.

This integrated approach, combining deep-learning-based structural modeling and analysis with biophysical simulations, reveals how TFs recognize intrinsic DNA shape features and actively modulate DNA conformation to achieve binding specificity. With this framework, we provide new insights into the fundamental principles governing protein–DNA interactions.

MATERIALS AND METHODS

SELEX-seq analysis

We analyzed the SELEX-seq data from Kribelbauer et al. (13) for the 21-bp *hth_exd_dfd_wt_f* system, applying a threshold cutoff of 0.25 in relative binding affinity. Resulting sequences were aligned using Top-Down Crawl (21), a motif-free alignment method. Spacer sequences were identified through a −3 shift alignment, revealing a 4-bp spacer (e.g., TAAA) paired with the 16-mer core sequence (ATGATTAATGAC). Relative binding affinities, ranging from 0.67 to 0.30, were grouped into four bins: 0.67 ≥

$x > 0.60$ (bin 1), $0.60 \geq x > 0.50$ (bin 2), $0.50 \geq x > 0.40$ (bin 3), and $0.40 \geq x \geq 0.30$ (bin 4). Intrinsic DNA shape profiles for these sequence bins were calculated and visualized using the Deep DNAshape webserver (17,22). Finally, of the four DNA shape profiles across these four bins, we selected sequences representing the median shape values, as shown in Table S1, for subsequent MD simulations.

Initial structure for MD simulation

We modeled the *Drosophila* Hth-Exd-Scr–DNA complex using all-atom MD simulations. Since no experimental structure was available for the *Drosophila* Hth protein, we used its human homolog bound to the TGACAG core motif (PDB: 4XRM) as a template and employed AlphaFold 2 (AF2) (23) to predict the DNA-binding domain of the *Drosophila* Hth structure (UniProt: O46339). Both homologs exhibited high sequence and structural similarity. For the Exd-Hox protein dimer, with the Hox protein Sex combs reduced (Scr), bound to the ATGATTAAT motif, we used the co-crystal structure (PDB: 2R5Z), following the approach described in (24).

The initial simulation setup involved constructing a 21-bp DNA sequence using Deep DNAshape (17) (Table S1), preserving the Hth and Exd-Scr core motifs to match the DNA shape observed in the co-crystal structures. We repeated this process by varying the spacer sequences. Furthermore, the geometry of all constructed DNA fragments was refined and minimized using PHENIX (25) with its *geometry minimization* program. For the trimeric complexes, we assembled the three proteins onto DNA fragments by aligning their respective core motifs using PyMOL (26) to generate an initial structure for MD simulation. For other protein–DNA complex setups, we aligned the respective proteins as mentioned in the text. Residue mutations were introduced using PyMOL mutagenesis. The protein sequences used are provided in Table S2.

To assess the stability of protein–DNA contacts and to ensure that the main protein–DNA contacts are preserved in our MD simulations, we monitored the minimum heavy-atom distances between DNA and predefined DNA-interacting residues on each protein. Interface residues were selected based on DNAproDB (27) annotations (Hth: N321, N325, R328, R329, and I324) and previously reported structural determinants (15) for Scr (I47, Q50, N51, and M54) and Exd (N51, R55, and N7). Because these residues cluster spatially at the interface, measuring the smallest distance between any of them and the DNA provides an effective metric for continuous protein–DNA engagement (Fig. S1).

MD simulation protocol

The MD simulation protocol followed the procedure described in (24). All simulations were conducted using GROMACS 2020.3 (28,29) on NVIDIA Tesla A40/100 GPUs, employing the AMBER ff14SB force field (30) for protein parameterization and the Parmbsc1 force field (31) for DNA. This combination has been extensively validated and shown to reproduce DNA structure and protein–DNA interactions with high accuracy (31–34). Each complex was solvated in a triclinic box with a 15-Å buffer between the solute and the box edges using the explicit TIP3P water model (35). To neutralize the net charge and approximate physiological conditions, Na^+ and Cl^- ions were added to a final concentration of 150 mM with the GROMACS *genion* tool (28,29).

Each system was first subjected to 2000 steps of steepest descent energy minimization to optimize solvent distribution around the solute. This was followed by a multiphase equilibration protocol to prepare the system for production simulations. Specifically, three successive *NVT* (constant number of particles, volume, and temperature) equilibration steps, each 10 ps in duration, were performed to gradually raise the system temperature to 300 K. Subsequently, a 700-ps *NPT* (constant number of particles, pressure, and temperature) equilibration was conducted to stabilize the pressure. This step utilized a velocity-rescaling (*v*-rescale) thermostat and a Parrinello-Rahman barostat, with coupling time constants of $\tau_T = 0.1$ ps and $\tau_P = 1$ ps, respectively. Throughout the equilibration, the temperature and pressure were maintained at 300 K and 1 bar.

After equilibration, production simulations were carried out for 500 ns in the *NPT* ensemble at a temperature of 300 K and a pressure of 1 bar. A 2-fs integration time step was employed. The Verlet cutoff scheme was used for all calculations, with long-range electrostatic interactions computed via the particle mesh Ewald method (36) using a 12-Å cutoff. Nonbonded van der Waals interactions were also truncated at 12 Å. All bond lengths were constrained using the LINCS algorithm (37).

A complete overview of all systems that the simulations were based on, including DNA sequences, orientations, and protein components, is provided in Table S3.

Simulation convergence and replica consistency

Simulation convergence was assessed by monitoring the root-mean-square deviation (RMSD) of the full protein–DNA complex, which stabilized after $\sim$300 ns for all systems. RMSD traces for every simulation are provided (see data and code availability for details). Each system was simulated in triplicate, with replicas initialized using different random seeds to ensure independent sampling.

To further test whether our MGW and MGW-FL observations were dependent on the specific replica set used, we performed an additional, independently equilibrated set of three 500-ns MD simulations for a representative high-affinity system and analyzed the last 200 ns of each trajectory (Fig. S2, *A* and *B*). The MGW and MGW-FL profiles from this second replica set closely matched those from the original set, with high Pearson and Spearman's rank correlations. Together, these analyses confirm that both MGW shape readout and its local fluctuations are robust to replica choice and well converged under the simulation protocol used.

DNA shape calculation

MGW values were calculated using Curves 5.3 (38) from snapshots extracted every 100 ps from the MD trajectories. Average MGW profiles were analyzed and plotted using data from the final 300–500 ns of each simulation. MGW-FL was quantified as the pooled standard deviation of MGW across the same time window for three independent replicas.

Although predictive approaches such as Deep DNAshape (22) and related methods (19,39) can estimate DNA shape fluctuations by calculating the standard deviation across predicted values, these estimates reflect variability between predicted means rather than true conformational dynamics. Accurate quantification of conformational flexibility requires physics-based approaches, such as MD simulations, which explicitly model the underlying atomic motions.

DeepPBS predictions

We evaluated protein–DNA complexes using the DeepPBS method (40), which employs geometric deep learning to infer position weight matrices associated with DNA-binding specificity from a given structure of a complex. DeepPBS assigns relative importance (RI) scores to individual heavy atoms of protein residues, reflecting their contribution to binding specificity. These atom-level scores can be summed to produce residue-level importance values. For this study, we enabled the "*both readout*" configuration in DeepPBS and computed heavy-atom RI scores.

Residue location identification

Residue–DNA interactions were quantified from the equilibrated portion of each trajectory (final 300–500 ns), sampled every 100 ps. Distances between protein heavy atoms and DNA base atoms were computed, and

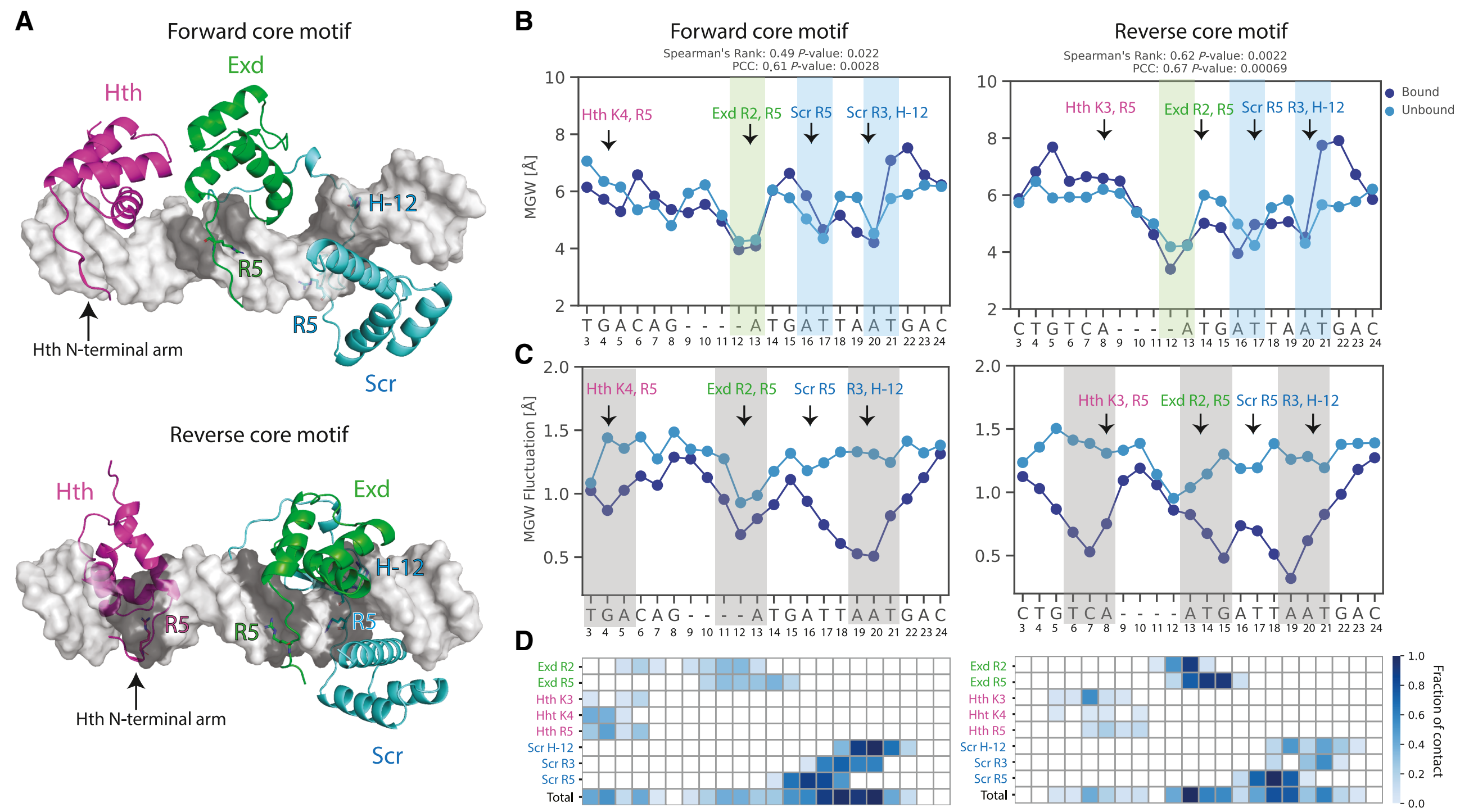

FIGURE 1 MGW and MGW-FL of the Hth-Exd-Hox–DNA ternary complex in forward and reverse orientations. (*A*) Structural configurations of Hth-Exd-Scr complexes bound to DNA core motifs in forward (*left*) and reverse (*right*) orientations. Key minor groove-interacting residues are shown as sticks. (*B*) Average MGW profiles for bound and unbound states in forward (*left*) and reverse (*right*) orientations. Color shaded regions indicate the location of MGW minima in regions where Hth (*magenta*), Exd (*green*), and Scr (*cyan*) bind. (*C*) MGW-FL profiles for bound and unbound states in forward (*left*) and reverse (*right*) orientations. Gray shaded regions indicate the locations of MGW-FL minima. (*D*) Relative positions of key minor groove-interacting residues are indicated based on the residue contact heatmap (described in materials and methods). In (*B*)–(*C*), the four dashes at the *x*-axis indicate the 4-bp spacer sequence.

contacts were defined as pairs within 3.5 Å. Contact frequencies were visualized as heatmaps. For lysine residues, the terminal amine group atoms were used: for arginine, the guanidino group atoms; and for histidine, the imidazole ring atoms. Only DNA base atoms were included in the analysis to capture base-specific contacts.

In the MGW and MGW-FL plots, residue positions were annotated based on their contact frequency. If no high-frequency contact was present (frequency >0.5), the midpoint of the contact distribution was used to assign the relative position of the protein residue. If multiple high-frequency contacts were present (frequency >0.8), the residue was assigned to the contact with the highest frequency. Total contacts were calculated as the sum of fractional contacts for all annotated residues in the heatmap.

Dynamic cross correlation matrix analysis

Dynamic cross correlation matrices (DCCMs) were used to quantify correlated and anticorrelated motions between residues within the Hth-Exd-Scr trimer. For each simulation, atomic positional covariance matrices were computed over the final 300−500 ns of the MD trajectories using C_α atoms only. Correlation coefficients range from 1 (fully correlated motion) to −1 (fully anticorrelated motion), with 0 indicating no dynamical coupling. Covariance matrices were generated using the *gmx covar* approach in the GROMACS package (28,29) and converted to residue-level DCCMs. For each DNA-binding orientation (forward and reverse), DCCMs were computed independently for all simulation replicas and then averaged element-wise to obtain a replica-averaged DCCM. To assess interprotein communication within the trimer, the averaged matrix was partitioned into Exd-Scr, Exd-Hth, and Scr-Hth blocks based on residue indices.

RESULTS

Context-dependent binding preferences revealed by DNA shape

We performed MD simulations of the Hth-Exd-Scr complex bound to a high-affinity DNA sequence (Fig. 1 *A*). Average MGW profiles over the final 200 ns (Fig. 1 *B*) were highly correlated between bound and unbound states, indicating that MGW in the complex is determined by the intrinsic sequence-dependent structure. In contrast, MGW-FL, which captures conformational flexibility (Fig. 1 *C*), was markedly reduced upon binding, particularly at contacts with positively charged residues such as Exd R5 and Scr R5/H–12 (Fig. 1, *C* and *D*). These reductions exceeded normal MD fluctuations, with decreases of 26%–36% at the Exd-Scr motifs compared with the unbound DNA for either core motif orientation (paired t-tests, $p < 0.05$). Full numerical values and statistical comparisons for each region are provided in Table S4, and the standard deviations across the three MD replicas for each system, for both MGW and MGW-FL, are shown in Fig. S2. These results show that MGW-FL serves as a signature of protein-induced stabilization, driven by charged residues that rigidify the DNA backbone.

Moreover, Hth binding orientation significantly influences the topology of the complex and TF cooperativity (13,41). Hth homodimers can bind DNA in both forward and reverse orientations (41), and our atomistic models show that this directional flexibility propagates through DNA to influence Exd-Scr binding (Fig. 1 *A*). In the reverse configuration, the Hth N-terminal arm—which is shown to use MGW readout (13)—is positioned closer to the Exd-Scr complex, shifting Exd toward Scr and producing a distinct decrease in MGW-FL at Exd contact sites (Fig. 1 *C*, right). We quantified these reductions in Table S4, which shows that the reverse core orientation produces larger and statistically stronger MGW-FL decreases at both the Hth and Exd-Scr motifs, with lower *p*-values compared with the forward orientation. Spacer regions retained unbound-like MGW-FL profiles, whereas core motifs with charged-residue contacts exhibited pronounced reductions (Fig. 1, *C* and *D*). These results demonstrate that orientation-specific binding affects local DNA conformation, with protein contacts actively remodeling the intrinsic DNA structure.

Comparative MD simulations of other homeodomain TFs, Engrailed (En) and Distal-less (Dll)—two highly similar but functionally antagonistic homeodomain TFs—further illustrate the interplay between intrinsic DNA shape and protein-induced modulation. Using the same set of high-, medium-, and low-affinity DNA sequences for both proteins, we observed that En and Dll induced different MGW patterns, with flanking-region variations most pronounced for high-affinity sequences and diminishing at lower affinities (Fig. S3). This indicates that intrinsic DNA structure provides the baseline shape while protein-specific interactions fine-tune it in a context-dependent manner.

Similarly, MGW-FL analysis further showed protein-induced stabilization near MGW minima compared with the unbound DNA (Fig. S4), and differences in MGW-FL profiles between En and Dll were most pronounced for high-affinity sequences. These MGW differences arise from protein–DNA interactions: compared with shape of the unbound DNA, the protein-bound form can adopt distinct conformations depending on the flanking-sequence context, resulting in different minor groove geometry, contact opportunities, and stability. Such flanking-sequence influences on MGW are well documented for homeodomain proteins, where regions adjacent to the core motif modulate DNA shape readout and functional specificity (24,42–44).

Collectively, these results demonstrate that MGW encodes intrinsic structural features of DNA, whereas MGW-FL captures protein-induced modulation of local flexibility. The alignment of MGW-FL reductions with contacting residues indicates coordinated stabilization of DNA dynamics. Moreover, the distinct MGW and MGW-FL patterns produced by different homeodomain proteins reveal that binding specificity is defined by the interplay between intrinsic DNA shape and protein-induced modulation.

Induced fit versus conformational selection in DNA recognition

The prior analysis revealed that MGW reflects intrinsic DNA structure, whereas MGW-FL captures protein-induced stabilization. Next, we sought to understand whether proteins select preexisting DNA conformations or actively remodel DNA upon binding by examining how DNA shape features vary across sequences of different binding affinities. We first analyzed intrinsic DNA shape preferences in SELEX-seq-derived spacer sequences by partitioning experimental sequences (13) into four affinity bins (see materials and methods) and predicting minor groove shape parameters using the Deep DNAshape webserver (22). High-affinity sequences showed a MGW minimum at the A13 position, whereas low-affinity sequences showed a maximum (Fig. 2 *A*). This indicates that high-affinity sites adopt preferred conformations in the absence of protein binding, consistent with a conformational selection mechanism. A similar preference was reported by Kribelbauer et al. (13), who observed MGW minima at this position, though their analysis was limited to DNA shape features that did not take into account the effect of nucleotides beyond nearest and next-nearest neighbors (i.e., a pentamer model) (45). Based on these results, we selected four representative sequences for MD simulations of unbound and bound states using complexes modeled through AF2-based homology modeling and structural superposition (see materials and methods).

MD simulations confirmed that intrinsic MGW profiles (Fig. 2 *B*) matched Deep DNAshape predictions: high-affinity sequences consistently placed A13 at a groove minimum, whereas low-affinity sequences placed it at a maximum (Fig. S5). High-affinity sequences exhibited narrower MGW and lower fluctuations, reflecting the intrinsic rigidity of AT-rich sequences, whereas GC-rich low-affinity sequences were more flexible (46).

Upon protein binding, MGW-FL is reduced globally, reflecting backbone stabilization (Figs. S5 and S6). To localize these effects, we compared MGW-FL distributions across DNA regions (Fig. 2 *C*). Bound complexes generally showed lower MGW-FL, consistent with protein-driven stabilization. In the Hth region, high-affinity sequences exhibited a pronounced shift toward lower MGW-FL values (Fig. 2 *C*, top, black arrows), reflecting stronger protein–DNA interactions and an induced fit. By contrast, the spacer region showed little difference between bound and unbound states (Fig. 2 *C*, center), supporting a conformational selection model in which proteins preferentially stabilize preexisting DNA conformations. The Exd-Scr core motif showed the strongest stabilization across all systems (Fig. 2 *C*, bottom), consistent with extensive contacts with charged residues.

Overall, these findings indicate that DNA recognition involves both conformational selection and induced fit, with

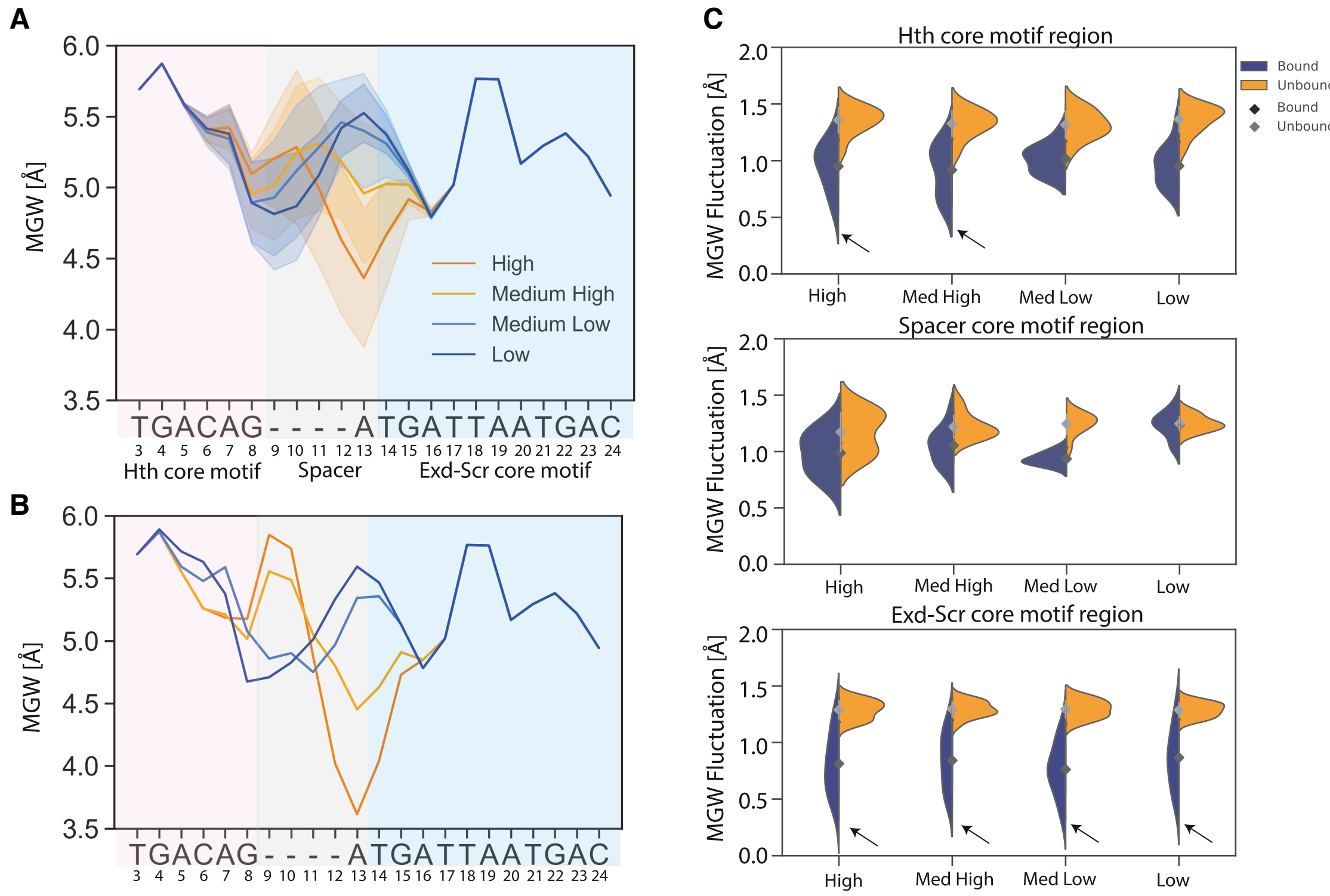

FIGURE 2 MGW and MGW-FL profiles across sequences of varying binding affinities. (*A*) Average MGW profiles for the Hth-Exd-Scr complex across four affinity categories: high, medium high, medium low, and low. (*B*) MGW profiles for four representative sequences selected based on the trends observed in (*A*). The Hth core motif region is shown in pink, the spacer region in gray, and the Exd-Scr region in blue. (*C*) MGW distributions across distinct functional regions of the DNA: the Hth core motif, spacer region, and Exd-Scr core motif, for the same four sequences of different affinities. Split violin plots compare MGW values in the bound versus unbound states. Arrows denote regions where binding likely suggests a potential protein-induced fit mechanism.

the balance between these mechanisms varying by sequence affinity and DNA region.

Evaluating bound DNA shape and mutation effects with AlphaFold 3

Although the preceding sections demonstrate that both intrinsic DNA shape and protein-induced modulation contribute to binding specificity, extending these insights to novel DNA sequences or mutant proteins requires computational structure prediction. We therefore evaluated whether AF3, an AlphaFold tool for modeling biomolecular complexes, can correlate DNA shape to TF–DNA binding. Our initial assessment showed that AF3-predicted complexes achieve a high degree of global structural accuracy. Comparing AF3 models of 11 selected proteins with their experimentally determined co-crystal structures showed a moderate correlation in MGW (average Pearson $r = 0.84$; Spearman's rank $\rho = 0.81$) (Figs. 3 *A* and S7). However, this high performance likely reflects the presence of these well-characterized complexes in AF3's training data rather than genuine predictive capability. To test this further, we compared AF3 predictions with MD simulations of the Hth-Exd-Scr trimer in complex with DNA. AF3 reproduced both forward and reverse orientations of the complex (Figs. S8, *A* and *B*), which is expected given that the corresponding crystal structure (PDB: 4XRM) was likely included in its training data. AF3 also captured approximate minor groove geometries, such as a narrower minor groove in high-affinity sequences and a wider minor groove in low-affinity ones (Fig. S9), but two critical limitations emerged.

First, AF3 fails to capture mutation effects. Mutations in shape-sensing residues produced MGW profiles nearly identical to complexes with WT protein (average Pearson $r = 0.99$) (Figs. 3 *A* and S10). This insensitivity indicates that AF3 reproduces average geometries based on structures present in its training data rather than modeling context-dependent structural variations that determine affinity and specificity. Interestingly, although AF3's MGW predictions were insensitive to mutations in shape-sensing residues in Hth, AF3's confidence scores decreased, particularly in the N-terminal arm (Figs. S8, *B* and *C*), suggesting that confidence may partially reflect the stabilizing role of these residues even when structural predictions do not.

Second, AF3 fails to model conformational fluctuations. To test whether sampling multiple AF3 predictions could recover MGW-FL, we generated three independent AF3 models per complex with different random seeds and computed pooled average MGW-FL across these

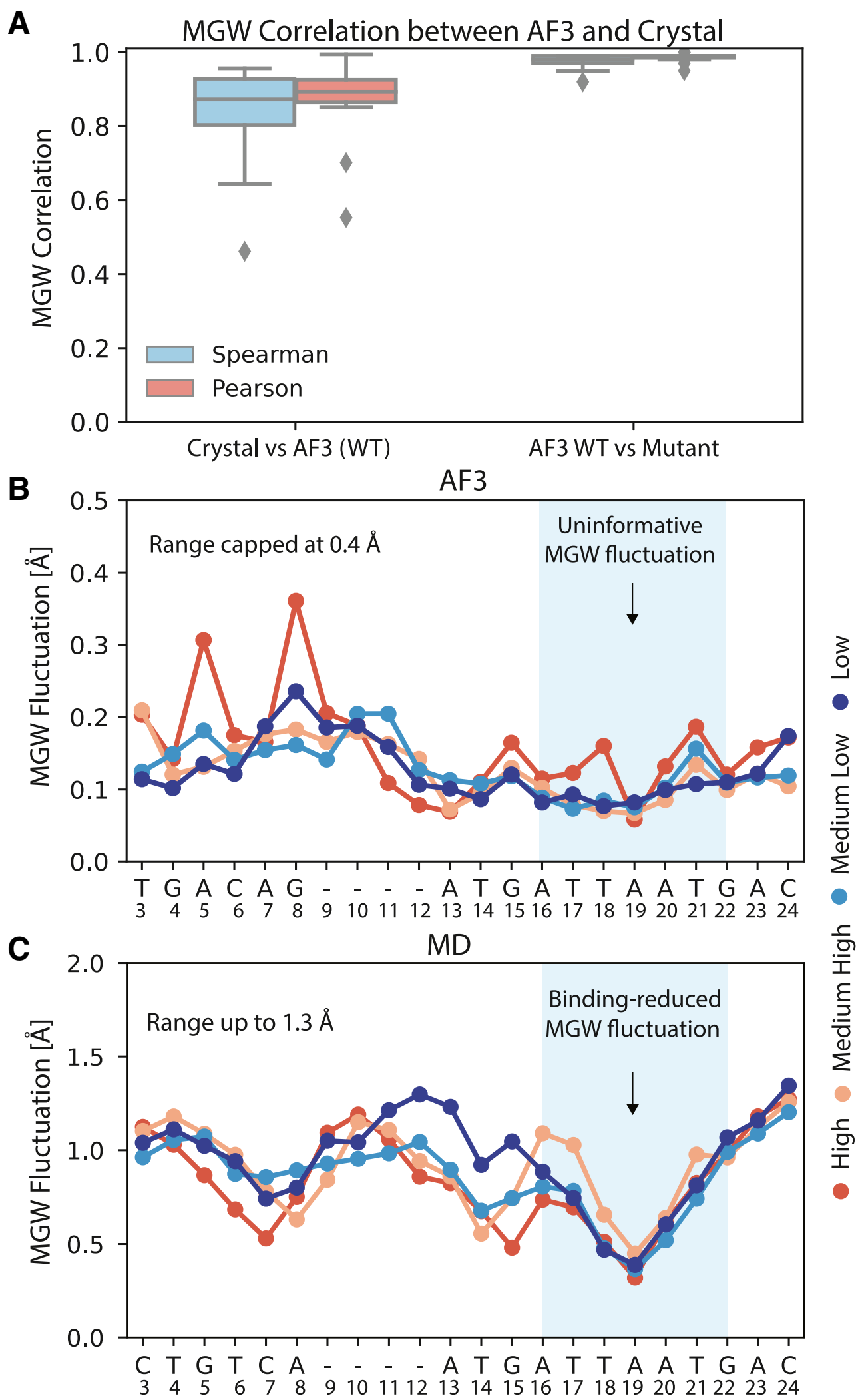

FIGURE 3 MD simulations reveal protein-coupled MGW dynamics not accessible to AF3 predictions. (*A*) Correlation between AF3-predicted MGW and co-crystal-derived MGW values (*left*) and between AF3-predicted MGW for WT and mutant complexes (*right*), showing agreement between AF3 predictions and experimental structures but limited responsiveness to mutations. (*B*) MGW-FL of AF3 predictions show flat and uninformative MGW-FL profiles across the core motif region. (*C*) In contrast, MD simulations reveal protein binding-induced reductions in MGW-FL within the core motif region.

predictions. All AF3-generated structures remained uniformly rigid, producing flat MGW-FL profiles across the core motif and failing to reproduce the position-specific minima observed in MD simulations. This indicates that AF3 model stochasticity does not substitute for explicit conformational dynamics (Fig. 3 *B*). In contrast, MD simulations revealed protein-coupled stabilization and reduced fluctuation at frequent contact sites, reflecting induced fit and binding stabilization rather than random variation (Fig. 3 C).

These results highlight a key limitation of deep learning methods for structure prediction that are trained on static structures: they cannot capture conformational flexibility or thermodynamic variability. In particular, the interplay between conformational selection and induced fit—which is essential to the binding mechanisms described above—requires modeling of conformational flexibility that AF3 cannot provide.

To overcome these limitations and capture both dynamics and mutation effects, we propose an AF–MD–DeepPBS workflow. To generate an initial scaffold, we leverage the speed of AlphaFold for the prediction of a first-pass structure. We chose to use AF2 in this workflow to predict the protein conformation only and construct complexes that retain the same DNA structures for the same DNA sequences. AF3 in comparison to AF2 aims primarily to predict complex assembly. To build initial complexes for MD simulation, we used homology modeling with AF3-predicted protein structures and rebuilt DNA structures (see materials and methods). These initial structures are then refined through MD simulation to capture induced fit and local fluctuations. Finally, DeepPBS (40) was applied to derive residue-level importance scores from structural and biophysical features, highlighting critical minor-groove-contacting residues (Figs. S11, S12, and S13; materials and methods). In the following sections, we apply this AF–MD–DeepPBS workflow to the Hth-Exd-Scr complex, demonstrating its utility in analyzing protein-induced DNA stabilization.

Hth-mediated stabilization and DNA shape modulation in Hth-Exd-Scr trimeric complex

Building on the AF–MD–DeepPBS pipeline, we investigated the role of Hth in stabilizing the Hth-Exd-Scr trimeric complex. Hth promotes Exd nuclear translocation and, together with Exd, enhances Hox–DNA binding specificity and affinity (9,39–41). To investigate Hth's contribution to DNA shape, we conducted MD simulations of four systems—Hth-Exd-Scr–DNA, Hth-Exd–DNA, Exd-Scr–DNA, and unbound DNA—and we compared their MGW-FL profiles. Across three replicates for each system, unbound DNA showed the highest fluctuations, serving as a baseline, whereas the full trimeric complex produced the strongest stabilization of MGW (Fig. 4).

To further dissect the combinatorial effects of Hth, Exd, and Scr, we analyzed their individual contributions, revealing synergistic effects. In the forward core orientation, the Hth-Exd complex exhibited high fluctuations in the Scr core motif region (TAAT), resembling unbound DNA (Fig. 4 *A*; Table S4), consistent with Scr's role in stabilizing this region. Removal of Scr (i.e., the Hth-Exd complex) also slightly increased MGW-FL across the Hth core motif (Fig. 4 *A*, red-shaded region; Table S4) and decreased RI scores in the Hth core motif region compared with the trimer (Fig. S14, *A* and *B*), underscoring the synergistic stabilization by all three proteins. Hth binding consistently decreased MGW-FL in its core motif region relative to

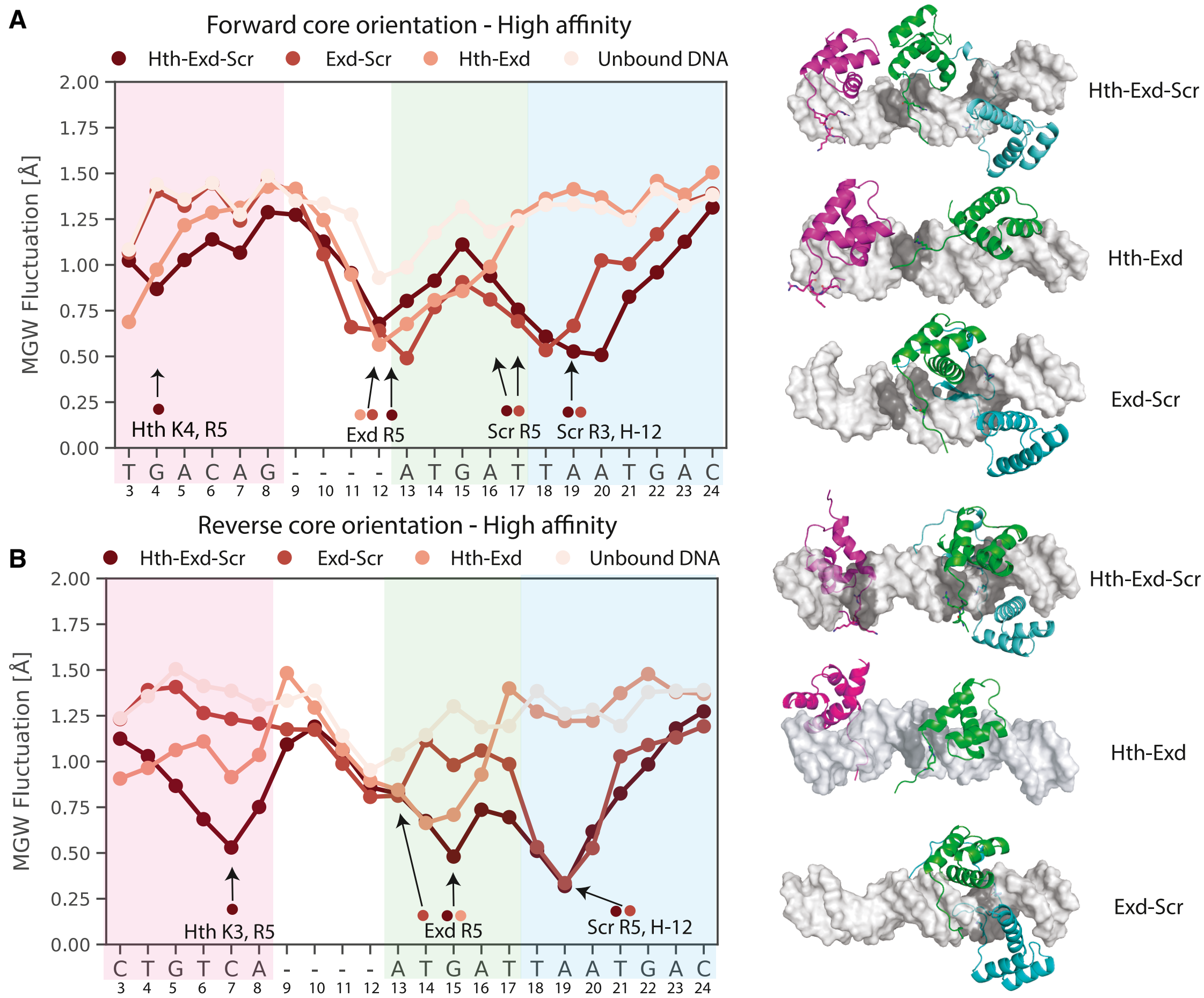

FIGURE 4 MGW-FL of Hth-Exd-Scr, Hth-Exd, and Exd-Scr complexes bound to high-affinity DNA sequences with core motifs in (*A*) forward and (*B*) reverse orientations. Unbound DNA is also shown for comparison. The positions of key residues are annotated based on residue contact analysis (Fig. S13). In (*A*) and (*B*), the initial structures of each complex are shown (*right*), with Hth in magenta, Exd in green and Scr in blue.

Exd-Scr or unbound DNA, with the effect more pronounced in low-affinity sequences (Fig. S15). This suggests a compensatory mechanism in which Hth reshapes DNA to stabilize weaker binding sites.

In addition to sequence affinity, core orientation further influences stabilization. In the forward orientation, Hth's N-terminal residues were positioned away from the complex, and MGW-FL in the Hth core region closely resembled unbound DNA (Fig. 4 *A*, red-shaded region), indicating limited stabilization. Consistent with this geometry, the contact maps in Figs. 1 *D* and S13 show interactions in the forward orientation localized toward the 5′ side of the DNA. In contrast, in the reverse orientation (Fig. 4 *B*, red-shaded region), closer N-terminal arm interactions produced greater MGW-FL stabilization, consistent with higher DeepPBS RI scores (Fig. S14, *B* and *C*) and a redistribution of Hth contacts toward the Exd region. Notably, repositioning of the Exd R5 residue near the Hox binding region shifted MGW minima by ∼2 nucleotides (A13 to G15 as seen in Fig. 4 *B*, green-shaded region), demonstrating that small positional shifts in key protein residues lead to measurable DNA conformational changes.

To further investigate cooperative behavior within the trimer, we computed replica-averaged DCCMs for the forward and reverse orientations. The DCCMs revealed distinct patterns of coordinated and anticorrelated motions across the three proteins (Fig. S16). In the forward orientation, the N-terminal arm of Scr showed strong positive correlation with Exd, consistent with direct contacts observed in MD snapshots and in the Scr-Exd co-crystal structure. Scr also exhibited weaker positive coupling with Hth through its N-terminal arm, whereas the Scr core and Hth displayed predominantly anticorrelated motions, reflecting DNA-mediated communication rather than direct contact. Exd and Hth showed minimal correlation, consistent with their lack of close interaction. In the reverse orientation, Scr-Exd coupling became more pronounced, whereas Hth exhibited largely anticorrelated motion in comparison to Exd and Scr, reflecting its repositioning relative to the DNA. Together, these results demonstrate that trimer specificity

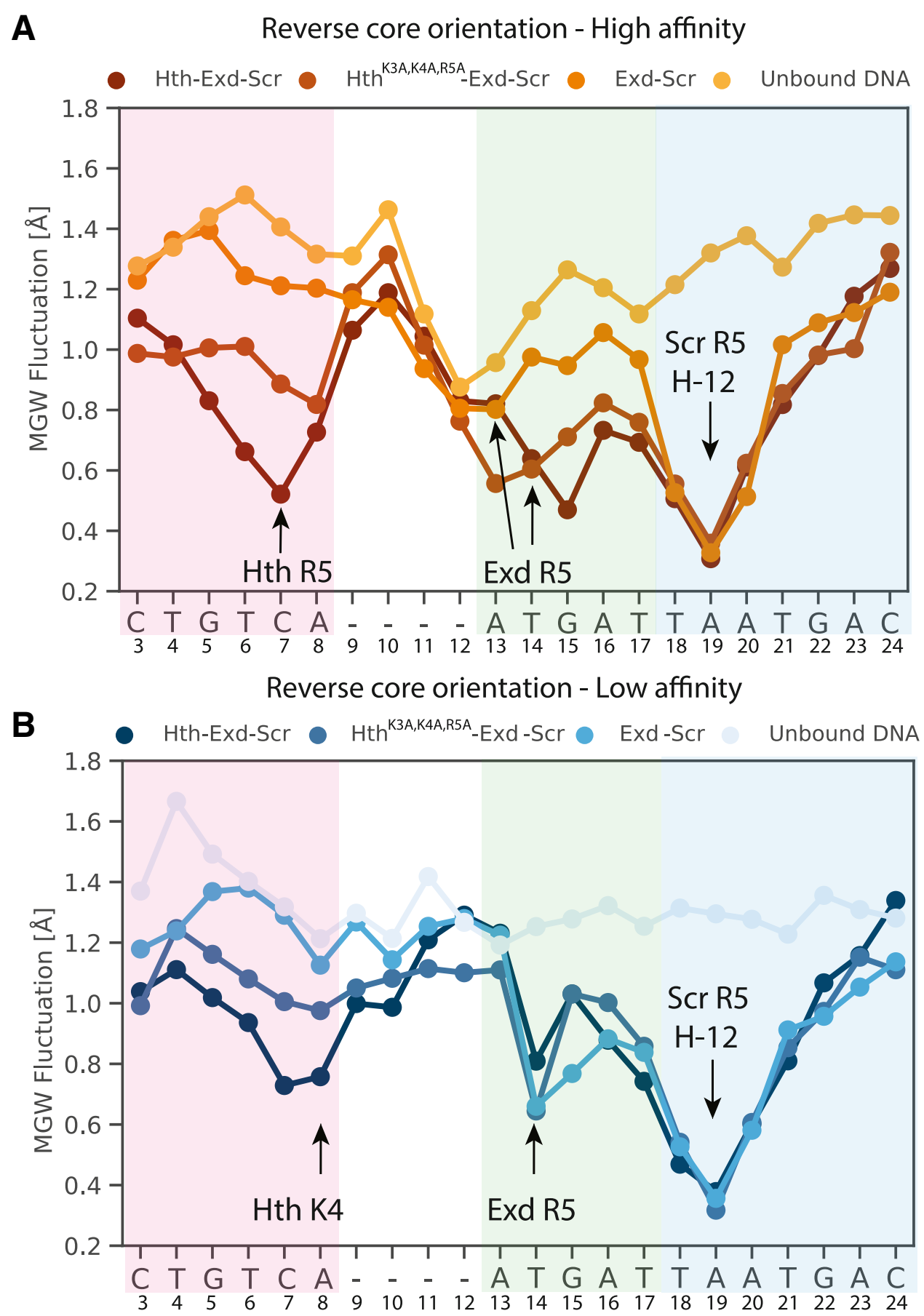

FIGURE 5 MGW-FL of Hth-Exd-Scr, $Hth^{K3A,4A,R5A}$-Exd, and Exd-Scr complexes bound to DNA sequences with the reverse core motif. Analyses focus on the reverse orientation, where the Hth N-terminal arm contacts the spacer and modulates protein–DNA interactions. The MGW profile of a high-affinity sequence is shown in (*A*) and low-affinity sequence in (*B*). The positions of key residues are annotated based on residue–DNA contacts during the MD simulations. Unbound DNA is also shown for comparison.

arises from the combined effects of DNA shape readout and motif-dependent cooperative dynamics across the protein–protein interfaces.

Finally, motif-level analysis showed that Hth enhances the overall binding specificity of the trimer (Fig. S17), with extension of the Hth core motif by six nucleotides further increasing specificity, reinforcing Hth's role in fine-tuning cooperative recognition.

Protein-induced impact on DNA conformation revealed by mutation simulations

The N-terminal arms of homeodomain proteins are highly dynamic yet play well-established roles in minor groove engagement and DNA shape readout (13,15,16,47). To test the functional importance of specific Hth residues, we introduced alanine substitutions at three N-terminal arm positions—K3, K4, and R5—selected based on prior experimental evidence implicating them in DNA shape readout (13). Because AF3 cannot reliably model mutant complexes (Fig. 3 *A*), we used the previous Hth-Exd-Scr–DNA structure as a template for MD simulations. The proteins were modeled with AF2 and superimposed on DNA (described in materials and methods). The resulting $Hth^{K3A,4A,R5A}$-Exd-Scr–DNA complex was compared with the WT trimeric complex, the Exd-Scr dimeric complex (Hth ablation), and unbound DNA. Mutations reduced MGW-FL stabilization at the Hth core motif (Fig. 5; Table S4), confirming the importance of K3, K4, and R5 in DNA shape recognition. Notably, the mutant trimer remained more stable than the Exd-Scr dimer, indicating that additional Hth–DNA interactions and Hth contacts with Exd or Scr contribute to binding affinity.

MGW-FL profiles further revealed that Hth mutations altered DNA shape at Exd binding sites: in high-affinity sequences, the MGW-FL minimum of the mutant shifted from G15 toward the spacer, resembling the Exd-Scr complex (Fig. 5 *A*), whereas in low-affinity sequences, it remained centered at T14. It is likely that in the low-affinity complex, the positioning of the Exd R5 residue is suboptimal, and its mutation does not result in changes in MGW-FL profile as seen in high-affinity sequences (Figs. 5 *B* and S13). Residue-level contact analysis using PLIP (48) confirmed that Exd residues (N54, I53, and R56–R58) maintained major groove interactions, whereas some Hth N-terminal residues (N1, Q2, and A3–A5) continued to form occasional minor groove contacts, explaining residual stabilization (Fig. S18).

To quantitatively evaluate changes in residue contributions upon mutation, we computed relative RI scores using DeepPBS, defining the change in RI between WT and mutant systems as $\Delta RI = RI_{\mathrm{Mutant}} - RI_{\mathrm{WT}}$. As expected, K3, K4, and R5 displayed reduced RI scores in the mutant complexes. At the global level, the mutant systems showed generally lower RI scores across the protein–DNA interface (Figs. S19 and S20), consistent with weakened binding. Nevertheless, some residues, such as Exd R5 and Scr major groove binding residues, exhibited increased RI scores, suggesting compensatory mechanisms that partially offset the loss of Hth N-terminal arm interactions, particularly in low-affinity sequence systems (Fig. S20).

Taken together, these results demonstrate that Hth's N-terminal arm residues are central to DNA shape readout and stabilization at the Exd interface. Their mutation weakens MGW-FL minima, resulting in reduced stabilization and altered DNA shape. Yet, cooperative interactions with Exd and Scr can in part compensate for the stabilizing effect of N-terminal Hth residues. The AF–MD–DeepPBS pipeline captured these mutation-dependent effects, whereas AF3 alone fails to capture them.

DISCUSSION

Our study highlights the multifaceted role of Hth in the Hth-Exd-Hox–DNA tertiary complex, showing that it stabilizes

protein–DNA interactions and modulates DNA shape. MD simulations and mutational analyses reveal that Hth contributes to binding stability by regulating MGW, with orientation- and sequence-dependent effects that reflect an interplay between intrinsic DNA structure and protein-induced modulation. A functional role of MGW-FL emerged: in unbound DNA, it mirrors intrinsic MGW, whereas in bound systems, it reflects protein-driven stabilization. The Hth N-terminal arm plays a key stabilizing role, as mutations at K3, K4, and R5 weakened minor groove stabilization and increased MGW-FL, consistent with prior work linking electrostatic interactions to minor groove stabilization (49).

In addition to these biological insights, our study evaluated the utility and limitations of AlphaFold-based prediction for protein–DNA complexes. AF2 has demonstrated high accuracy in protein modeling, including the recent discovery of a new fold for Forkhead proteins (50), but AF3 remains more limited for complexes that include nucleic acids (51). This limitation partly reflects differences in its training data: AF3's training data include far fewer DNA-containing structures (12,221 in total) compared with protein structures (235,334 in total; RCSB Protein Data Bank, August 2025), representing a two-order-of-magnitude imbalance. As a result, AF3 often reproduces bound DNA geometries that are already present in its training data but struggles to generalize to unseen or mutant complexes. Consistent with this, our mutation analyses revealed that AF3's predictions did not deviate from WT profiles, highlighting its difficulty in capturing mutation-induced conformational changes.

Beyond mutation effects, AF3 also falls short in representing induced structural adjustments upon binding. Although unbound DNA displays MGW profiles reflecting intrinsic DNA shape preferences, binding often induces DNA shape changes, with some proteins actively deforming DNA and others recognizing intrinsic geometries (52). Although AF3 reproduces aspects of bound DNA shape, this likely reflects training bias from similar deposited complexes rather than true inference of induced fit.

Overall, these observations motivate a hybrid strategy that integrates predictive modeling with physics-based simulations. For WT complexes lacking crystal structures, AF3 predictions can be paired with residue-level scoring methods such as DeepPBS to evaluate protein–DNA interactions. For mutants, where relevant structural data are largely absent from training data, MD simulations become essential: AlphaFold-generated complexes can serve as starting models for MD simulations, followed by MD simulations and quantitative analysis with DeepPBS or related tools. When an experimental co-crystal structure is available, AF2 can be used to model the protein and align it to the DNA in the crystal structure; otherwise, AF3 can generate the full complex. This workflow leverages AlphaFold's speed and accessibility for rapid model generation while addressing its limitations in capturing mutation effects and DNA conformational flexibility. By integrating MD simulations, mutation analysis, and DeepPBS, our framework enables systematic dissection of residue-level contributions to DNA binding specificity and stability. Future applications may extend this approach to explore the roles of additional cofactors, DNA variants, and post-translational modifications in shaping protein–DNA recognition.

Conclusion

Our study confirms that DNA shape is a critical determinant of DNA recognition and binding specificity of many homeodomain proteins. Through MD simulations and mutation analyses, we demonstrate that the Hth-Exd-Scr complex stabilizes DNA conformation by modulating MGW and its MGW-FL in a sequence- and orientation-dependent manner. Rather than being governed solely by conformational selection or induced fit, DNA recognition emerges from a context-dependent balance between the two. These findings underscore that protein–DNA recognition is not dictated by a single mechanism but instead reflects the interplay of multiple, overlapping factors. Mutational analyses further reveal that Hth shape-readout residues (K3, K4, and R5) influence MGW both locally at the Hth binding site and globally across the complex, with evidence of compensatory stabilization by neighboring Exd and Scr residues.

Beyond these biological insights, our evaluation of AlphaFold-based predictions highlights both their utility and limitations. Although AF3 provides rapid first-pass models for WT complexes, it struggles to capture mutation-induced conformational changes and fails to capture dynamic groove fluctuations. To address these gaps, we propose an integrated AF–MD–DeepPBS pipeline: using AF2 to model proteins and generate initial complexes, MD to simulate conformational ensembles, and DeepPBS to quantify residue-level contributions. This framework overcomes the static nature of AlphaFold-like models and enables systematic evaluation of mutation effects on DNA shape readout. In cases where no structural information is available, AF3 can still serve as a practical starting point and, when paired with DeepPBS, can provide actionable residue-level insights even in the absence of higher-resolution models.

DATA AND CODE AVAILABILITY

RMSD trajectories for all MD simulations are available in a zenodo repository: https://doi.org/10.5281/zenodo.17694015.

ACKNOWLEDGMENTS

The authors thank Brendon H. Cooper for advice on Top-Down Crawl, Liucong Ling for assistance with the selection of DNA fragments for MD simulations, Raktim Mitra for advice on DeepPBS, Rosa Di Felice for advice

on MD, and all members of the Rohs lab for valuable suggestions. This work was supported by the National Institutes of Health (R35GM130376 to R.R.), a University of Southern California Office of Research and Innovation SBIR/STTR Planning Award (to R.R.), an Andrew J. Viterbi Fellowship in Computational Biology and Bioinformatics (to A.M.S.), and the Deutsche Forschungsgemeinschaft (grant GO 2495/16-1 to N.G.).

AUTHOR CONTRIBUTIONS

Project conception: Y.J., T.P.C., and R.R. Lead project design: Y.J., T.P.C., and R.R. Supporting project design A.M.S., J.L., J.F.K.S., and N.G. Data generation and analysis: Y.J. Manuscript writing: Y.J. and A.M.S. Manuscript edits: Y.J., A.M.S., T.P.C., J.L., J.F.K.S., N.G., and R.R. Project supervision: R.R.

DECLARATION OF INTERESTS

The authors declare no competing interests.

SUPPORTING MATERIAL

Supporting Material can be found online at https://doi.org/10.1016/j.bpj.2026.03.036.

REFERENCES

1. Rieckhof, G. E., F. Casares, …, R. S. Mann. 1997. Nuclear translocation of extradenticle requires homothorax, which encodes an extradenticle-related homeodomain protein. *Cell.* 91:171–183.
2. Aldaz, S., G. Morata, and N. Azpiazu. 2005. Patterning function of homothorax/extradenticle in the thorax of Drosophila. *Development.* 132:439–446.
3. Agrawal, P., F. Habib, …, L. S. Shashidhara. 2011. Genome-level identification of targets of Hox protein Ultrabithorax in Drosophila: novel mechanisms for target selection. *Sci. Rep.* 1:205.
4. Kurant, E., D. Eytan, and A. Salzberg. 2001. Mutational analysis of the Drosophila homothorax gene. *Genetics.* 157:689–698.
5. Ladam, F., and C. G. Sagerström. 2014. Hox regulation of transcription: more complex(es). *Dev. Dyn.* 243:4–15.
6. Jaw, T. J., L.-R. You, …, Y. H. Sun. 2000. Direct interaction of two homeoproteins, homothorax and extradenticle, is essential for EXD nuclear localization and function. *Mech. Dev.* 91:279–291.
7. Wang, X., T. Zhou, …, R. Rohs. 2018. Analysis of genetic variation indicates DNA shape involvement in purifying selection. *Mol. Biol. Evol.* 35:1958–1967.
8. Moens, C. B., and L. Selleri. 2006. Hox cofactors in vertebrate development. *Dev. Biol.* 291:193–206.
9. Ryoo, H. D., T. Marty, …, R. S. Mann. 1999. Regulation of Hox target genes by a DNA bound Homothorax/Hox/Extradenticle complex. *Development.* 126:5137–5148.
10. Gebelein, B., J. Culi, …, R. S. Mann. 2002. Specificity of Distalless repression and limb primordia development by abdominal Hox proteins. *Dev. Cell.* 3:487–498.
11. Mann, R. S., and S.-K. Chan. 1996. Extra specificity from extradenticle: the partnership between HOX and PBX/EXD homeodomain proteins. *Trends Genet.* 12:258–262.
12. Singh, A., B. Acharya, …, S. De. 2024. Stability and dynamics of extradenticle modulates its function. *Curr. Res. Struct. Biol.* 7:100150.
13. Kribelbauer, J. F., R. E. Loker, …, R. S. Mann. 2020. Context-dependent gene regulation by homeodomain transcription factor complexes revealed by shape-readout deficient proteins. *Mol. Cell.* 78:152–167.e11.
14. Azpiazu, N., and G. Morata. 1998. Functional and regulatory interactions between Hox andextradenticle genes. *Genes Dev.* 12:261–273.
15. Joshi, R., J. M. Passner, …, R. S. Mann. 2007. Functional specificity of a Hox protein mediated by the recognition of minor groove structure. *Cell.* 131:530–543.
16. Abe, N., I. Dror, …, R. S. Mann. 2015. Deconvolving the recognition of DNA shape from sequence. *Cell.* 161:307–318.
17. Li, J., T. P. Chiu, and R. Rohs. 2024. Predicting DNA structure using a deep learning method. *Nat. Commun.* 15:1243.
18. Wang, Y., J. Li, …, R. Rohs. 2025. DNAdesign: feature-aware in silico design of synthetic DNA through mutation. *Bioinformatics.* 41:btaf052.
19. Abramson, J., J. Adler, …, J. M. Jumper. 2024. Accurate structure prediction of biomolecular interactions with AlphaFold 3. *Nature.* 630:493–500.
20. Gebelein, B., D. J. McKay, and R. S. Mann. 2004. Direct integration of Hox and segmentation gene inputs during Drosophila development. *Nature.* 431:653–659.
21. Cooper, B. H., T. P. Chiu, and R. Rohs. 2022. Top-Down Crawl: a method for the ultra-rapid and motif-free alignment of sequences with associated binding metrics. *Bioinformatics.* 38:5121–5123.
22. Li, J., and R. Rohs. 2024. Deep DNAshape webserver: prediction and real-time visualization of DNA shape considering extended k-mers. *Nucleic Acids Res.* 52:W7–W12.
23. Jumper, J., R. Evans, …, D. Hassabis. 2021. Highly accurate protein structure prediction with AlphaFold. *Nature.* 596:583–589.
24. Jiang, Y., T. P. Chiu, …, R. Rohs. 2024. Probing the role of the protonation state of a minor groove-linker histidine in Exd-Hox–DNA binding. *Biophys. J.* 123:248–259.
25. Adams, P. D., P. V. Afonine, …, R. W. Grosse-Kunstleve. 2010. PHENIX: a comprehensive Python-based system for macromolecular structure solution. *Acta Crystallogr. Sect. D Biol. Crystallogr.* 66:213–221.
26. Schrödinger, L. L. C. 2015. The PyMOL Molecular Graphics System, Version 1.8. https://www.pymol.org.
27. Mitra, R., A. S. Cohen, …, R. Rohs. 2025. DNAproDB: an updated database for the automated and interactive analysis of protein–DNA complexes. *Nucleic Acids Res.* 53:D396–D402.
28. Smith, L. J., X. Daura, and W. F. van Gunsteren. 2002. Assessing equilibration and convergence in biomolecular simulations. *Proteins.* 48:487–496.
29. Daura, X., W. F. van Gunsteren, and A. E. Mark. 1999. Folding–unfolding thermodynamics of a β-heptapeptide from equilibrium simulations. *Proteins.* 34:269–280.
30. Maier, J. A., C. Martinez, …, C. Simmerling. 2015. ff14SB: improving the accuracy of protein side chain and backbone parameters from ff99SB. *J. Chem. Theory Comput.* 11:3696–3713.
31. Ivani, I., P. D. Dans, …, M. Orozco. 2016. Parmbsc1: a refined force field for DNA simulations. *Nat. Methods.* 13:55–58.
32. Battistini, F., A. Hospital, …, M. Orozco. 2019. How B-DNA dynamics decipher sequence-selective protein recognition. *J. Mol. Biol.* 431:3845–3859.
33. Hörberg, J., and A. Reymer. 2020. Specifically bound BZIP transcription factors modulate DNA supercoiling transitions. *Sci. Rep.* 10:18795.
34. Dans, P. D., I. Ivani, …, M. Orozco. 2017. How accurate are accurate force-fields for B-DNA? *Nucleic Acids Res.* 45:4217–4230.
35. Jorgensen, W. L., J. Chandrasekhar, …, M. L. Klein. 1983. Comparison of simple potential functions for simulating liquid water. *J. Chem. Phys.* 79:926–935.
36. Darden, T., D. York, and L. Pedersen. 1993. Particle mesh Ewald: An N·log (N) method for Ewald sums in large systems. *J. Chem. Phys.* 98:10089–10092.
37. Hess, B., H. Bekker, …, J. G. E. M. Fraaije. 1997. LINCS: A linear constraint solver for molecular simulations. *J. Comput. Chem.* 18:1463–1472.

38. Lavery, R., and H. Sklenar. 1988. The definition of generalized helicoidal parameters and of axis curvature for irregular nucleic acids. *J. Biomol. Struct. Dyn.* 6:63–91.
39. Wohlwend, J., G. Corso, …, R. Barzilay. 2025. Boltz-1 democratizing biomolecular interaction modeling. Preprint at bioRxiv. https://doi.org/10.1101/2024.11.19.624167.
40. Mitra, R., J. Li, …, R. Rohs. 2024. Geometric deep learning of protein–DNA binding specificity. *Nat. Methods.* 21:1674–1683.
41. Jolma, A., Y. Yin, …, J. Taipale. 2015. DNA-dependent formation of transcription factor pairs alters their binding specificity. *Nature.* 527:384–388.
42. Chiu, T. P., J. Li, …, R. Rohs. 2022. It is in the flanks: Conformational flexibility of transcription factor binding sites. *Biophys. J.* 121:3765–3767.
43. Gordân, R., N. Shen, …, M. L. Bulyk. 2013. Genomic regions flanking E-box binding sites influence DNA binding specificity of bHLH transcription factors through DNA shape. *Cell Rep.* 3:1093–1104.
44. Ghoshdastidar, D., and M. Bansal. 2022. Flexibility of flanking DNA is a key determinant of transcription factor affinity for the core motif. *Biophys. J.* 121:3987–4000.
45. Zhou, T., L. Yang, …, R. Rohs. 2013. DNAshape: a method for the high-throughput prediction of DNA structural features on a genomic scale. *Nucleic Acids Res.* 41:W56–W62.
46. Oguey, C., N. Foloppe, and B. Hartmann. 2010. Understanding the sequence-dependence of DNA groove dimensions: implications for DNA interactions. *PLoS One.* 5:e15931.
47. Slattery, M., T. Riley, …, R. S. Mann. 2011. Cofactor binding evokes latent differences in DNA binding specificity between Hox proteins. *Cell.* 147:1270–1282.
48. Adasme, M. F., K. L. Linnemann, …, M. Schroeder. 2021. PLIP 2021: expanding the scope of the protein–ligand interaction profiler to DNA and RNA. *Nucleic Acids Res.* 49:W530–W534.
49. Rohs, R., X. Jin, …, R. S. Mann. 2010. Origins of Specificity in Protein-DNA Recognition. *Annu. Rev. Biochem.* 79:233–269.
50. Cooper, B. H., A. C. Dantas Machado, …, R. Rohs. 2023. DNA binding specificity of all four Saccharomyces cerevisiae forkhead transcription factors. *Nucleic Acids Res.* 51:5621–5633.
51. Wang, G. L., Y. Jiang, …, R. Rohs. 2025. Novel fold and wing structure of Forkhead transcription factor facilitate DNA binding. *Nucleic Acids Res.* 53:gkaf946.
52. Azad, R. N., D. Zafiropoulos, …, T. D. Tullius. 2018. Experimental maps of DNA structure at nucleotide resolution distinguish intrinsic from protein-induced DNA deformations. *Nucleic Acids Res.* 46:2636–2647.

Biophysical Journal, Volume *125*

Supplemental information

Readout of intrinsic and induced DNA shape by homeodomain transcription factor complexes

Yibei Jiang, Alexandra M. Shewchuk, Tsu-Pei Chiu, Jinsen Li, Judith F. Kribelbauer-Swietek, Nicolas Gompel, and Remo Rohs

SUPPLEMENTAL INFORMATION

Readout of induced and intrinsic DNA shape by homeodomain transcription factor complexes

Yibei Jiang[1,†], Alexandra M. Shewchuk[1], Tsu-Pei Chiu[1], Jinsen Li[1], Judith F. Kribelbauer-Swietek[1,2], Nicolas Gompel[3], and Remo Rohs[1,4,5,6,7,8,*]

[1]Department of Quantitative and Computational Biology, University of Southern California, Los Angeles, CA 90089, USA

[2]Department of Biological Sciences, University of Southern California, Los Angeles, CA 90089, USA

[3]Bonn Institute for Organismic Biology, University of Bonn, 53115 Bonn, Germany

[4]Department of Chemistry, University of Southern California, Los Angeles, CA 90089, USA

[5]Department of Physics & Astronomy, University of Southern California, Los Angeles, CA 90089, USA

[6]Thomas Lord Department of Computer Science, University of Southern California, Los Angeles, CA 90089, USA

[7]Division of Medical Oncology, Department of Medicine, University of Southern California, Los Angeles, CA 90033, USA

[8]Alfred E. Mann Department of Biomedical Engineering, University of Southern California, Los Angeles, CA 90089, USA

[†]Present address: Illumina, Inc., 5200 Illumina Way, San Diego, CA 92122, USA

*To whom correspondence should be addressed. Tel: +1 213 740 0552; Email: rohs@usc.edu

SUPPLEMENTAL FIGURES

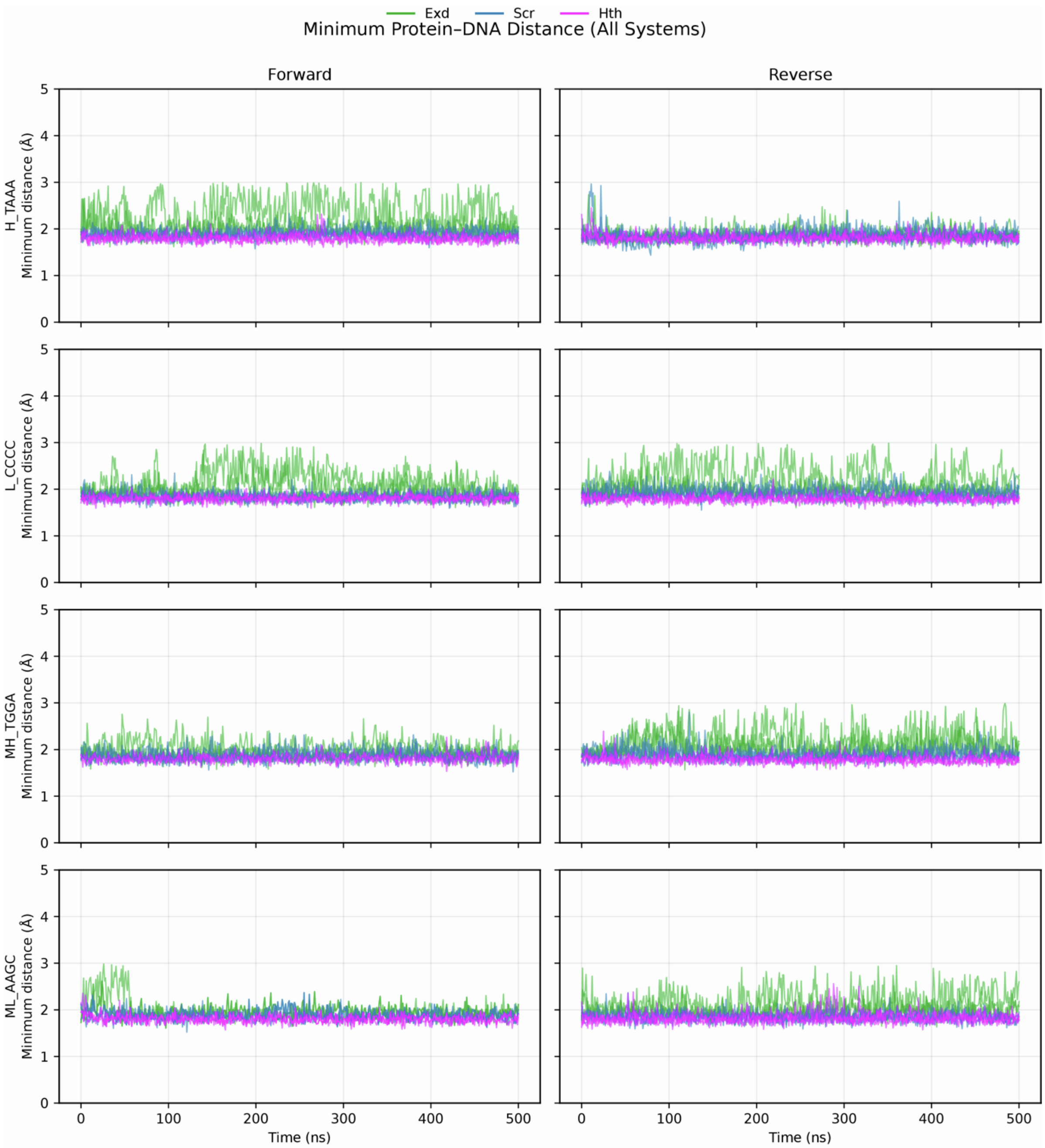

Fig. S1: Time-series traces show the minimum heavy-atom distance (Å) between the DNA and interface residues of Hth, Exd, and Scr across 500-ns simulations for each sequence variant in the forward (left) and reverse (right) orientations. Three replicas are overlaid for each system, with distances from the three proteins shown in green, blue, and magenta.

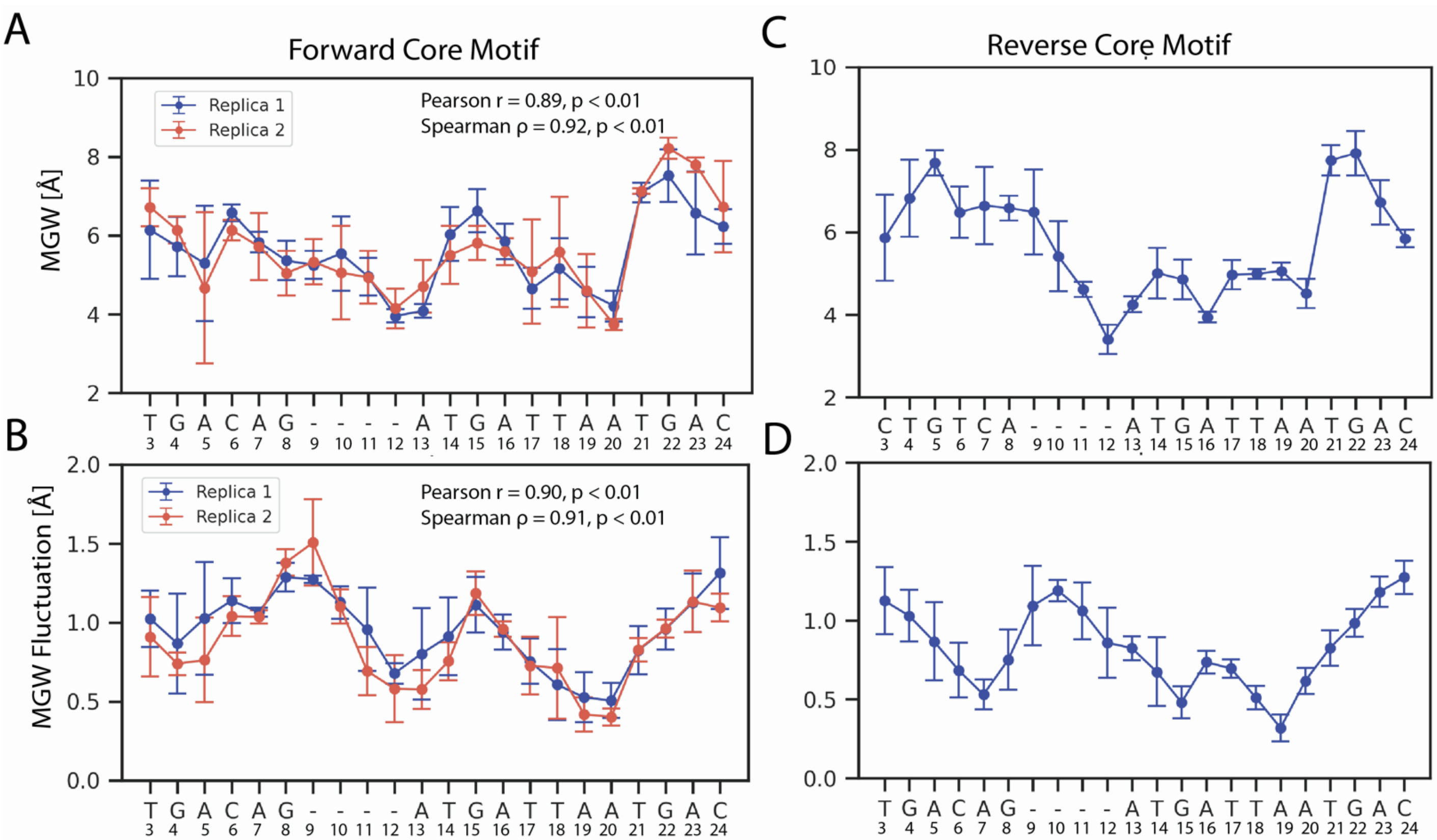

Fig. S2. Convergence of minor groove width (MGW) and MGW fluctuation (MGW-FL) across independently equilibrated MD replicas. (A) MGW profiles for the Forward core motif, comparing two independently equilibrated replica sets (blue, orange), each consisting of three 500-ns simulations; the last 200 ns of each simulation is shown. The two replica sets exhibit highly similar MGW patterns (Pearson $r = 0.89$, $p < 0.01$; Spearman's rank $\rho = 0.92$, $p < 0.01$). (B) Corresponding MGW-FL, computed as the pooled MGW standard deviation within each replica set, also shows strong agreement between replicas (Pearson $r = 0.90$, $p < 0.01$; Spearman's rank $\rho = 0.91$, $p < 0.01$). Error bars indicate variability among the three simulations within each replica set. (C) MGW profile for the high-affinity Reverse core motif. (D) Corresponding MGW-FL profile for system in (C). In panels (C–D), error bars represent variability among the three replicate simulations

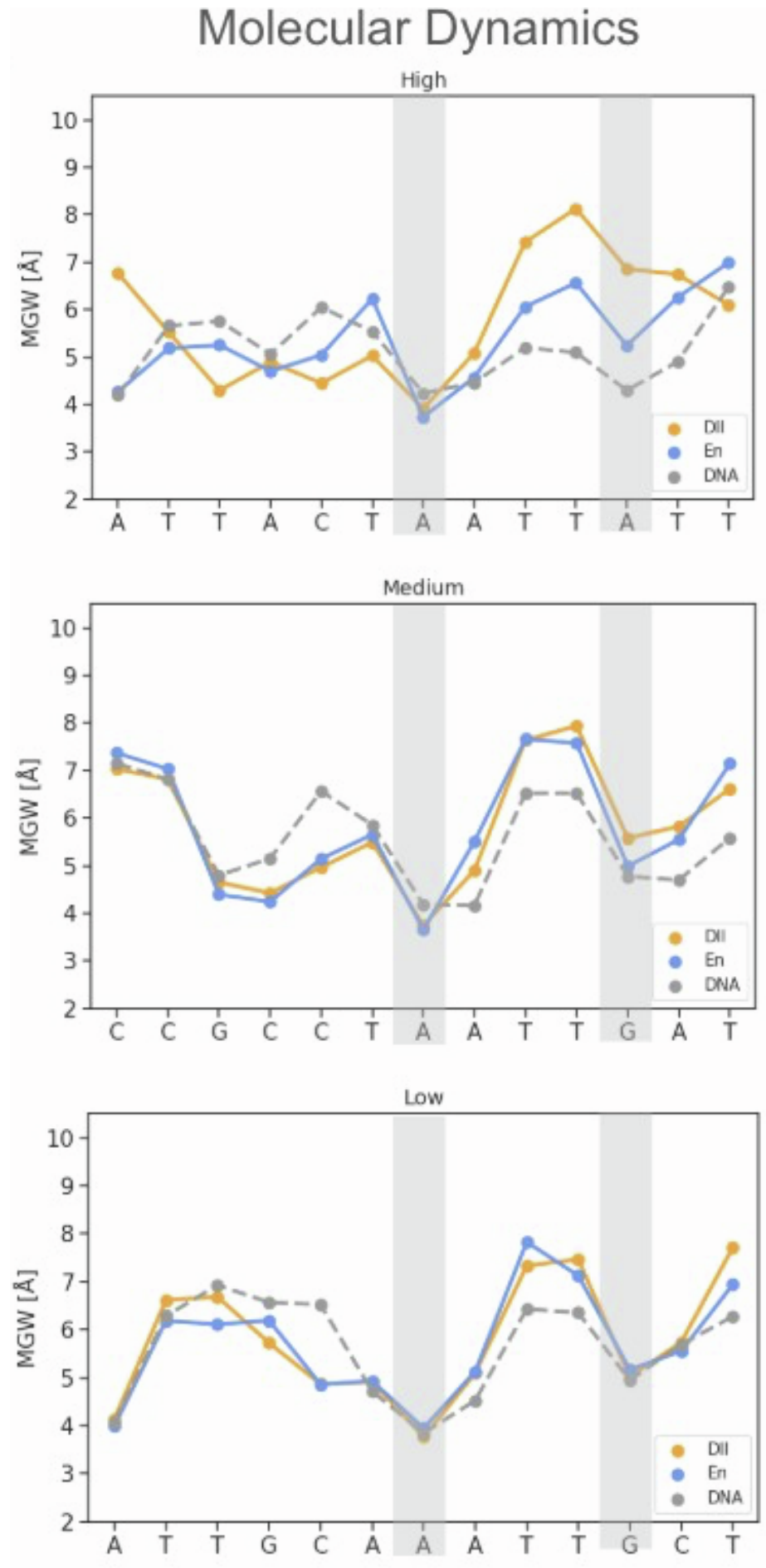

Fig. S3: DNA sequence profiles for various nucleotide sequences bound by Engrailed (En) and Distal-less (Dll) homeodomain proteins. Average DNA shape profiles calculated from MD simulations for three selected nucleotide sequences with relative binding affinities derived from SELEX-seq binding assays (High, Medium, and Low binding affinity). The DNA profile for each sequence bound to Dll (orange), En (blue), or unbound DNA (gray) is shown in each sequence

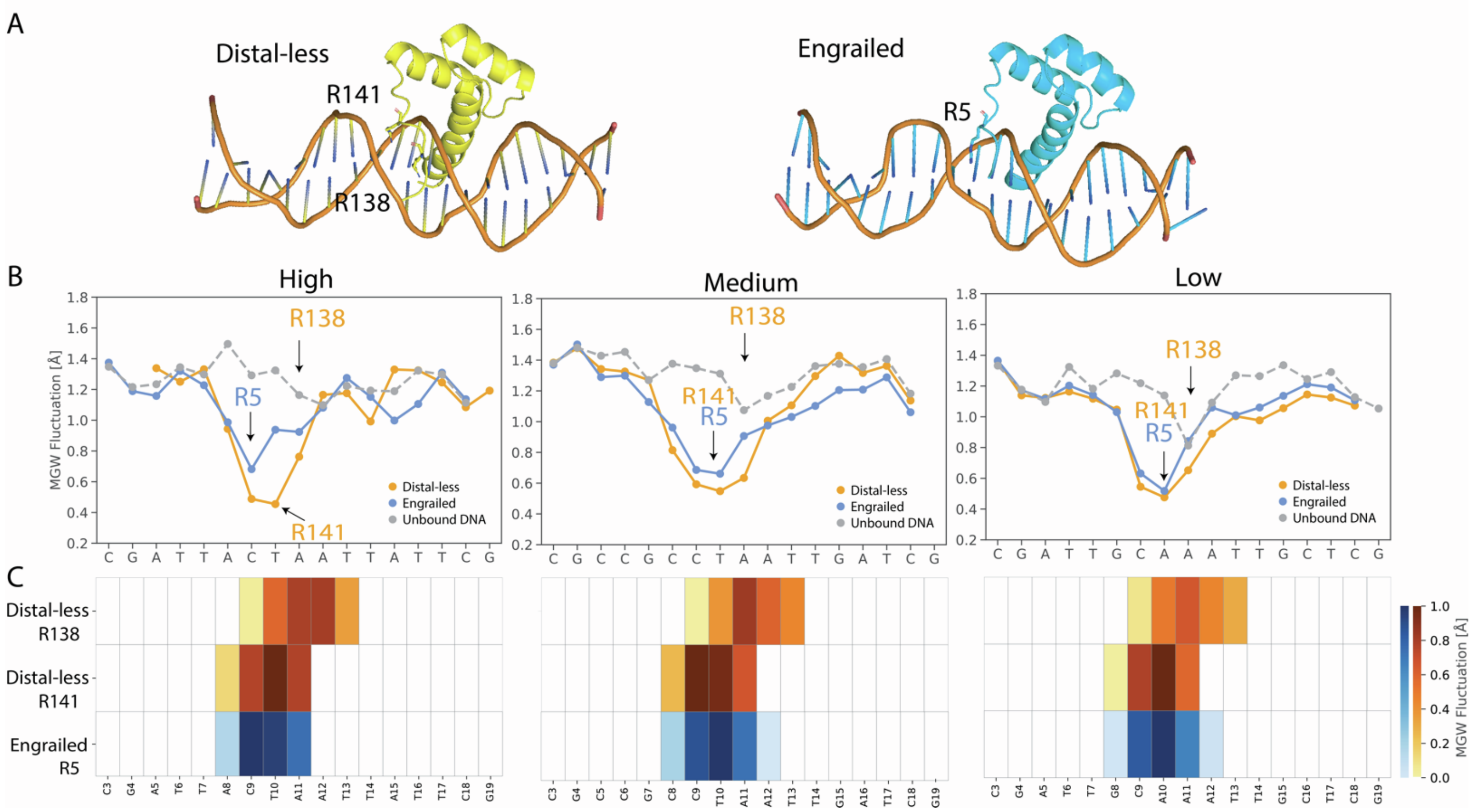

Fig. S4: Minor groove width fluctuations (MGW-FL) of various nucleotide sequences for Engrailed (En) and Distal-less (Dll) proteins. (A) Representative structures of the Dll and En protein–DNA complexes derived from MD simulations. Minor groove (MG)-interacting residues are labeled and shown as sticks. (B) Average MGW-FL profiles derived from MD simulations for three DNA sequences of different affinities (High, Medium, and Low affinity). For each sequence, MGW-FL is plotted for DNA bound to Dll (orange), En (blue), and unbound DNA (gray). (C) Residue–DNA contact maps for MG-interacting residues identified in (A), showing the closest DNA positions used to determine the residue labels in (B) (see Materials and Methods for details).

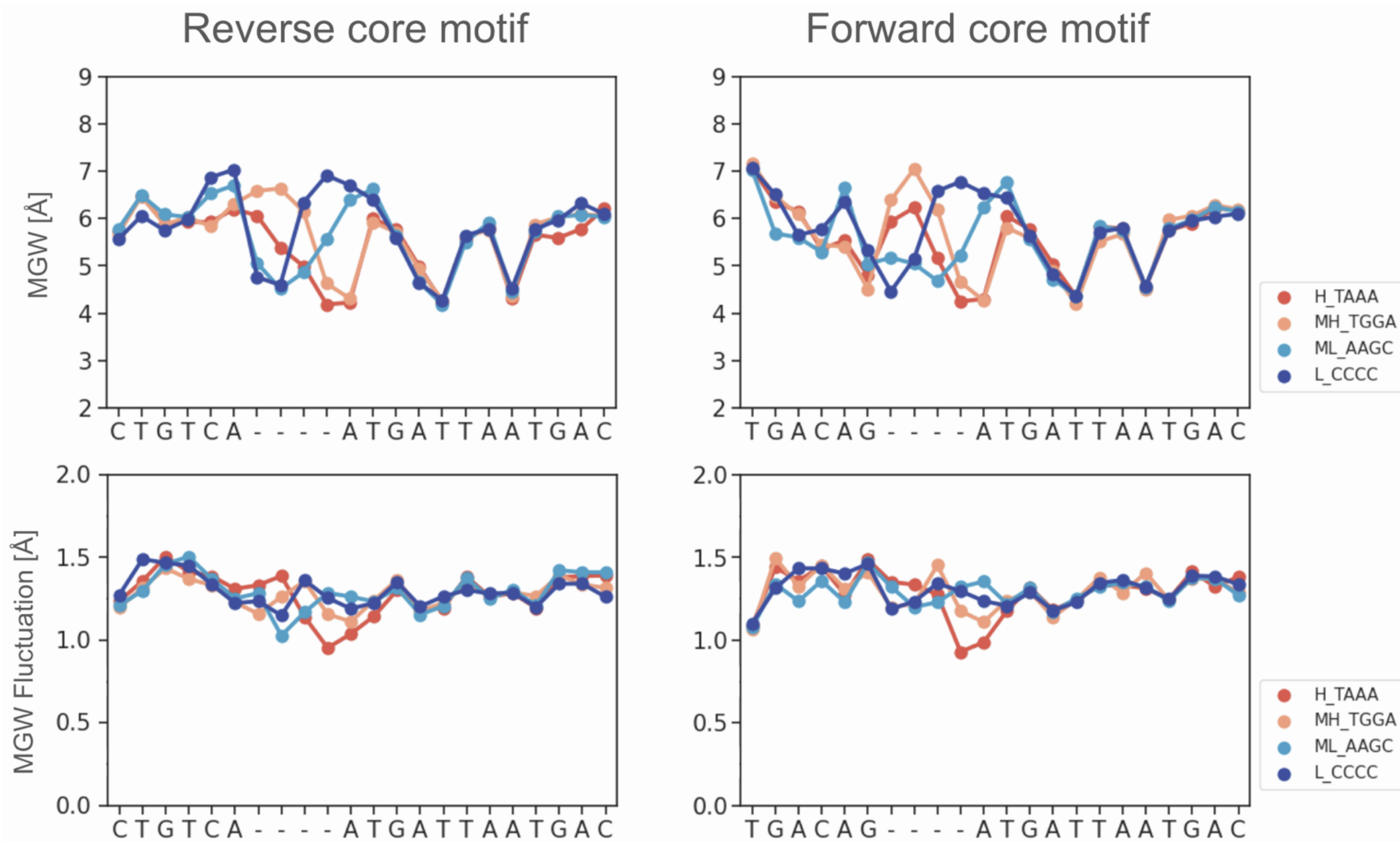

Fig. S5: Average unbound MGW and MGW-FL for four DNA sequences of various affinities for sequences with reverse core motif and sequences with forward core motif from MD simulations.

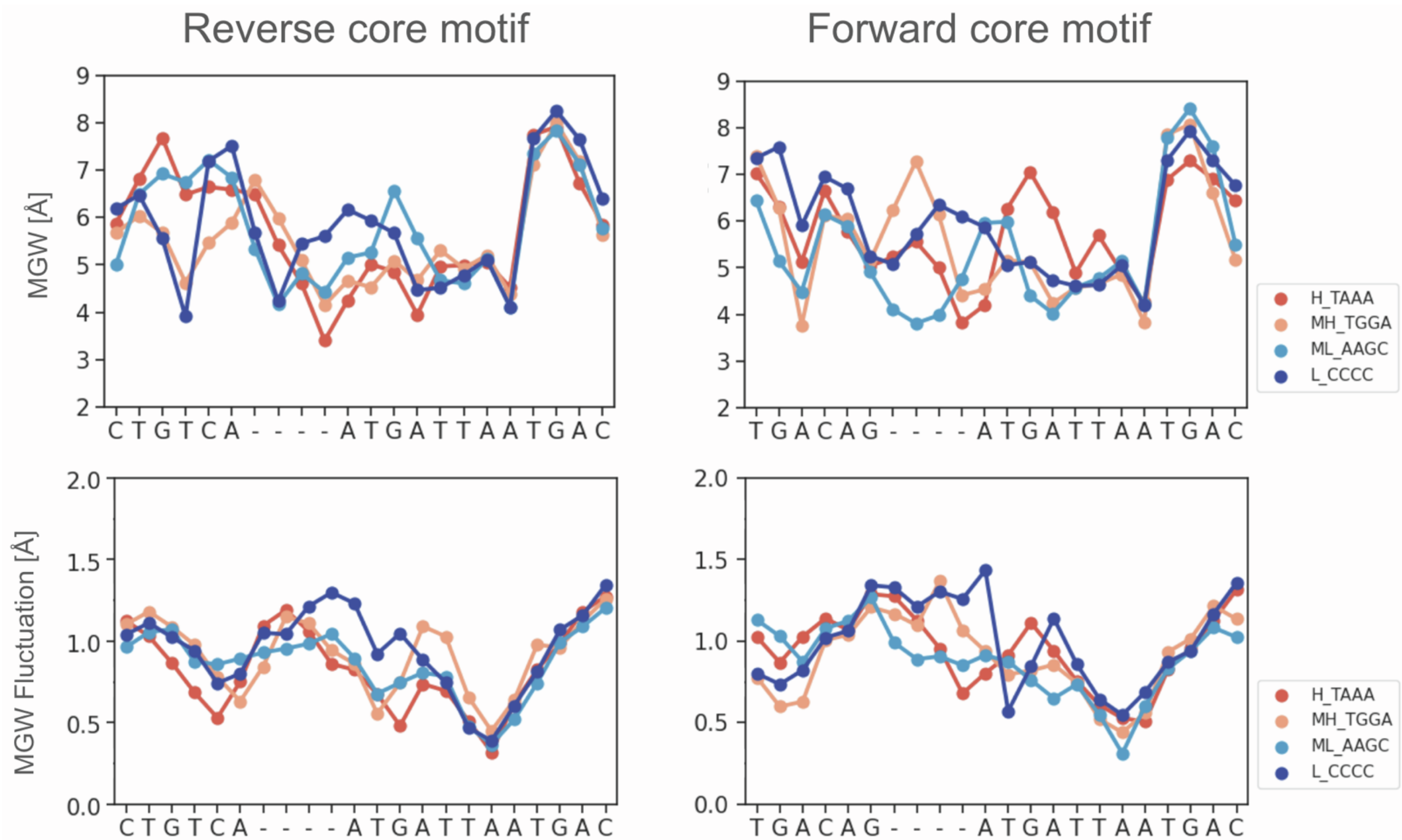

Fig. S6: Average MGW and MGW-FL for bound DNA fragments of various affinities calculated from MD simulations for sequences with reverse core motif and sequences with forward core motif.

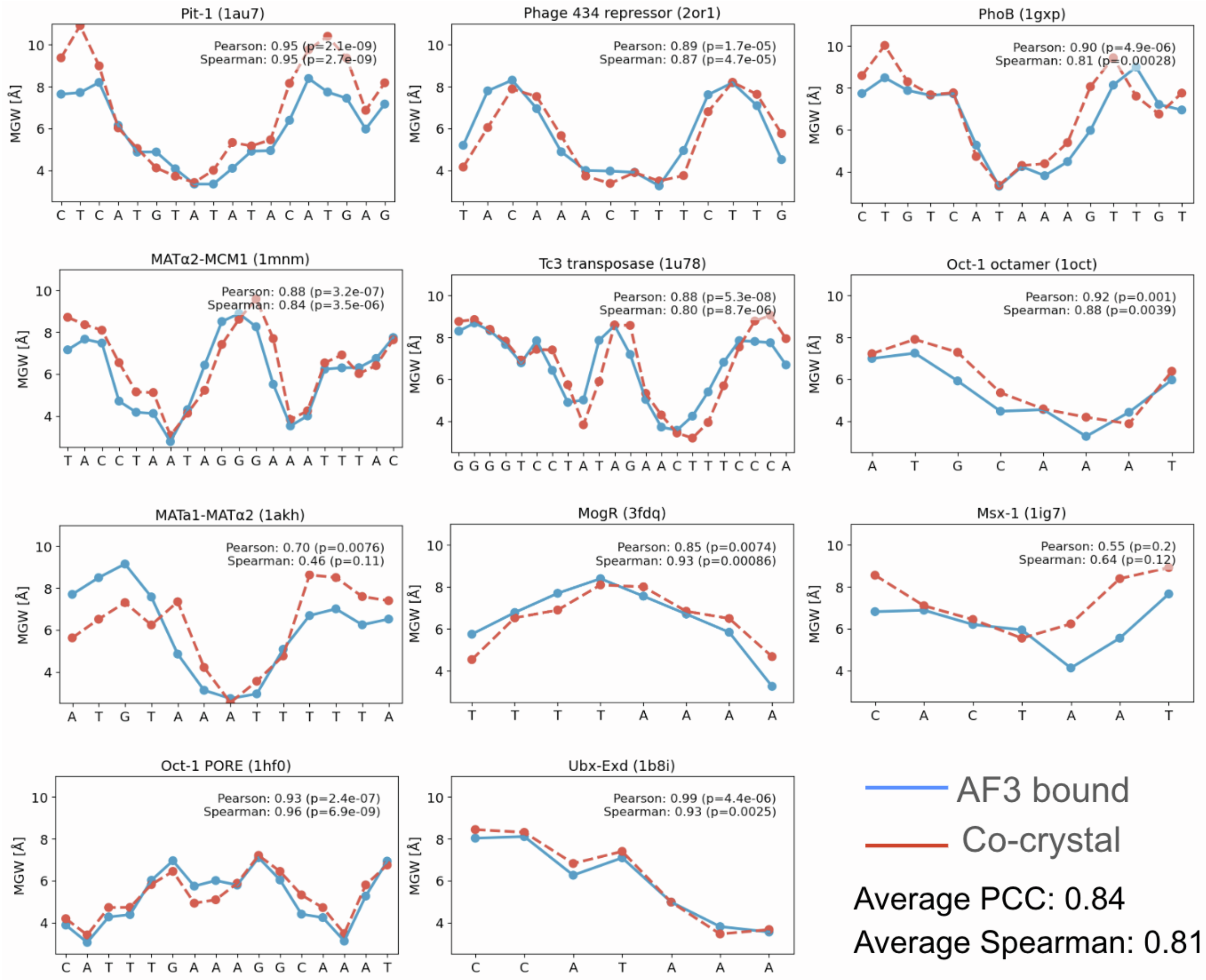

Fig. S7: MGW comparison for complexes predicted with AlphaFold 3 (AF3) (2) (blue) and co-crystal structures (red) for eleven selected protein–DNA complexes obtained from (3).

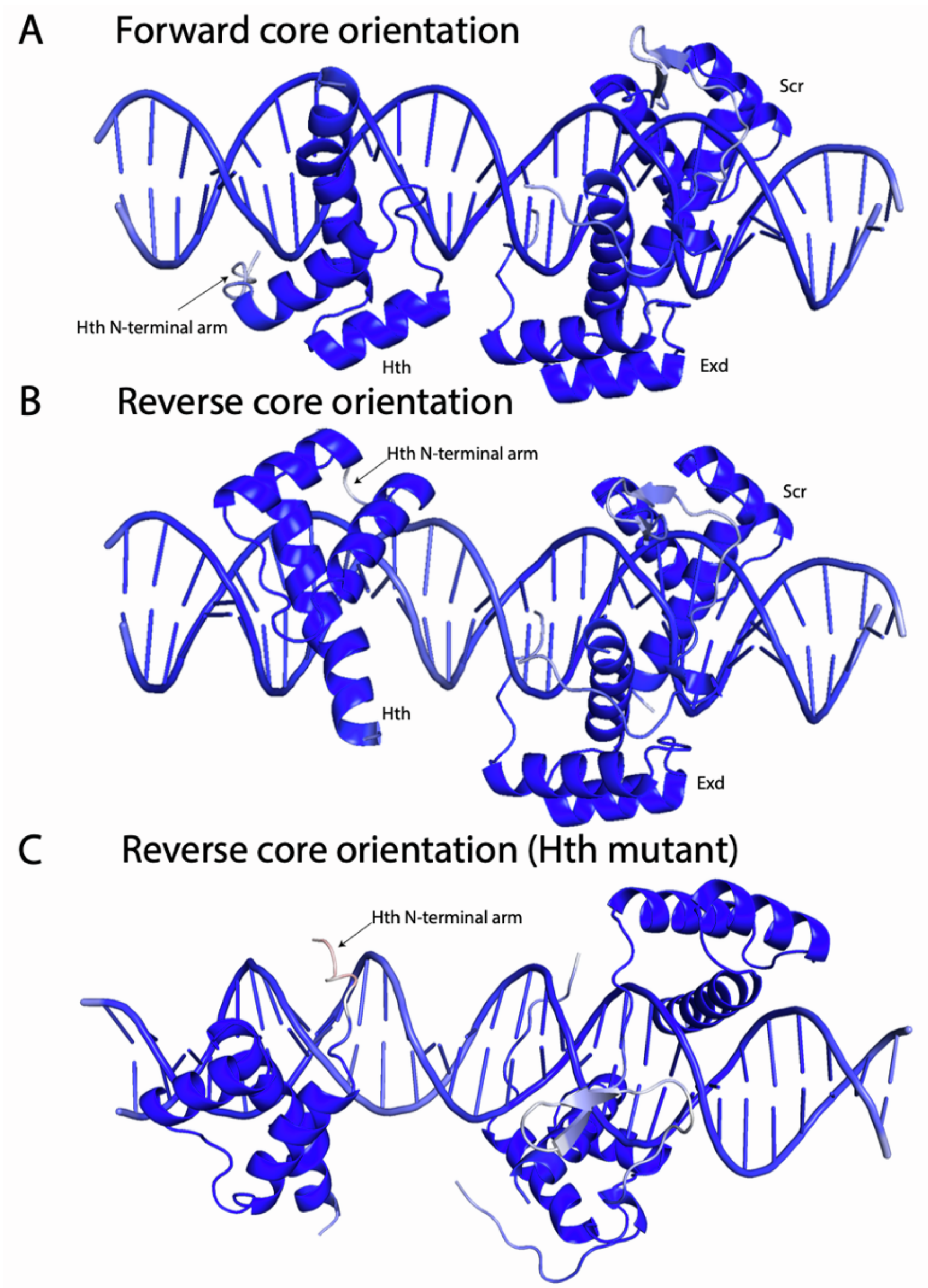

Fig. S8: AF3 prediction for Hth complex with forward (A), reverse core orientations (B), and Hth mutant (C). The structures are colored based on AF3 pLDDT scores (blue color implying higher AF3 confidence within the standard 0 to 100 range). Arrow indicates the mutated N-terminal arm residues with reduced pLDDT score.

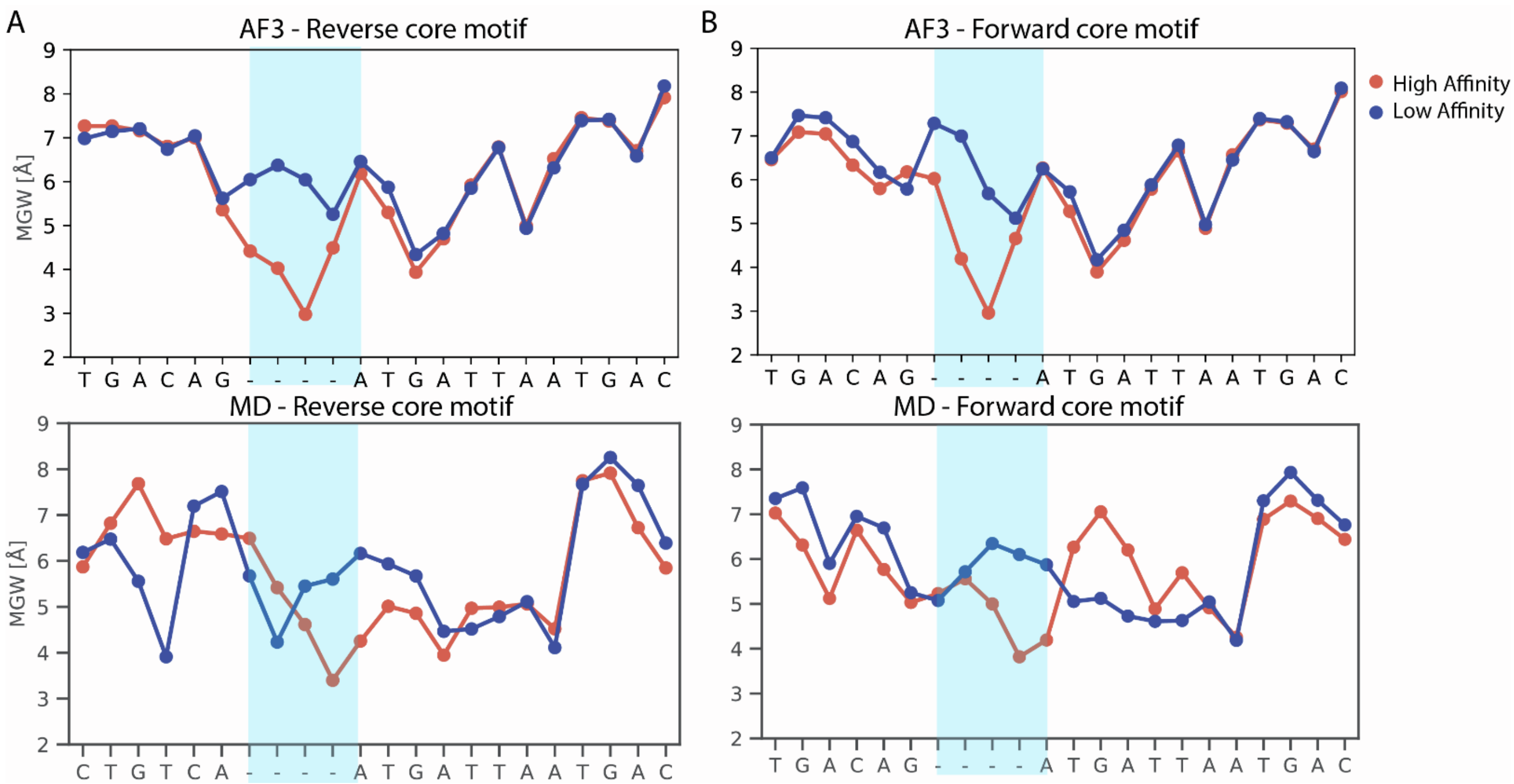

Fig. S9: MGW comparison between AF3-derived predictions (top panel) and MD simulations (bottom panel) for high-affinity and low-affinity DNA sequences in both (A) forward and (B) reverse core motif orientations.

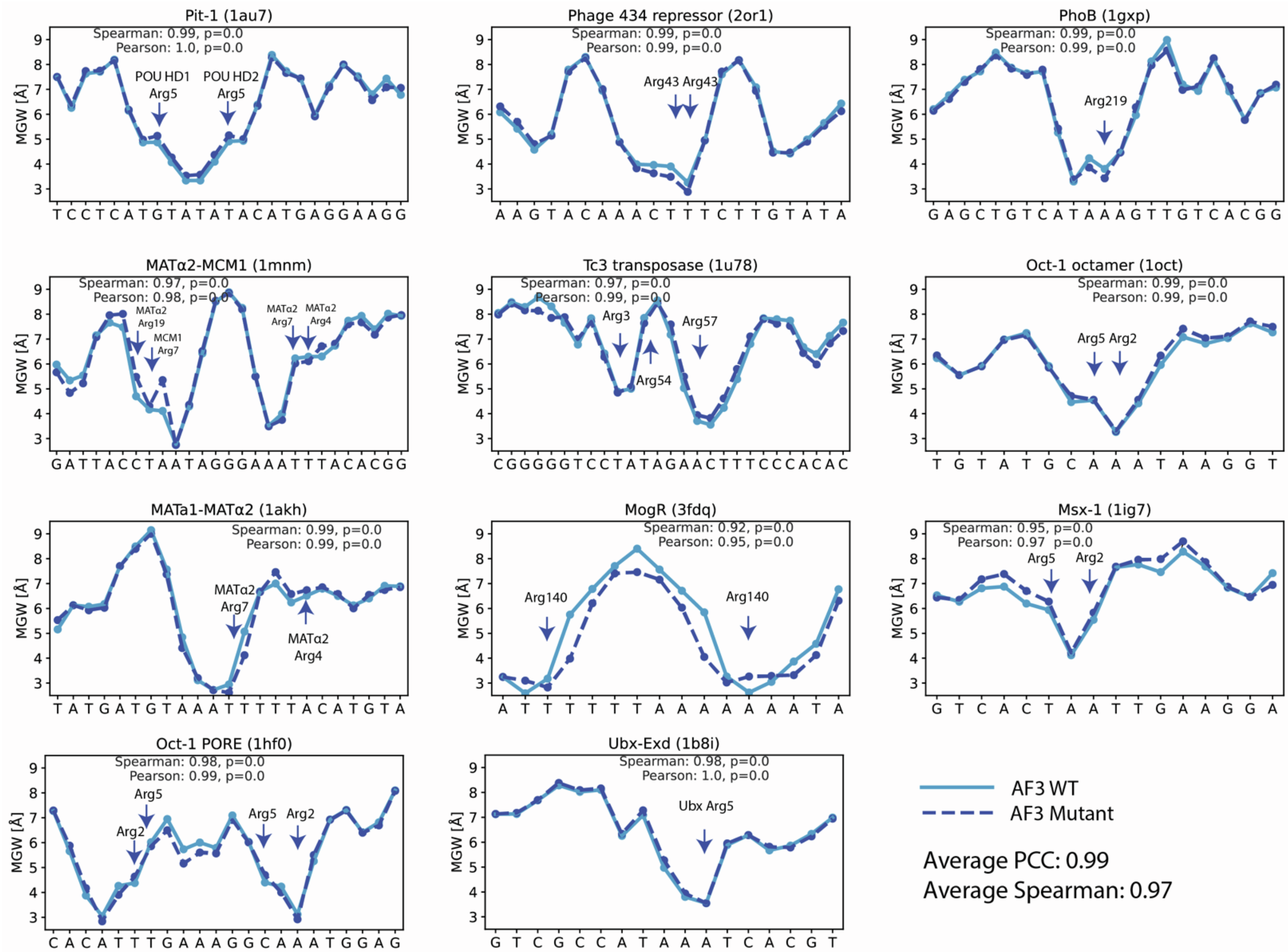

Fig. S10: MGW comparison for AF3-predicted wild-type (WT) (light blue solid line) and AF3-predicted mutant (darker blue dashed line) structures for eleven selected protein–DNA complexes obtained from (3). The mutated residues are annotated with arrows.

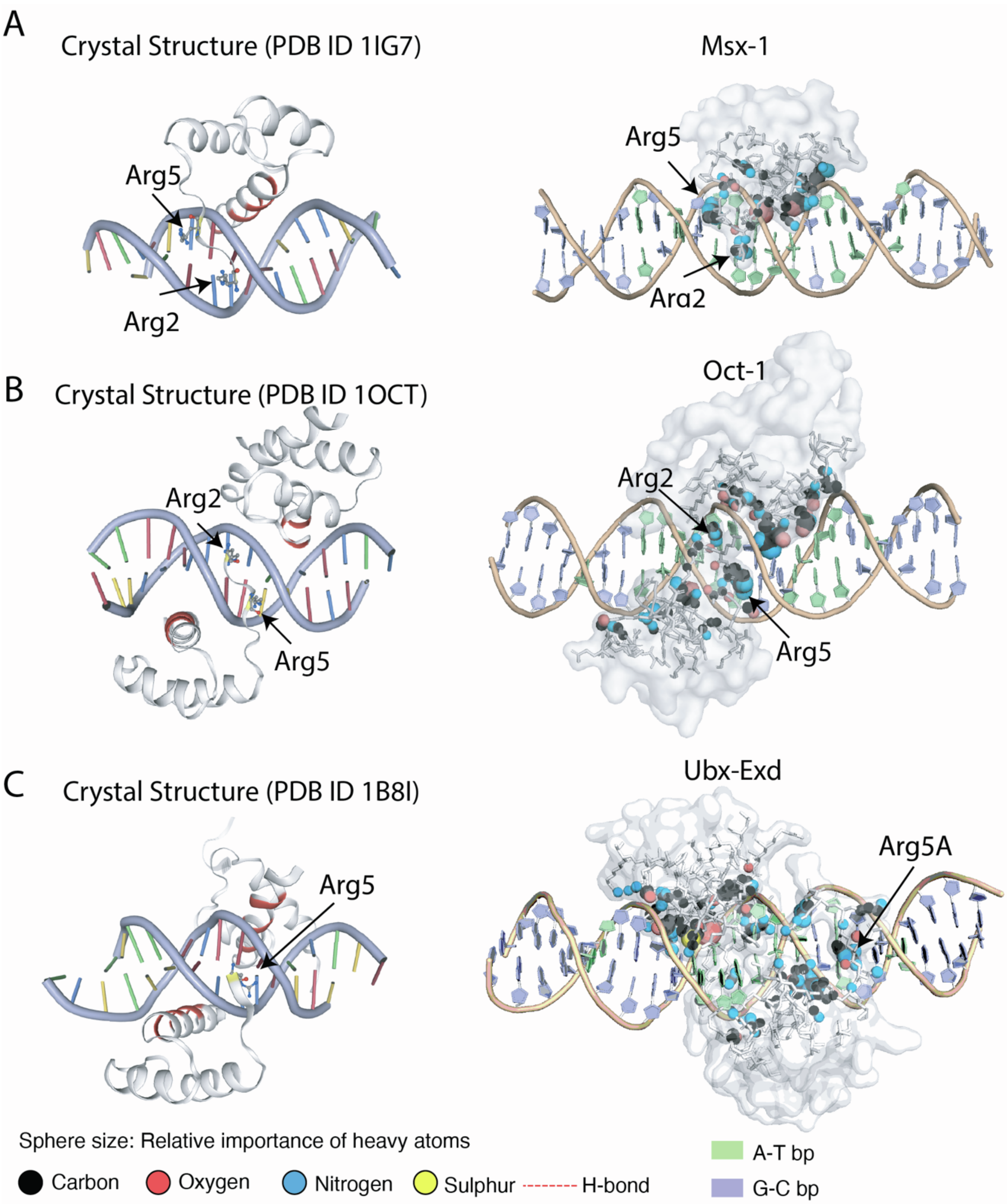

Fig. S11: DeepPBS (4) heavy-atom relative importance (RI) scores for various AF3-predicted wild-type (WT) homeodomain–DNA complexes. Sphere sizes reflect the relative importance of each heavy atom. Key minor groove (MG) contacts are annotated for these protein–DNA complexes. Visualizations of the co-crystal structures are shown on the left, and DeepPBS RI scores are shown on the right.

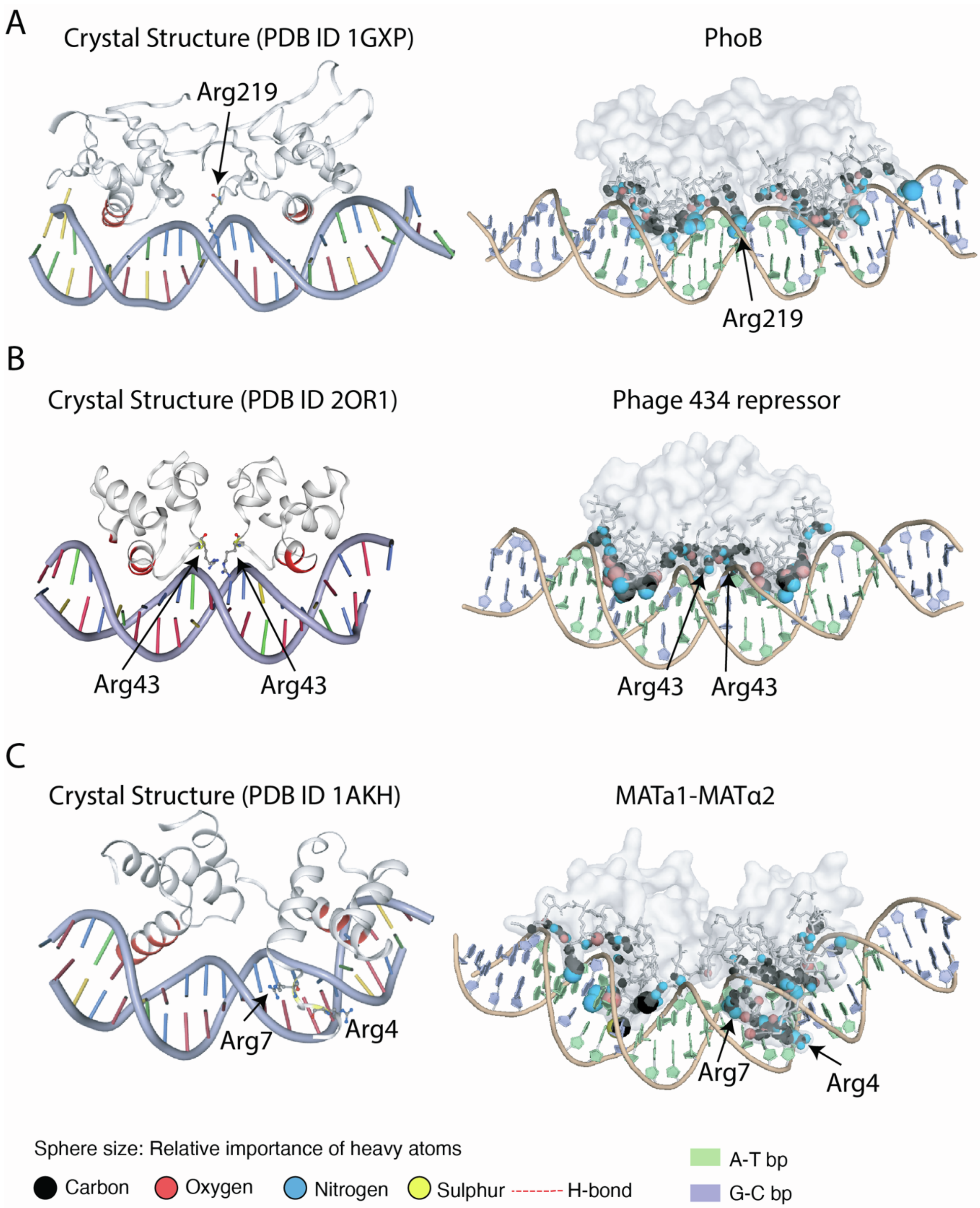

Fig. S12: DeepPBS (4) heavy-atom relative importance (RI) scores for various AF3-predicted wild-type (WT) protein–DNA complexes. Sphere sizes reflect the relative importance of each heavy atom. Key minor groove (MG) contacts are annotated for these protein–DNA complexes. Visualizations of the co-crystal structures are shown on the left, and DeepPBS RI scores are shown on the right.

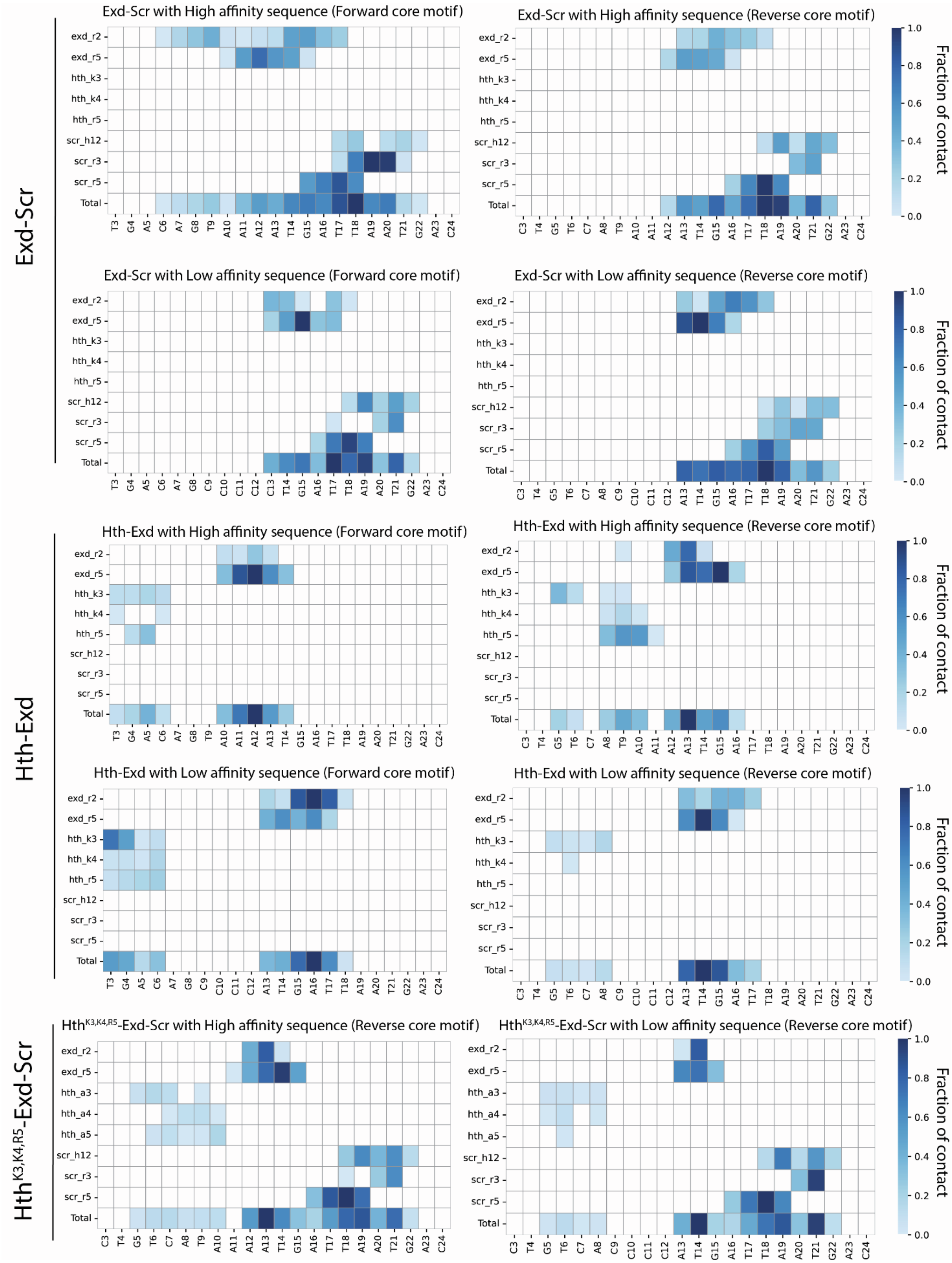

Fig. S13: Minor groove contacts for high- and low-affinity sequences across various protein–DNA complex architectures. The complexes analyzed include the Hth-Exd–DNA complex, the Exd-Scr–DNA complex, and the mutant Hth^{K3A,K4A,R5A}-Exd–DNA complex. DNA sequences contain both forward and reverse core motifs.

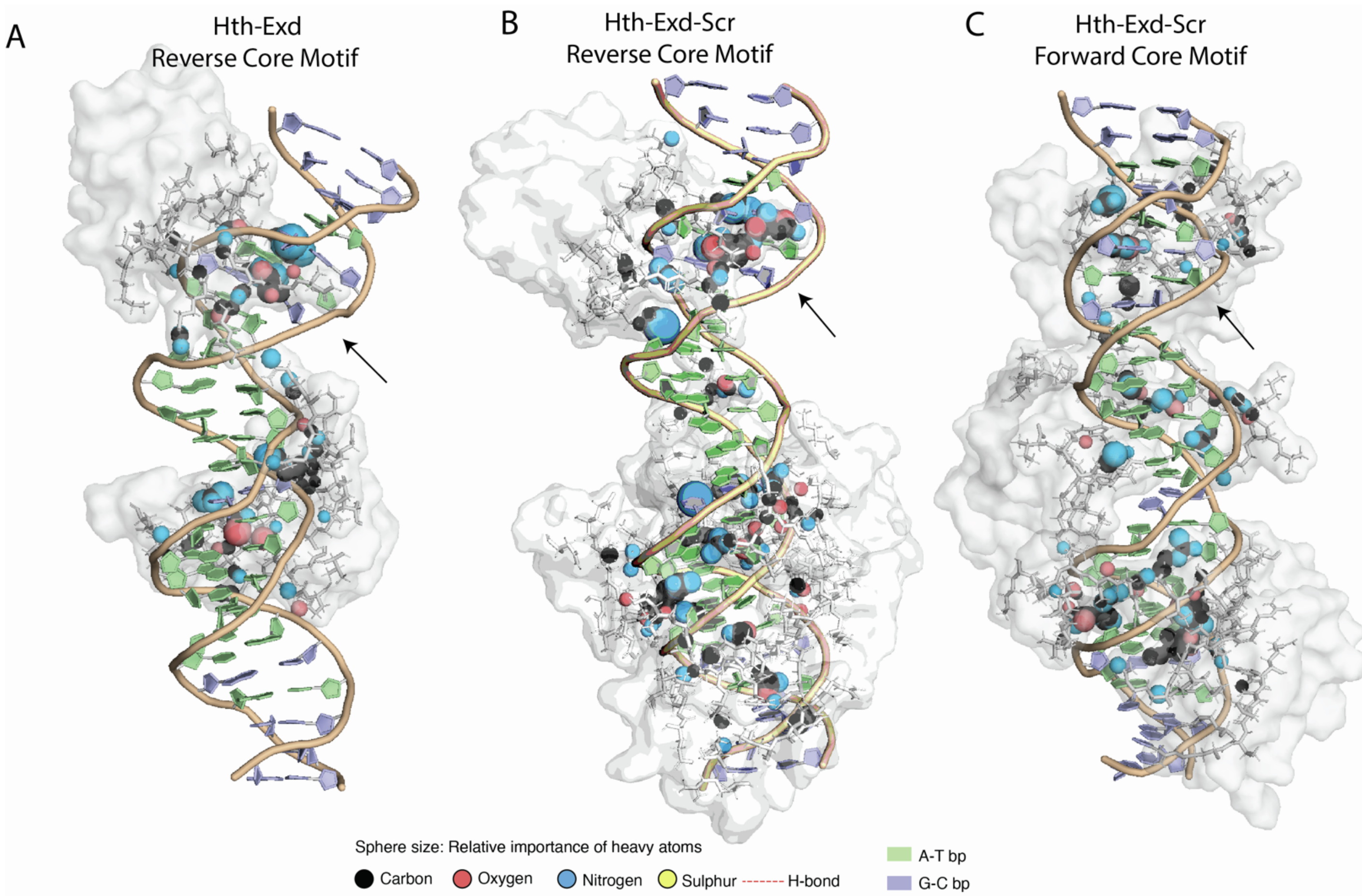

Fig. S14: DeepPBS relative importance (RI) analysis of Hth-Exd-Scr and Hth-Exd complexes. Sphere sizes represent the RI scores of heavy atoms in mediating protein–DNA interactions, with a black arrow indicating the Hth core motif region. (A) Hth-Exd complex with DNA in the reverse core motif orientation, (B) Hth-Exd-Scr complex with DNA in the reverse orientation, and (C) Hth-Exd-Scr complex with DNA in the forward orientation.

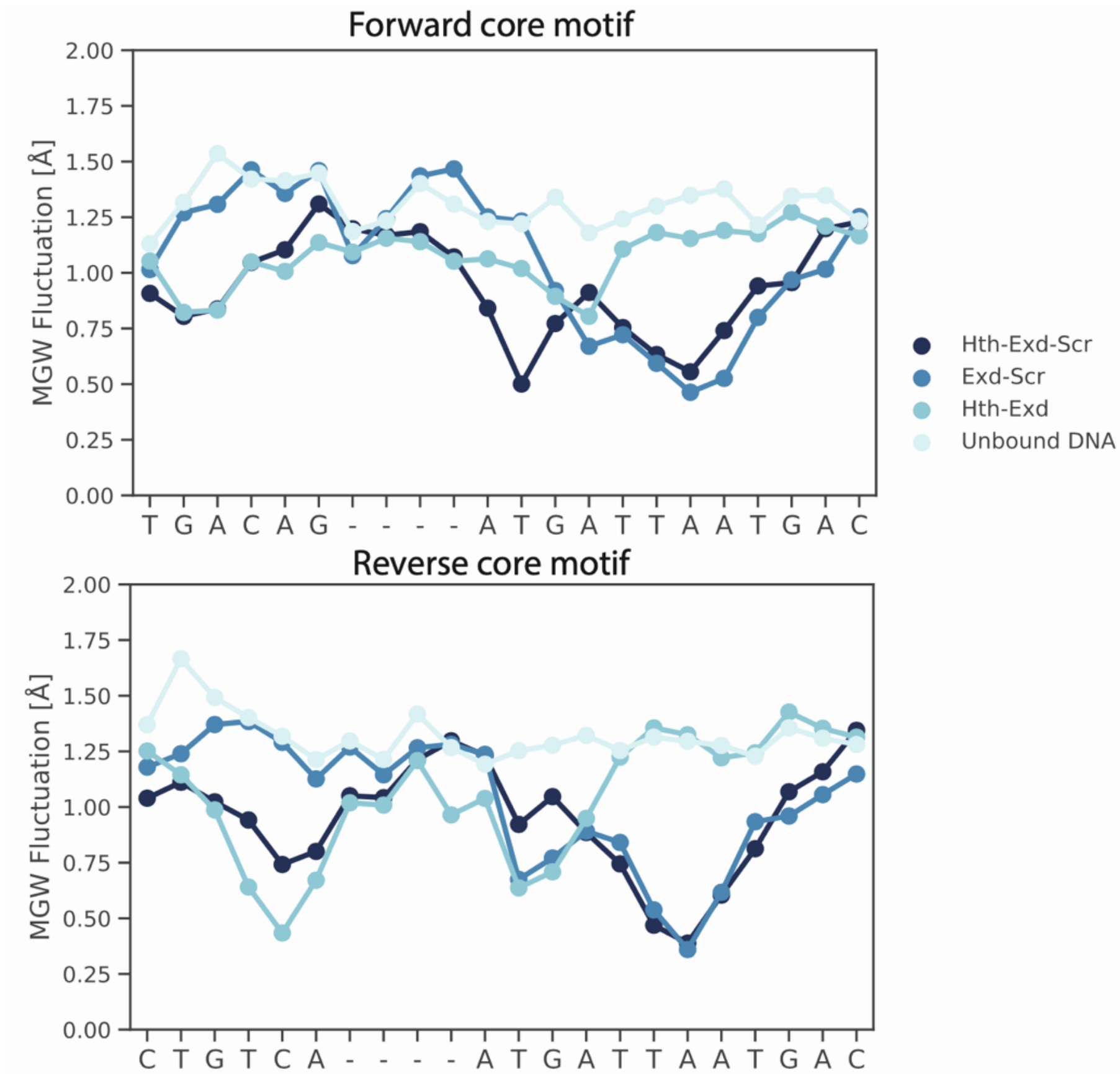

Fig. S15: MGW-FL of Hth-Exd-Scr, Hth-Exd, and Exd-Scr complexes bound to low-affinity nucleotide sequences with core motifs in (A) forward versus (B) reverse core orientations. Unbound DNA is also shown.

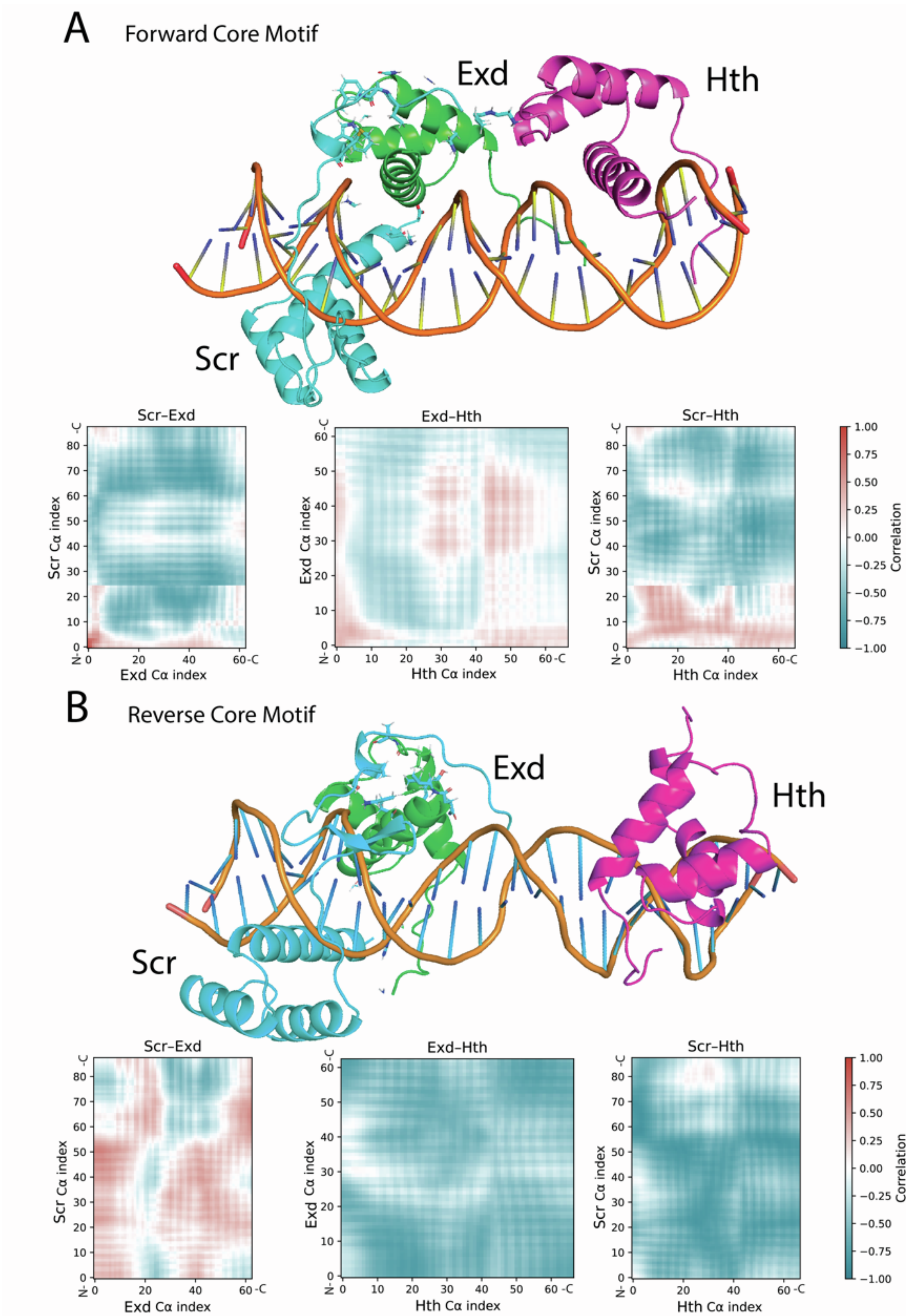

Fig. S16: Replica-averaged dynamic cross-correlation matrices (DCCMs) for the Hth-Exd-Scr trimer in the (A) forward and (B) reverse orientations. Positive values indicate coordinated motion, and negative values indicate anticorrelated motion. The patterns reveal orientation-dependent coupling between Scr-Exd, Exd-Hth, and Scr-Hth that reflects both direct contacts and DNA-mediated correlation within the trimer.

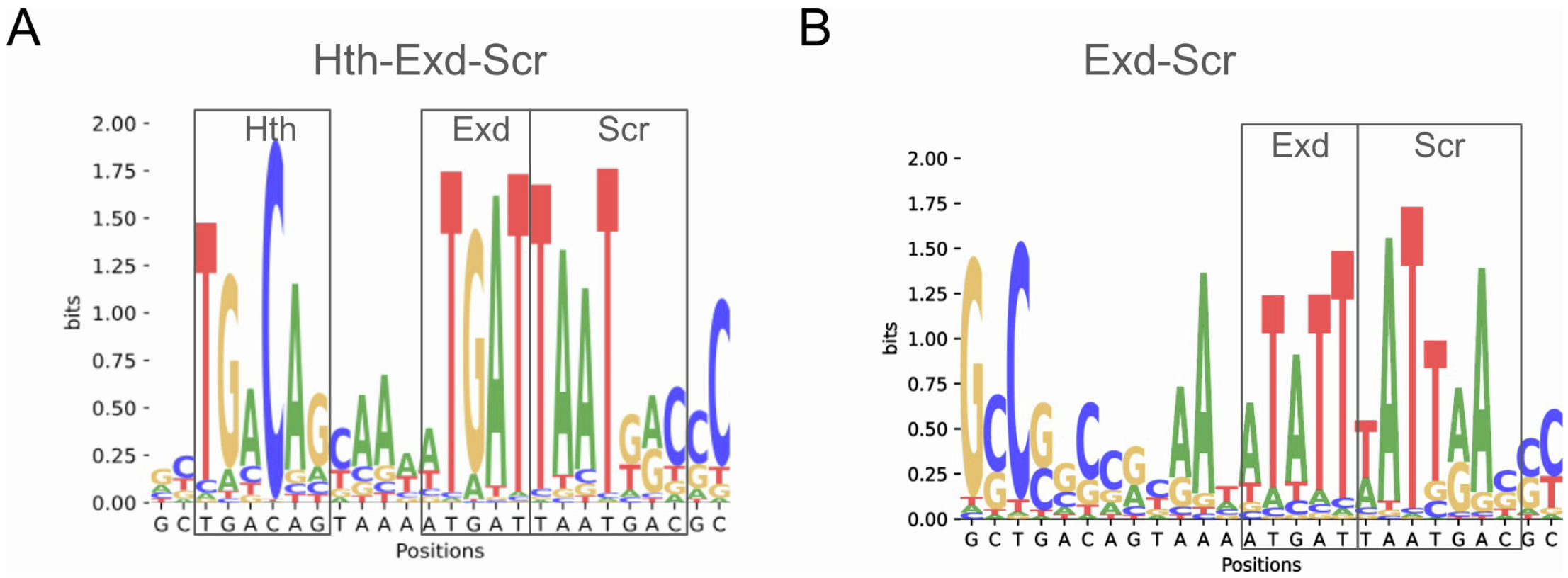

Fig. S17: DNA binding specificity predicted by DeepPBS for the Hth-Exd-Scr trimer with high- and low-affinity sequences derived from SELEX-seq experiments (Table S1) (5).

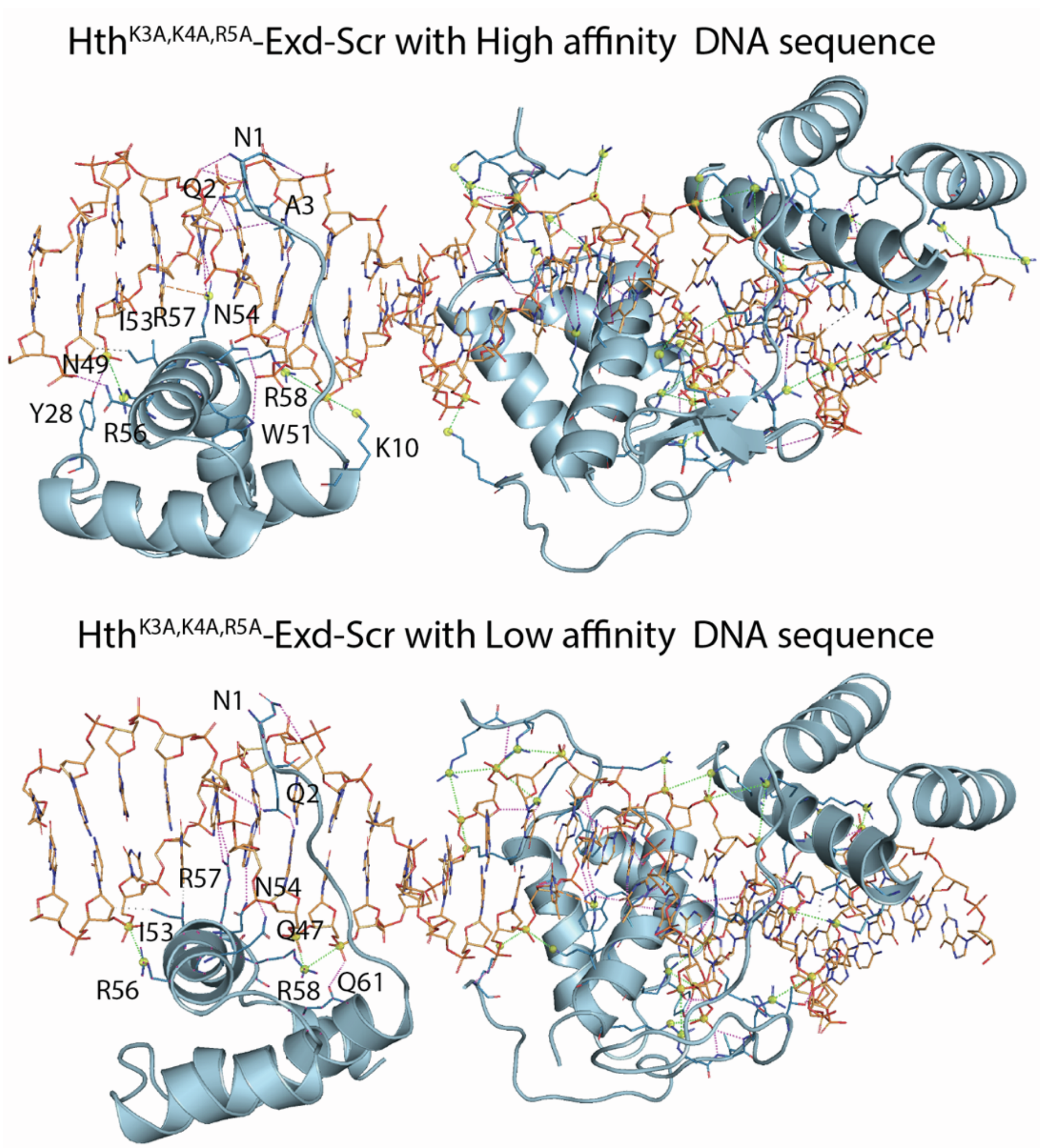

Fig. S18: DNA interactions of the Hth-Exd-Scr trimer with high- and low-affinity nucleotide sequences. Magenta lines indicate hydrogen bonding, orange dashes indicate π-cation interaction, gray indicate hydrophobic interaction, and green dashes indicate salt bridges.

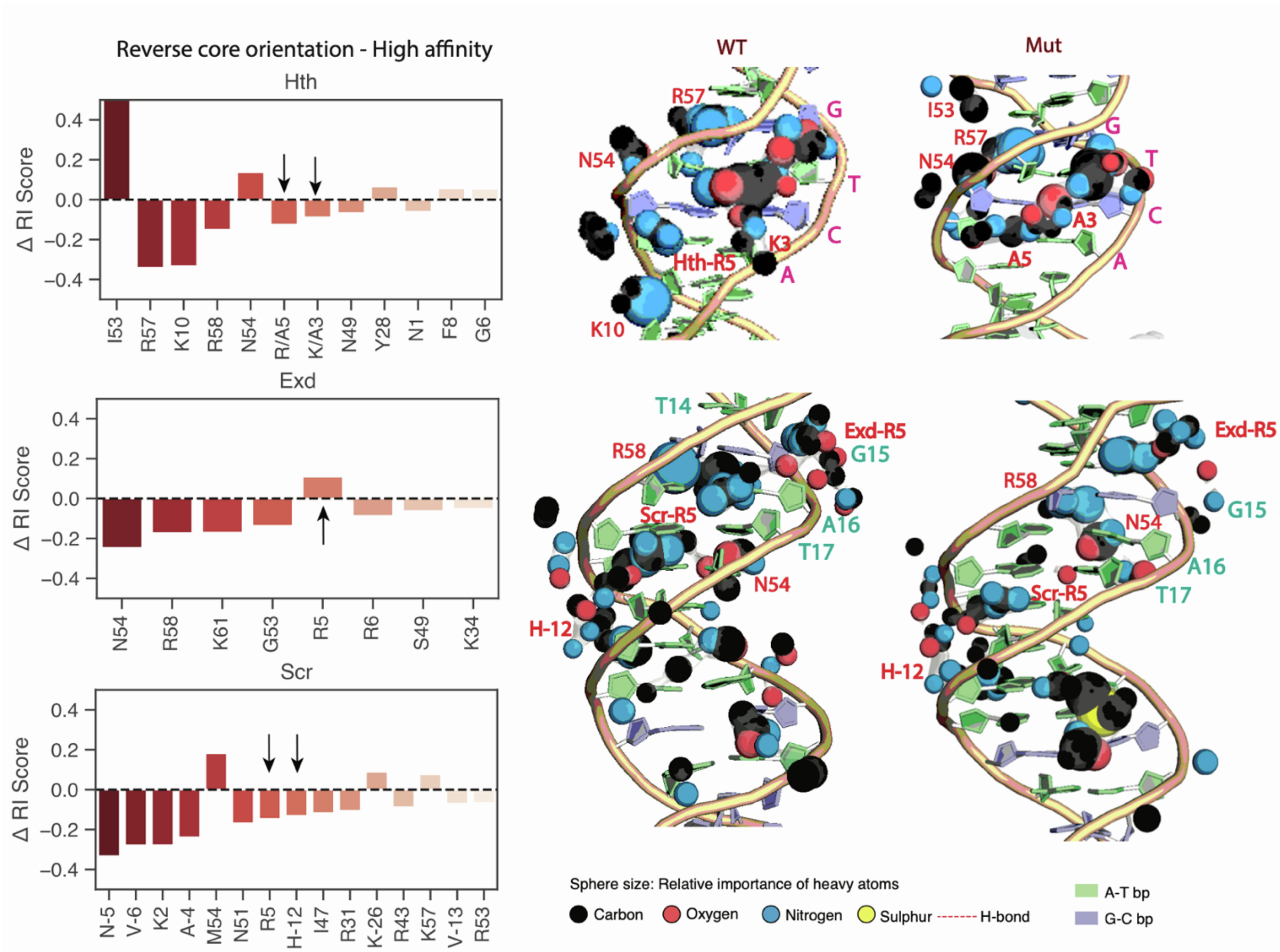

Fig. S19: Change in relative importance (RI) scores for the Hth-Exd-Scr–DNA ternary complex with high-affinity nucleotide sequence upon mutation of Hth residues. RI changes are computed as follows: $\Delta RI = RI_{\text{Mutant}} - RI_{\text{WT}}$. Arrows highlight residues with MG contacts of each protein. The right panel shows visualizations of DeepPBS-predicted RI scores for wild-type (WT) and mutant (Mut) complexes, with key residues labeled. Protein omitted for visibility.

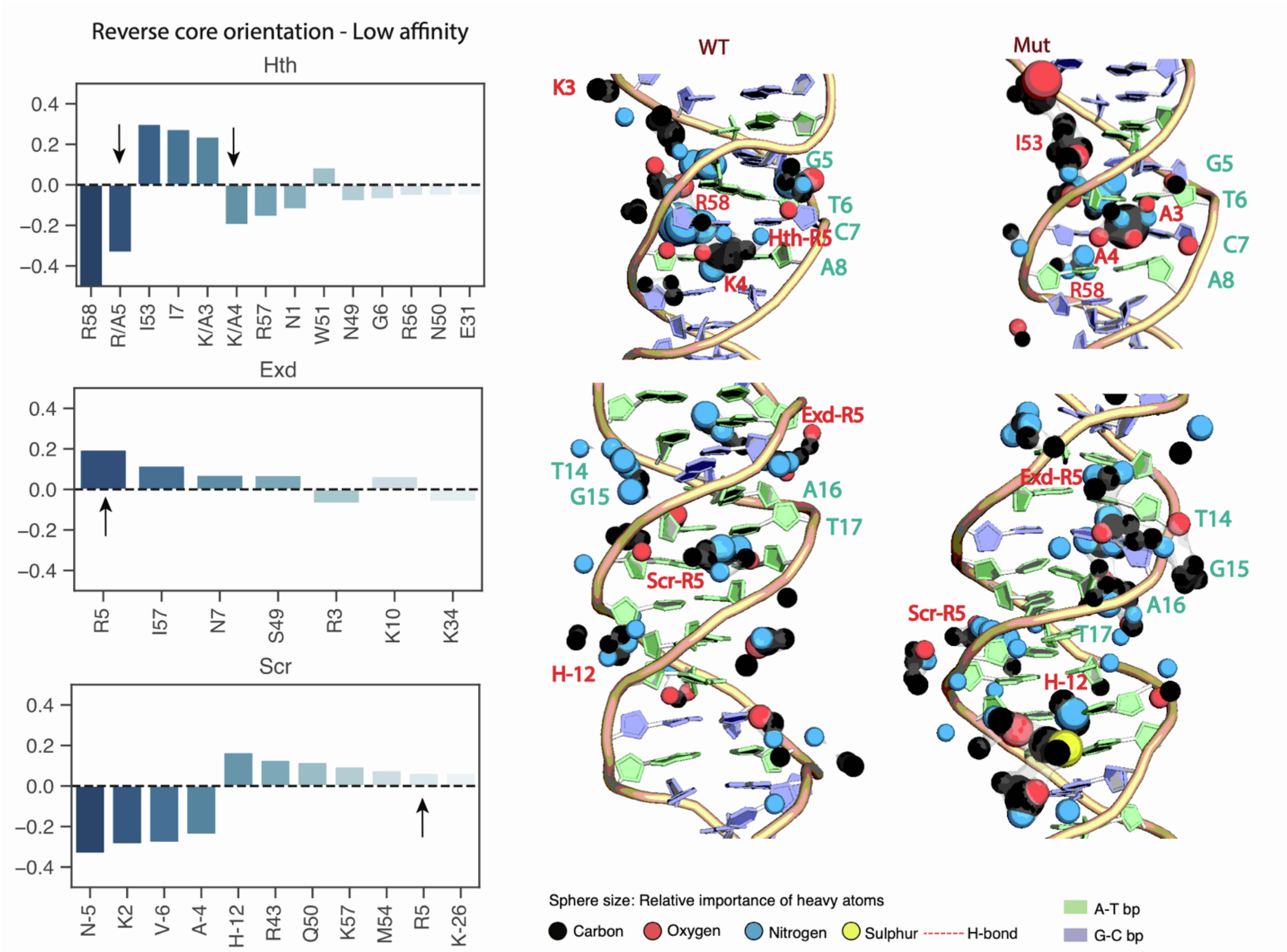

Fig. S20: Change in relative importance (RI) scores for the Hth-Exd-Scr–DNA complex with low-affinity sequence upon mutation of specific Hth residues. RI changes are computed as follows: $\Delta RI = RI_{\text{Mutant}} - RI_{\text{WT}}$. Arrows highlight residues that interact with the minor groove. The right panel shows visualizations of DeepPBS-derived RI scores for wild-type (WT) and mutant (Mut) complexes, with key residues labeled. Protein omitted for visibility.

SUPPLEMENTAL TABLES

Affinity	Sequence
High - Forward	$C_1G_2T_3G_4A_5C_6A_7G_8\mathbf{T_9A_{10}A_{11}A_{12}}A_{13}T_{14}G_{15}A_{16}T_{17}T_{18}A_{19}A_{20}T_{21}G_{22}A_{23}C_{24}C_{25}G_{26}$
Medium High -Forward	$C_1G_2T_3G_4A_5C_6A_7G_8\mathbf{T_9G_{10}G_{11}A_{12}}A_{13}T_{14}G_{15}A_{16}T_{17}T_{18}A_{19}A_{20}T_{21}G_{22}A_{23}C_{24}C_{25}G_{26}$
Medium Low -Forward	$C_1G_2T_3G_4A_5C_6A_7G_8\mathbf{T_9A_{10}A_{11}G_{12}C}_{13}T_{14}G_{15}A_{16}T_{17}T_{18}A_{19}A_{20}T_{21}G_{22}A_{23}C_{24}C_{25}G_{26}$
Low - Forward	$C_1G_2T_3G_4A_5C_6A_7G_8\mathbf{C_9C_{10}C_{11}C_{12}}A_{13}T_{14}G_{15}A_{16}T_{17}T_{18}A_{19}A_{20}T_{21}G_{22}A_{23}C_{24}C_{25}G_{26}$
High - Reverse	$C_1G_2C_3T_4G_5T_6C_7A_8\mathbf{T_9A_{10}A_{11}A_{12}}A_{13}T_{14}G_{15}A_{16}T_{17}T_{18}A_{19}A_{20}T_{21}G_{22}A_{23}C_{24}C_{25}G_{26}$
Medium High -Reverse	$C_1G_2C_3T_4G_5T_6C_7A_8\mathbf{T_9G_{10}G_{11}A_{12}}A_{13}T_{14}G_{15}A_{16}T_{17}T_{18}A_{19}A_{20}T_{21}G_{22}A_{23}C_{24}C_{25}G_{26}$
Medium Low -Reverse	$C_1G_2C_3T_4G_5T_6C_7A_8\mathbf{T_9A_{10}A_{11}G_{12}C}_{13}T_{14}G_{15}A_{16}T_{17}T_{18}A_{19}A_{20}T_{21}G_{22}A_{23}C_{24}C_{25}G_{26}$
Low - Reverse	$C_1G_2C_3T_4G_5T_6C_7A_8\mathbf{C_9C_{10}C_{11}C_{12}}A_{13}T_{14}G_{15}A_{16}T_{17}T_{18}A_{19}A_{20}T_{21}G_{22}A_{23}C_{24}C_{25}G_{26}$

Table S1: Nucleotide sequences used for MD simulation of the tertiary Hth-Exd-Scr–DNA complex. Spacer sequences derived from SELEX-seq experiments using the Top-Down Crawl (1) method are shown in bold.

Scr 88 *aa*	GKKNPPQIYPWMKRVHLGTSTVNANGETKRQRTSYTRYQTLELEKEFHFNRYLTRRRRIEIAH ALCLTERQIKIWFQNRRMKWKKEHK
Exd 63 *aa*	ARRKRRNFSKQASEILNEYFYSHLSNPYPSEEAKEELARKCGITVSQVSNWFGNKRIRYKKNI
Hth 67 *aa*	NQKKRGIFPKVATNILRAWLFQHLTHPYPSEDQKKQLAQDTGLTILQVNNWFINARRRIVQPMIDQS
Hth mutant 67 *aa*	NQAAAGIFPKVATNILRAWLFQHLTHPYPSEDQKKQLAQDTGLTILQVNNWFINARRRIVQPMIDQS

Table S2: Protein sequences used for MD simulation of the tertiary Hth-Exd-Scr–DNA complex. The length of each protein is annotated below in number of amino acids (aa). The mutated residues in the $Hth^{K3A,K4A,R5}$ mutant is annotated with underline. Select N-terminal arm residues such as Scr H-12, Scr R3, Scr R5, Exd R2, Exd R5, Hth K3, Hth K4, and Hth R5 are highlighted in red.

ID	Type	Orient	DNA	Complex	Mut	Reps	Time (ns)
1	Bound	Forward	H_TAAA	Hth–Exd–Scr (trimer)	WT	3	500
2	Bound	Forward	MH_TGGA	Hth–Exd–Scr (trimer)	WT	3	500
3	Bound	Forward	ML_AAGC	Hth–Exd–Scr (trimer)	WT	3	500
4	Bound	Forward	L_CCCC	Hth–Exd–Scr (trimer)	WT	3	500
5	Bound	Forward	H_TAAA	Hth–Exd (dimer)	WT	3	500
6	Bound	Forward	L_CCCC	Hth–Exd (dimer)	WT	3	500
7	Bound	Forward	H_TAAA	Exd–Scr (dimer)	WT	3	500
8	Bound	Forward	L_CCCC	Exd–Scr (dimer)	WT	3	500
9	Bound	Reverse	H_TAAA	Hth–Exd–Scr (trimer)	WT	3	500
10	Bound	Reverse	MH_TGGA	Hth–Exd–Scr (trimer)	WT	3	500
11	Bound	Reverse	ML_AAGC	Hth–Exd–Scr (trimer)	WT	3	500
12	Bound	Reverse	L_CCCC	Hth–Exd–Scr (trimer)	WT	3	500
13	Bound	Reverse	H_TAAA	Hth–Exd (dimer)	WT	3	500
14	Bound	Reverse	L_CCCC	Hth–Exd (dimer)	WT	3	500
15	Bound	Reverse	H_TAAA	Exd–Scr (dimer)	WT	3	500
16	Bound	Reverse	L_CCCC	Exd–Scr (dimer)	WT	3	500
17	Bound	Reverse	H_TAAA	Hth–Exd–Scr (trimer)	Mutant	3	500
18	Bound	Reverse	L_CCCC	Hth–Exd–Scr (trimer)	Mutant	3	500
19	Bound	Reverse	H_TAAA	Hth–Exd (dimer)	Mutant	3	500
20	Bound	Reverse	L_CCCC	Hth–Exd (dimer)	Mutant	3	500
21	Unbound	Forward	H_TAAA	DNA only	WT	3	300
22	Unbound	Forward	MH_TGGA	DNA only	WT	3	300
23	Unbound	Forward	ML_AAGC	DNA only	WT	3	300
24	Unbound	Forward	L_CCCC	DNA only	WT	3	300
25	Unbound	Reverse	H_TAAA	DNA only	WT	3	300
26	Unbound	Reverse	MH_TGGA	DNA only	WT	3	300
27	Unbound	Reverse	ML_AAGC	DNA only	WT	3	300
28	Unbound	Reverse	L_CCCC	DNA only	WT	3	300
29	Bound	–	High	Engrailed	WT	3	300
30	Bound	–	Medium	Engrailed	WT	3	300
31	Bound	–	Low	Engrailed	WT	3	300
32	Bound	–	High	Distal-less	WT	3	300
33	Bound	–	Medium	Distal-less	WT	3	300
34	Bound	–	Low	Distal-less	WT	3	300

Table S3: Summary of all MDs simulations performed in this study. Each system was simulated in three independent replicas unless otherwise noted. Systems include forward and reverse orientations, four DNA core sequences (High, Medium High, Medium Low, Low), and multiple protein configurations (Hth-Exd-Scr trimer, Hth-Exd dimer, Exd-Scr dimer), as well as unbound DNA controls.

Comparison	Region	State A	State B	% Change	p-value
Unbound → Exd-Scr (Reverse)	Hth	1.35	1.27	-5.9%	0.263
Unbound → Exd-Scr (Reverse)	Spacer	1.22	1.12	-7.7%	0.275
Unbound → Exd-Scr (Reverse)	Exd_Scr	1.27	0.85	-33.2%	**0.002**
Exd-Scr → WT Trimer(Reverse)	Hth	1.27	0.91	-28.6%	**0.042**
Exd-Scr → WT Trimer(Reverse)	Spacer	1.12	1.03	-8.4%	0.357
Exd-Scr → WT Trimer(Reverse)	Exd_Scr	0.85	0.81	-4.7%	0.431
WT Trimer → Mutant(Reverse)	Hth	0.91	1.01	+11.7%	**0.049**
WT Trimer → Mutant(Reverse)	Spacer	1.03	1.08	+4.7%	0.740
WT Trimer → Mutant(Reverse)	Exd_Scr	0.81	0.81	-0.6%	0.935
Unbound → WT Trimer(Reverse)	Hth	1.35	0.91	-32.8%	**0.003**
Unbound → WT Trimer(Reverse)	Spacer	1.22	1.03	-15.4%	**0.033**
Unbound → WT Trimer(Reverse)	Exd_Scr	1.27	0.81	-36.4%	**0.003**
Unbound → Hth-Exd (Reverse)	Hth	1.35	0.91	-32.7%	**0.008**
Unbound → Hth-Exd (Reverse)	Spacer	1.22	1.09	-10.2%	0.342
Unbound → Hth-Exd (Reverse)	Exd_Scr	1.27	1.15	-10.1%	**0.030**
Unbound → Mutant(Reverse)	Hth	1.35	1.01	-25.0%	**0.020**
Unbound → Mutant(Reverse)	Spacer	1.22	1.08	-11.5%	0.376
Unbound → Mutant(Reverse)	Exd_Scr	1.27	0.81	-36.7%	**0.008**
Unbound → Exd-Scr (Forward)	Hth	1.33	1.32	-0.9%	0.731
Unbound → Exd-Scr (Forward)	Spacer	1.25	1.11	-11.4%	**0.030**
Unbound → Exd-Scr (Forward)	Exd_Scr	1.28	0.87	-32.3%	**0.013**
Exd-Scr → WT Trimer(Forward)	Hth	1.32	0.98	-25.5%	**0.042**
Exd-Scr → WT Trimer(Forward)	Spacer	1.11	1.08	-3.0%	0.598
Exd-Scr → WT Trimer(Forward)	Exd_Scr	0.87	0.83	-4.3%	0.638
Unbound → WT Trimer(Forward)	Hth	1.33	0.98	-26.2%	0.074
Unbound → WT Trimer(Forward)	Spacer	1.25	1.08	-14.0%	0.062
Unbound → WT Trimer(Forward)	Exd_Scr	1.28	0.83	-35.3%	**0.003**
Unbound → Hth-Exd (Forward)	Hth	1.33	1.05	-21.1%	0.056
Unbound → Hth-Exd (Forward)	Spacer	1.25	1.07	-14.8%	**0.004**
Unbound → Hth-Exd (Forward)	Exd_Scr	1.28	1.14	-11.4%	0.058

Table S4. Mean MGW-FL for the Hth core motif, spacer, and Exd–Scr core motif regions across simulated complexes. Comparisons include transitions from unbound DNA to dimers, wild-type trimer, and mutant trimer configurations in both reverse and forward orientations. State A and State B correspond to the first and second conditions listed for each comparison. Percent change reflects the relative difference in MGW fluctuation between states, and *p*-values are derived from two-tailed paired t-tests calculated across simulation replicates pooled across affinity classes. *P*-values < 0.05 are highlighted in bold.

SUPPLEMENTAL REFERENCES

1. Cooper, B., T.P. Chiu, and R. Rohs, 2022. Top-Down Crawl: a method for the ultra-rapid and motif-free alignment of sequences with associated binding metrics. *Bioinformatics*, **38**:5121–5123.
2. Abramson, J., J. Adler, J. Dunger, R. Evans, T. Green, A. Pritzel, O. Ronneberger, L. Willmore, A.J. Ballard, J. Bambrick, et al., 2024. Accurate structure prediction of biomolecular interactions with AlphaFold 3. *Nature*, **630**:493–500.
3. Azad, R.N., D. Zafiropoulos, D. Ober, Y. Jiang, T.P. Chiu, J.M. Sagendorf, R. Rohs, and T.D. Tullius, 2018. Experimental maps of DNA structure at nucleotide resolution distinguish intrinsic from protein-induced DNA deformations. *Nucleic Acids Res.*, **46**:2636–2647.
4. Mitra, R., J. Li, J.M. Sagendorf, Y. Jiang, A.S. Cohen, T.P. Chiu, C.J. Glasscock, and R. Rohs, 2024. Geometric deep learning of protein–DNA binding specificity. *Nat. Methods*, **21**:1674–1683.
5. Kribelbauer, J.F., R. Loker, S. Feng, C. Rastogi, N. Abe, H. Rube, H.J. Bussemaker, and R.S. Mann, 2020. Context-dependent gene regulation by homeodomain transcription factor complexes revealed by shape-readout deficient proteins. *Mol. Cell*, **78**:152–167.
